# RhoA WT allele loss unleashes the cancer driver function of RhoA E40Q

**DOI:** 10.1172/JCI201975

**Published:** 2026-08-25

**Authors:** Justine Noujarède, Qiuyue Wang, Eleftherios Panagiotis Kokkinogenis, Yuewan Luo, Ivona Cudina, Simon Willaume, Clémence Mooser, Lap Phuoc Nguyen, Mads Frederik Poulsen, Simon Heijmerikx, Thu Han Le Phan, Xiubin He, Jesper Bøje Andersen, Claus Storgaard Sørensen, Cord Brakebusch

**Affiliations:** University of Copenhagen, Biotech Research and Innovation Center (BRIC), Copenhagen, Denmark.

**Keywords:** Cell biology, Oncology, Cancer

## Abstract

Cancer hot spot mutations of unknown function often obscure the functional understanding of the molecular pathways underlying cancer formation and limit the progress of precision medicine. We investigated unresolved driver functions of *RHOA* in head and neck squamous cell carcinoma. Our investigation reveals that *RHOA* E40Q is a partial loss-of-function allele that paradoxically promotes tumorigenesis only in the absence of WT RhoA. Therefore, mice expressing *RHOA* E40Q specifically in keratinocytes lacking WT *Rhoa* spontaneously developed squamous cell carcinoma and had defective hair shaft formation. Mechanistically, this is related to increased replication stress and genome instability caused by aberrant expression of cell cycle regulators and DNA repair genes, independent of the classical RhoA effectors ROCK and DIAPH. These data establish *RHOA* E40Q as an unusual, context-dependent oncogenic driver: a seemingly inactive variant that unleashes its tumor-promoting potential only when the WT allele is absent.

## Abstract 2

A mutation in the RhoA gene promotes cancer if no normal RhoA is present.

## Introduction

Genomic analyses of cancers revealed constitutively activating mutations of oncogenes and inactivating mutations of tumor suppressors but also mutational hot spots with unclear effects on protein function. While some of these hot spot mutations might be passenger mutations, others might be of importance for cancer formation — for example, by modulating protein activity to a certain range or by enabling novel protein interactions. Identifying such noncanonical driver mutations and revealing the mechanism by which they promote tumor progression not only will conceptually advance our understanding of cancer but also conceivably open new avenues for cancer therapy.

An example of a gene with hot spot mutations of unknown function is *RHOA*. *RHOA* is a ubiquitously expressed small GTPase that regulates actin cytoskeletal organization, cell contraction, and other biological effects, using a range of different effector molecules, including the ROCK kinases and the DIAPH formins (1, 2). Because constitutively activated *RHOA* promotes cancer-relevant cellular effects such as proliferation and epithelial-mesenchymal transition (EMT) (3, 4), an oncogenic role was expected for *RHOA* (5, 6). Surprisingly, however, no canonical, constitutively active *RHOA* mutants have been found in cancer to date. Instead, several hot spots with noncanonical mutations were detected, often with a strong preference for a specific cancer type (6). A dominant-negative *RHOA* G17V mutation was reported to facilitate lymphoma formation (7–11), and the gain-of-function (GOF) mutation *RHOA* Y42C was identified as a driver mutation in gastric cancers (12). Most noncanonical mutations of *RHOA*, however, are not functionally characterized, making the role of RhoA in cancer still enigmatic.

One of these noncanonical mutations with unknown function is *RHOA* E40Q, which is found in head and neck squamous cell carcinoma (HNSCC) and, to a lesser extent, in breast cancer (13). HNSCC is the seventh most frequent cancer type worldwide and accounts for approximately 450,000 deaths every year (14–16). Because no effective screening test is available, most HNSCC cases are diagnosed at an advanced stage. Importantly, the poor survival rates of patients who are human papilloma virus (HPV) negative and have HNSCC have not changed significantly in recent decades, indicating a need for new targets for intervention and therapeutic agents (14). KO of *Rhoa* in keratinocytes in mice did not cause a developmental phenotype, but KO increased the susceptibility to skin tumor formation induced by 7,12-dimethylbenz[a]anthracene/12-*O*-tetradecanoyl phorbol-13-acetate (DMBA/TPA), supporting a role for *RHOA* signaling in HNSCC (17).

We report now that *RHOA* E40Q is a loss-of-function (LOF) mutation that displays its full cancer-promoting potential only in the absence of WT RhoA. Although keratinocyte-restricted ectopic expression of *RHOA* E40Q in a mouse model in the presence of WT *Rhoa* did not cause an obvious phenotype, expression of *RHOA* E40Q in keratinocytes lacking WT *Rhoa* resulted in absent fur formation and spontaneous formation of well-differentiated epidermal squamous cell carcinoma (SCC) within a few weeks. Cellular studies further revealed that *RHOA* E40Q is an LOF mutation that, in the absence of WT *RHOA*, correlates with increased migration, increased proliferation, and decreased genome maintenance.

These data support an HNSCC-promoting function of *RHOA* E40Q in the absence of WT RhoA and a role for RhoA signaling in genome maintenance, paving the way for innovative therapeutic approaches for the treatment of HNSCC with alterations in RhoA signaling.

## Results

### In HNSCC, RHOA E40Q is often associated with a heterozygous deletion of the RHOA gene.

The point mutation *RHOA* E40Q, which is in the Switch I domain mediating interaction of RhoA with effectors and activating guanine nucleotide exchange factors, is found in 4 of 496 HNSCC cases in The Cancer Gene Atlas (TCGA) PanCancer Atlas dataset. All *RHOA* E40Q HNSCC cases were HPV negative, like the majority of the HNSCC TCGA PanCancer dataset. *RHOA* E40Q was also detected in 0.2% of breast cancers but not in any of the 30 additional cancer types analyzed by TCGA, indicating a highly cancer type–specific occurrence. A heterozygous loss of *RHOA* corresponding with a decreased *RHOA* expression was noted in 72% of the HNSCC cases (Supplemental Figure 1, A and B; supplemental material available online with this article; https://doi.org/10.1172/JCI201975DS1). Similarly, 3 of the 4 *RHOA* E40Q HNSCC cases showed a heterozygous loss of *RHOA* and reduced expression of *RHOA*, suggesting that *RHOA* E40Q cancers, in most cases, are not expressing a WT version of *RHOA*. As with *RHOA* E40Q, the missense mutations *RHOA* T37S and *RHOA* G62E, are located in Switch regions and occur on a background of a heterozygous loss of *RHOA*. Interestingly, we observed a significant enrichment of the amplification of the *MYC* oncogene in this group of point mutations, using Welch’s *t* test for unequal variances and unequal sample sizes (Supplemental Figure 1C).

If *RHOA* E40Q mutations are of functional relevance in cancer, we speculated that other RhoA signaling genes also might be genetically altered in HNSCC. Therefore, we assessed the HNSCC dataset for copy number alterations of Rho subfamily GTPases (*RHOA*, *RHOB*, *RHOC*), *RHOA* effectors, and *RHOA* regulators (guanine nucleotide exchange factors, inactivating GTPase activating proteins, Rho GDP dissociation inhibitors [GDIs]) (Supplemental Table 1). *RHOA* was rarely deleted in HNSCC and never amplified. In contrast, *RHOB* was rarely amplified but never deleted, whereas *RHOC* was co-deleted together with *NRAS* in 2% of the HNSCC cases. With respect to *RHOA* effectors, the kinase *ROCK1* was amplified in 2% of the HNSCC cases, whereas *ROCK2* was not altered. On the other hand, the actin polymerization–promoting formin *DIAPH2* was deleted in 5% of the HNSCC cases. Of the Rho inhibiting GDIs, *GDI1*, which does not bind to RhoB and, therefore, only inhibits RhoA and RhoC, was amplified in 3% of the HNSCC cases. Finally, we noted that the HNSCC driver genes *MYC*, *PIK3CA*, and *FGF3* were co-amplified with certain RHO GTPase signaling genes, which might lead to alterations in RHO signaling (Supplemental Figure 1D).

Taken together, these data indicate *RHOA* E40Q is a hot spot mutation that, surprisingly, is associated with a heterozygous loss of the second allele, which is not typical of an oncogene.

### Establishment of RHOA KO SCC cell lines expressing RHOA E40Q.

To investigate RhoA E40Q function in keratinocyte-derived SCC cells lacking WT *RHOA* in vitro, 4 SCC cell lines were selected: SCC27 (tongue), which lacks detectable *RHOA* expression; CJM (oral cavity), which expresses both *RHOA* E40Q and *RHOA* WT; the fibroblastoid line H376 (oral cavity); and the epithelioid cell line SCC12 (skin SCC). To model the lack of WT *RHOA* observed in most SCCs expressing *RHOA* E40Q, we first abrogated expression of endogenous WT *RHOA* by introducing frameshift mutations in the coding region of *RHOA* by CRISPR/Cas9-induced DNA double-strand breaks (DSBs) in all lines except SCC27. KO of *RHOA* was confirmed by quantification of Western blots indicating a reduction of RhoA protein in the polyclonal cell mixtures by at least 70% in H376, SCC12, and CJM (*RHOA* KO) (Figure 1). In these cells, we then expressed HA-tagged *RHOA* E40Q (*RHOA* KO *+* *RHOA* E40Q; hereafter, E40Q) or HA-tagged *RHOA* WT (*RHOA* KO *+* *RHOA* WT) or empty vector (*RHOA* KO + empty). Importantly, the gRNA used for inactivation of the endogenous *RHOA* gene was located at an intron-exon border and, therefore, could not inactivate the transduced *RHOA* cDNA, which lacks intronic sequences. Because the HA-tagged *RHOA* contained a second start codon at the beginning of *RHOA*, we sometimes observed that translation started at this second ATG, resulting in a RhoA protein lacking the HA tag. In SCC12 and CJM cells, quantifications of ectopically expressed RhoA E40Q and RhoA WT proteins were not significantly different from each other or from the level of endogenously expressed RhoA in WT cells transduced with empty vector (Figure 1A). H376 cells had less RhoA E40Q expression compared with RhoA WT in(*RHOA* KO + *RHOA* WT and *RHOA* WT + empty, whereas SCC27 had similar ectopic expression of RhoA E40Q and RhoA WT.

### RHOA E40Q is a partial LOF mutation.

Loss of *RHOA* resulted in a significant increase of RhoB protein in H376, SCC12, and CJM cells (Figure 1A). This showed that residual WT cells in the polyclonal mixtures were not sufficient to mask this phenotype. Ectopic expression of RhoA WT rescued this phenotype. In RhoA-deficient SCC27 cells, expression of RhoA WT decreased RhoB protein. These data indicate RhoB protein levels in SCC cells are regulated by RhoA.

Expression of RhoA E40Q in RhoA-deficient SCC cell lines, however, reduced RhoB expression less efficiently than RhoA WT in H376, SCC12, and CJM, whereas no significant reduction of RhoB was observed in SCC27 cells expressing RhoA E40Q (Figure 1, A and B). Although the limited reduction in H376 cells might be related to a reduced expression of RhoA E40Q compared with RhoA WT, these data suggest RhoA E40Q is less functional than RhoA WT.

We then assessed different downstream pathways of RhoA signaling. Myosin light chain (MLC) phosphorylation is promoted by the Rho effector ROCK. Paradoxically, the absence of RhoA significantly increased the level of phosphorylated MLC (pMLC) in H376, SCC27, and SCC12, whereas it was not altered in CJM (Figure 1A). This effect was rescued by re-expression of RhoA WT in H376 and SCC27 cells and nonsignificantly in SCC12 cells. Expression of RhoA E40Q in RhoA-deficient cells, however, did not show a significant rescue effect in any of the cell lines.

RhoA can promote phosphorylation of cofilin via ROCK/LIMK and of Erk and Akt by less characterized pathways (16). However, no significant alteration of cofilin phosphorylation (pCof), Erk phosphorylation (pERK), or Akt phosphorylation (pAkt) was observed for RhoA KO or by expression of RhoA WT or RhoA E40Q in *RHOA* KO cells (Supplemental Figure 2A).

To distinguish whether RhoA E40Q is promoting pMLC by direct activation of ROCK or indirectly via RhoB, we deleted the *RHOB* gene in H376 or CJM *RHOA* KO cells expressing *RHOA* WT or *RHOA* E40Q cDNA. Efficient loss of RhoB was confirmed by Western blot (Figure 1C). In the absence of endogenous RhoA and RhoB, expression of RhoA E40Q did not induce a higher level of pMLC than ectopic expression of RhoA WT. Furthermore, no alteration of pCof, pAkt, or pERK was observed (Supplemental Figure 2B).

These data indicate that RhoA E40Q–expressing *RHOA* KO cells have elevated pMLC levels dependent on RhoB, as in *RHOA* KO, and suggest that RhoA E40Q is a partial-LOF mutation.

### RHOA T37S and G62E behave as LOF mutations in HNSCC.

In patients, HNSCC with a *RHOA* T37S or *RHOA* G62E mutation shows heterozygous loss of *RHOA* and amplification of *MYC*, like *RHOA* *E40Q* HNSCC (Supplemental Figure 1, A and C). To test whether *RHOA* T37S and *RHOA* G62E show an LOF phenotype, as in *RHOA* E40Q, we stably overexpressed these mutants in H376 cells lacking endogenous *RHOA*. T37S and, even more so, G62E had lower protein levels than RHOA WT, suggesting the possibility of an increased degradation of these mutants compared with WT (Supplemental Figure 3). *RHOA* T37S and *RHOA* G62E had higher RhoB protein levels than did *RHOA* WT–expressing cells. Moreover, they both had significantly increased pMLC expression, similar to *RHOA* E40Q. Additional deletion of *RHOB* completely abrogated this effect and, interestingly, resulted in increased protein levels of RhoA T37S and G62E. These data suggest *RHOA* T37S and *RHOA* G62E are LOF mutations similar to *RHOA* E40Q.

### RHOA E40Q does not strongly affect cell cycle or EMT in the SCC lines tested.

RhoA signaling affects cell cycle and EMT (3, 4). Investigating the cell cycle by analysis of DNA content (via DAPI staining) did not reveal any obvious change in *RHOA*-deficient or *RHOA* E40Q–expressing cells compared with *RHOA* WT–expressing cells in H376, SCC12, and CJM (Supplemental Figure 4A). Because SCC27 showed a minor alteration, we performed a cell cycle analysis by 5-ethynyl-2′-deoxyuridine (EdU) incorporation. This revealed a slight decrease in G2/M but also a doubling of re-replicating cells, though at a low level (Supplemental Figure 4B).

To assess a possible role of RhoA E40Q in EMT, we performed immunofluorescence microscopy. Expression and nuclear localization of YAP, an EMT-promoting transcription factor that is activated downstream of RhoA (17), were not obviously altered by loss of *RHOA* or re-expression of E40Q, either in the epithelioid SCC12 (Supplemental Figure 5) or in H376, SCC27, or CJM (data not shown). Furthermore, staining for F-actin and the epithelial markers E-cadherin and β-catenin did not indicate a strong alteration in *RHOA* KO + empty or E40Q in the SCC cell lines (Supplemental Figure 6 and data not shown). These results do not support a prominent role for RhoA or RhoA E40Q in EMT or the cell cycle in the SCC cell lines tested.

### RHOA E40Q expressing RHOA KO SCC cells have increased ROCK-dependent migration.

Rho signaling affects cell migration in keratinocytes in a ROCK-dependent manner (18). Therefore, we investigated whether deletion of the *RHOA* gene and expression of *RHOA* E40Q affect HNSCC migration in a 2D wound-closure assay in which proliferation was inhibited by serum-free medium. We found that loss of *RHOA* or expression of *RHOA* E40Q in the absence of WT RhoA significantly increased migration of H376, SCC27, and SCC12 (Figure 2). No effect was found in CJM cells, which correlated with their unaltered pMLC levels observed earlier.

Next, we assessed whether the increased migration is dependent on ROCK, by treating the migrating cells with the ROCK inhibitor (ROCKi) Y27632. ROCK inhibition, indeed, prevented the quicker migration of *RHOA* KO + empty or E40Q cells compared with *RHOA* WT–expressing cells (Figure 2, A and B).

These data confirm that *RHOA* *E40Q* is an LOF mutation that promotes cancer-relevant cell migration by a RhoB/ROCK/pMLC pathway, like *RHOA* KO.

### RhoA E40Q and RhoB are located both in cytoplasm and nucleus.

Recently, we demonstrated that RhoA signaling is regulated independently in cytoplasm and nucleus (19). To test whether ectopically expressed HA-tagged RhoA E40Q and Myc-tagged RhoB can enter the nucleus, we performed immunofluorescence staining and analyzed the results by confocal microscopy. Nuclei were identified by DAPI and cell area by fluorescently labelled phalloidin, via detection of F-actin. Quantitative image analysis suggested that both RhoA E40Q and RhoB are located in the cytoplasm and nucleus, and the nuclear fraction of cellular RhoB is about twice as high as that of RhoA E40Q (Supplemental Figure 7A).

### RHOA KO and RHOA E40Q reduced fitness of breast epithelial cells in a ROCK-independent manner.

The *RHOA* E40Q mutation is mainly found in HNSCC, but, to a lesser extent, it also is found in breast cancer. To test the functionality of *RHOA* E40Q in breast epithelial cells, we applied the CRISPR Select^TIME^ assay for functional testing of cancer gene variants of unknown significance to MCF10A cells (20, 21). CRISPR-Select^TIME^ is a competitive growth assay that monitors changes in variant frequencies over time to assess their effects on cell proliferation and survival. In this study, CRISPR-Select^TIME^ was performed to evaluate the relative growth dynamics of cells harboring the *RHOA* E40Q or *RHOA* K6*(KO) mutations compared with the WT allele carrying a silent mutation (WT′). The fraction of each mutation relative to WT′ was quantified at 2 days and 12 days after transfection of the gene-editing tools, allowing the detection of potential fitness advantages or disadvantages associated with the introduced variants. Importantly, the point mutations were introduced into the endogenous *RHOA* gene and, therefore, were expressed under the control of the endogenous *RHOA* promoter, avoiding problems related to unphysiological expression levels of the mutants. *RHOA* KO, and the *RHOA* E40Q mutation even more so, strongly reduced the fitness of MCF10A cells, as shown by a decreased amount of the mutants relative to *RHOA* WT′ after 10 days of culture (Supplemental Figure 7B). ROCK inhibition by Y27632 did not alter the reduced fitness of *RHOA* KO and *RHOA* E40Q relative to *RHOA* WT′, indicating that the reduction of cellular fitness is independent of ROCK signaling, in contrast to the regulation of cell migration.

These results prove the *RHOA* E40Q mutation is not neutral and that it lacks a growth-promoting effect in MCF10A cells. The reduced fitness is comparable to that induced by a pathogenic variant of *BRCA2*, a key molecule for DNA repair by homologous recombination (20). Therefore, it is possible that *RHOA* plays a role in genome maintenance in MCF10A cells.

### Mice with a keratinocyte-restricted expression of RHOA E40Q in the absence of WT Rhoa develop spontaneous SCC.

We reported previously that mice with a keratinocyte-restricted loss of RhoA show normal skin development but form malignant skin tumors in response to DMBA/TPA treatment (18). To reveal the effects of *RHOA* E40Q on the development of keratinocyte epithelium, we crossed mice with a keratinocyte-restricted KO of *Rhoa* (*Rhoa* locus) with mice that ectopically express *Rhoa* E40Q (ROSA26 locus) in a keratinocyte-restricted manner, obtaining mice expressing HA-tagged *RHOA* E40Q in the absence of E40Q mice expressing both *RHOA* E40Q and *RhoA* WT (*Rhoa* HET + *RHOA* E40Q; hereafter HET) or control mice that did not express RhoA E40Q and had no reduction of endogenous RhoA levels (Supplemental Figure 8). Surprisingly, E40Q mice had strongly impaired fur formation and were completely or mostly naked (Figure 3A). Although hair follicles formed, no normal hair shafts could be detected in naked skin of 8-week-old mice (Figure 3B, arrowheads). Altered hair shafts, but no inflammation, were observed already in skin of 9-day-old mice, indicating that the hair-loss phenotype is not related to an immune reaction (Supplemental Figure 9). Where patches of hair were visible, they were maintained normally for until least 3 months of age.

Body weight of male and female E40Q mice was slightly reduced compared with that of control mice (Figure 3C). Moreover, E40Q mice spontaneously developed well-differentiated SCC in the skin within a few weeks, particularly at the outer ear lobes and around the anus (Figure 3, A, D, and E, and Supplemental Figure 10). Tumor onset was significantly earlier in naked and in female mice, although all mutant mice had tumors at 13 weeks of age (Figure 3D and data not shown).

Invasiveness of the tumors was indicated by an irregular contour line and by groups of cells separated from the main tumor, as seen by H&E staining or by immunofluorescence staining for the keratinocyte marker keratin 5 (Figure 3E, white arrowheads). In large tumors, lymphocytic infiltrates were observed at the edges (Figure 3E and Supplemental Figure 10B, blue arrowheads) and draining lymph nodes were enlarged (data not shown). Cancers showed hyperkeratosis and the formation of keratin pearls, indicating the ability of cancer cells to terminally differentiate (Figure 3E and Supplemental Figure 10B, yellow arrowheads).

Importantly, HET mice, which homozygously express *RHOA* E40Q (ROSA26 locus) and heterozygously *Rhoa* WT (*Rhoa* locus), had normal fur and did not display any cancer within 15 weeks of age, confirming that *RHOA* E40Q is not a GOF mutation (GOF) (Figure 3, A and D).

Tumor-free epidermis in ears and back skin of E40Q mice was thickened compared with that of control mice (Figure 4, A and B), and no thickening was observed in HET mice (data not shown). At 9 days, epidermis was much thicker in ears than in back skin and in ears at 3 weeks or 8 weeks of age. This correlated with an increased frequency of Ki67^+^ proliferating cells in the ear epidermis of 8-week-old mice compared with control mice (Figure 4, C and D). It also correlated with the higher frequency of cancer observed in ears compared with back skin and the early occurrence of cancers in the ear.

These in vivo data indicate a strong cancer-promoting function of *RHOA* *E40Q* in the absence of WT *Rhoa* but not in its presence.

### RHOA E40Q impairs genome maintenance in the absence of RHOA WT.

The early onset of spontaneous cancers in E40Q mice as well as the results from the CRISPR Select^TIME^ assay let us speculate that defective genome maintenance could contribute to the cancer formation. Therefore, we assessed the formation of DNA DSBs by immunofluorescence staining of ear and back skin sections for γH2AX. Epidermis of E40Q mice showed a significant increase in the percentage of cells with nuclear foci of γH2AX compared with control mice (Figure 5), suggesting an increased frequency of DSBs and an increased genome instability. A high frequency of nuclear γH2AX foci was also observed in cancers of E40Q mice (Supplemental Figure 11).

To further investigate this phenotype, we isolated primary keratinocytes from E40Q, HET, and control mice. Primary keratinocytes of E40Q and HET mice had protein levels similar to that of HA-tagged RhoA E40Q cells, comparable to about 30% of the endogenous RhoA expression (Supplemental Figure 12). Expression of RhoB and pMLC was strongly increased in E40Q mice compared with controls, which is in line with our observations in SCC cell lines. Interestingly, HET keratinocytes showed an intermediate increase of RhoB but no alteration in pMLC, indicating that the increase of RhoB protein in the presence of RhoA is not affecting pMLC.

Cell cycle analysis indicated an increase in re-replication, a reduction in G0/G1, and an increase in S and G2/M phase in E40Q keratinocytes compared with the control (Figure 6A and Supplemental Figure 13A), which matches the increased keratinocyte proliferation observed in vivo. In the absence of irradiation, the frequency of DSBs identified as nuclear γH2AX foci (Supplemental Figure 13B) was significantly elevated in S phase and re-replicating cells (Figure 6, B and C). Following irradiation with 2 Gy, the frequency of γH2AX foci in S phase, G2/M phase, and re-replicating cells was significantly increased after 0.5 hour and 1 hour. No increase in γH2AX foci frequency was observed in keratinocytes derived from HET mice (Figure 6D).

Replication stress can cause the formation of micronuclei. Indeed, primary keratinocytes from E40Q mice had approximately twice the frequency of micronuclei than did HET or control keratinocytes (Figure 6, E and F). These data validate the hypothesis that genome maintenance is impaired in E40Q keratinocytes.

To assess whether RhoA E40Q also impairs genome maintenance in HNSCC cell lines, we tested the SCC27-derived cell lines for their response to irradiation. At 0.5 hours after irradiation with 2 Gy, SCC27 cells expressing *RHOA* *E40Q* displayed a significantly increased frequency of γH2AX foci compared with SCC27 cells, which lack *RHOA*, and compared with SCC27 cells expressing *RHOA* WT (Supplemental Figure 13, B–D). At other time points, no significant difference was detected.

These results indicate *RhoA* *E40Q* is negatively affecting genomic maintenance in the absence of WT RhoA, which may contribute to cancer development.

### RhoA E40Q regulates genome maintenance in the absence of RhoA WT independent of ROCK and DIAPH.

Recently, the RhoA effector mDia1 was shown to contribute to DNA repair, suggesting a possible involvement of RhoA in genome maintenance (22). In addition, the mDia2-encoding gene *DIAPH2* is frequently deleted in HNSCC. To obtain more knowledge of the mechanism underlying the genome maintenance defect induced by *RHOA* E40Q in the absence of *RHOA* WT, we tested the involvement of ROCK1 and ROCK2 and of the formins DIAPH1, 2, and 3 by inhibiting them with ROCKi or the formin inhibitor SMIFH2. If the defect is dependent on abnormal activation (e.g., too high, too low, wrong place) or ROCK or DIAPH, the inhibitor should abrogate the difference between E40Q and controls. The inhibitors would also reveal if ROCK or DIAPH is playing a role in genome maintenance of WT keratinocytes. To distinguish acute from more long-term effects, we applied the inhibitor treatment for 1 hour and for 24 hours.

Treatment of keratinocytes with ROCKi or SMIFH2 for 1 hour did not rescue cell cycle alterations; it increased γH2AX foci 0.5 hours after irradiation and increased phosphorylated replication protein A (pRPA) foci, a marker of replication stress, in the absence of irradiation in E40Q primary keratinocytes (Figure 7). Similarly, increasing the duration of inhibition to 24 hours did not rescue cell cycle changes; it increased γH2AX foci 0.5 hours after irradiation and increased micronuclei (Supplemental Figure 14). Inhibitor treatment had no effect on *Rhoa* HET + *RHOA* WT or control cells in any of the assays compared with DMSO treatment. These data suggest RhoA E40Q regulates genome maintenance in the absence of RhoA WT independent of ROCK and DIAPH.

### RhoA E40Q regulates genome maintenance in the absence of RhoA WT independent of RhoB.

E40Q keratinocytes had increased expression of RhoB, leading to increased phosphorylation of MLC (Supplemental Figure 12). Analysis of transcriptomic and proteomic data from 2 independent cohorts of patients with HNSCC (23, 24) (*n* = 218 tumors and 102 matched adjacent normal tissues) revealed that in normal tissue adjacent to HNSCC, *RHOA* mRNA negatively correlated with *RHOB* mRNA (Supplemental Figure 15A). In HNSCC, *RHOA* mRNA was significantly decreased, whereas RhoB protein was significantly increased (Supplemental Figure 15B). These data confirm a negative effect of *RHOA* signaling on *RHOB* expression.

Could increased RhoB signaling be involved in the impaired genome stability observed in E40Q keratinocytes? To assess this question, we knocked down RhoB by siRNA (Figure 8A). This did not affect cell cycle, replication stress, and γH2AX foci after irradiation of HET or control cells (Figure 8, B–E). However, it strongly reduced S phase and increased G0/G1 in E40Q keratinocytes, suggesting a redundant function of RhoA and RhoB in cell cycle control. Re-replication, however, was not altered. Furthermore, the increase neither in pRPA and micronuclei nor in irradiation-induced γH2AX foci was rescued by the knockdown of RhoB. Thus, the impaired genome maintenance of E40Q keratinocytes appears not to be related to a “toxic” function of RhoB.

### RhoA E40Q is defective in loading GTP.

To test whether the E40Q mutation affects the GTP-binding of RhoA, we performed pull-down experiments for GTP-bound RhoA with SCC27 cells and primary keratinocytes in the presence and absence of GTPgS, a nonhydrolyzable GTP analog. This assay showed a 2- to 3-fold increase of RhoA E40Q precipitate after preincubation with GTPgS, suggesting that RhoA E40Q can bind GTP. However, it also revealed a strongly reduced ability to load GTPgS compared with RhoA WT, supporting an LOF phenotype of RhoA E40Q (Supplemental Figure 16). In the absence of GTPgS, approximately 1%–2% of the input RhoA E40Q was pulled down, indicating a huge excess of GDP-bound RhoA E40Q. It should be noted that unspecific binding of GDP-bound RhoA E40Q to the beads could contribute to the pull-down. With respect to RhoB, the pull-down experiments suggested an increased fraction of GTP-bound RhoB protein in HET and E40Q compared with control cells. These results indicate that RhoA E40Q is defective in loading GTP.

### RhoA E40Q regulates genome maintenance in the absence of RhoA by controlling gene expression of cell cycle regulators and DNA repair genes.

To delineate the transcriptomic alterations associated with the *Rhoa* E40Q mutation, we performed RNA-seq in primary mouse keratinocyte cells of E40Q, HET, and control mice. Principal component analysis showed that control and HET samples clustered closely, whereas E40Q samples formed a distinct and separate cluster, indicating substantial transcriptional divergence induced by *RHOA* E40Q in the absence of RhoA WT (Supplemental Figure 17A). Consistently, the overall expression patterns of differentially expressed genes (DEGs) were highly similar between control and HET samples but markedly distinct from E40Q samples (Figure 9A). Comparing HET and E40Q, we identified 1,251 upregulated and 1,792 downregulated genes in the E40Q group (Figure 9B). Reactome enrichment analysis revealed that these DEGs were significantly enriched in several gene sets, with the top 2 being “activation of ATR in response to replication stress” and “HDR through homologous recombination (HRR)” (Figure 9C). Gene set enrichment analysis (GSEA) further demonstrated that pathways related to the “activation of ATR in response to replication stress” and “HDR through homologous recombination (HRR)” gene sets were significantly downregulated in E40Q cells (Figure 9D), indicating a decreased ability to respond to replication stress and DNA repair in E40Q keratinocytes, consistent with the results presented in the previous experiments. In contrast, gene signatures of eukaryotic translation initiation and the PI3K cascade downstream of FGFR3 were significantly upregulated in the E40Q group, suggesting a tendency toward enhanced cell proliferation and survival in the E40Q group (Figure 9D).

To further characterize the molecular network affected by the E40Q mutation, we performed protein-protein interaction analysis of DEGs and identified 10 distinct subnetwork clusters associated with diverse biological roles. The largest subnetwork was predominantly enriched for genes involved in cell proliferation and cell cycle regulation. Hub gene analysis identified the top 10 hub genes within this subnetwork, including genes related to checkpoints (*Cdc20*, *Bub1*, and *Bub1b*) and the mitotic process (*Ndc80*, *Nuf2*, and *Cenpe*), whose defects will cause chromosome instability, aneuploidy, and polyploidy (Supplemental Figure 17B).

Zooming in on the genes, we isolated all the significantly dysregulated genes in the top 2 gene sets. As shown in the heatmap (Figure 9E), 45 genes were significantly downregulated and 1 gene was significantly increased. Among them, we observed *Rad51*, whose decrease in expression can induce DNA re-replication, and the key DNA repair genes *Brca1* and *Brca2*, whose decrease can enhance tumor generation.

We further confirmed the association between *RHOA* and the indicated gene sets in transcriptomic data from a cohort of patients with HNSCC (*n* = 528 tumors) (25). As observed in cells, GSEA using *RHOA* expression as a continuous variable in the HNSCC transcriptomic data showed that *RHOA* expression was positively associated with the dysregulated gene sets observed in Figure 9C, including RhoA activity signatures (“Rho GTPase effectors” and “Rho GTPases activate formins”), “activation of ATR in response to replication stress,” and multiple gene sets related to DNA repair (HRR, SSA, and mismatch repair) (Figure 9F). Notably, the gene sets “formation of the cornified envelope” and “keratinization” were negatively associated with *RHOA* expression, which is consistent with the analysis of the primary E40Q keratinocytes (Supplemental Figure 17C).

The genes shown in Figure 9E also exhibited similar significant associations (*n* = 42 of 46) with RhoA expression in tumor samples (Supplemental Figure 17D). Notably, the key DNA mismatch repair gene *MLH1* strongly associated with *RHOA* expression (*R* = 0.65, *P* = 1.11 × 10^–63^) (Supplemental Figure 17E), indicating a relationship between RhoA and DNA repair. Therefore, we defined the genes within the ATR and DNA repair pathways that were altered in E40Q (Figure 9E) as the “E40Q-affected replication stress response (RSR) and DNA repair signature.” Hierarchical clustering based on this signature stratified patients into 2 distinct clusters, with GSEA confirming that the gene signature was significantly upregulated in patients in cluster 1 versus those in cluster 2 (Supplemental Figure 17F). Patients with high expression of the signature genes had significantly better survival outcomes, suggesting that E40Q-mediated alterations in replication stress response and DNA repair pathways are associated with patient prognosis (Figure 9G).

Thus, the transcriptomic analysis indicates the *RHOA* E40Q mutation in the absence of WT *RHOA* decreases the replication stress response and DNA repair, increasing genomic instability and thereby contributing to cancer progression.

## Discussion

*RHOA* E40Q has been identified as a mutation hot spot in HNSCC, but its biological activity as well as its importance for cancer formation were unclear. This study reveals that *RHOA* E40Q is an LOF mutation that promotes cancer development only in the absence of WT *RHOA*. This increased cancer formation is conceivably dependent on 3 cellular changes observed in keratinocytes: (a) increased proliferation, (b) decreased genome maintenance, and (c) increased migration. Interestingly, the increased proliferation might contribute to the decreased genome maintenance, because cell cycle dysregulation is known to contribute to replication stress (26). Replication stress, on the other hand, can result in DSBs and decreased genome stability (27). In skin, we observed spontaneous cancer formation, particularly in the ear lobe of very young E40Q mice, correlating with an increased thickness of the ear epidermis compared with back skin at 9 days of age. The frequency of DSBs detected by γH2AX staining, however, was similar in both skin regions, indicating that other factors such as a differential ability to perform error-free repair of DSBs by homologous recombination might contribute to the higher probability of SCC in the ear epidermis.

Earlier, a role of RhoA in EMT was reported (4), but neither immunofluorescence analysis of the SCC cell lines nor the transcriptomic analysis of the primary keratinocytes of the mutant mice gave any indication of EMT regulation by *RHOA* E40Q. In tumor-free skin sections of E40Q mice, no widespread inflammation was observed. Furthermore, the transcriptomic analysis of the primary E40Q keratinocytes did not reveal any pro-inflammatory signatures. Although inflammation is often a central driver in the initiation and progression of HNSCC (28), it does not seem to play a dominant role in the spontaneous cancer formation in E40Q mice.

Even more prominent than the tumor formation was the widespread loss of hairs in E40Q mice. Replication stress in stem cells is known to trigger senescence and ageing (27); therefore, it is possible that the defective hair formation is related to replication stress in hair follicle stem cells. Moreover, mice with defects in DNA repair by homologous recombination displayed a hair loss phenotype (29, 30). Indeed, we observed cancer formation particularly in mice with complete hair loss. Furthermore, hair that was not lost during the first 2 weeks of life was maintained normally in E40Q mice, pointing to a critical period early in life of the animals for both hair follicle destruction as well as cancer formation.

Finally, we observed a severe reduction of S phase after knockdown of *RHOB* in primary E40Q keratinocytes, suggesting a redundant function of *RHOA* and *RHOB* in cell cycle control in these cells.

Which molecular mechanisms underlie these phenotypes? RhoA is a master regulator of the actin cytoskeleton and regulates, via ROCK and the DIAPH formins, the formation and turnover of F-actin as well as cell contraction (1, 2). Impaired regulation of the actin cytoskeleton, however, appears not to be crucial for the altered cell cycle regulation or the defective genome maintenance observed in primary E40Q keratinocytes, because neither inhibitors of ROCK nor of formins rescued the corresponding cell cycle changes. Also, with normal keratinocytes, the inhibitor did not appear to have an effect on cell cycle control, corroborating that the respective RhoA effectors are not crucial players. We also excluded the possibility that a high, toxic amount of RhoB protein is the reason for the cell cycle changes. Instead, several gene groups associated with cell cycle regulation were differentially expressed in the transcriptome of E40Q keratinocytes, suggesting that altered regulation of gene expression might be a central factor explaining the cell cycle phenotype. Similar correlations were observed for the genome instability effect: no involvement of ROCK, formins, or RhoB, but differential expression of genes related to replication stress and DNA repair.

In contrast, the pro-migratory phenotypes of E40Q SCC cells were entirely dependent on a RhoB/ROCK/pMLC pathway, as proven by inhibitors and CRISPR/Cas9-mediated KO of *RHOB*. The same pathway was increased in primary E40Q keratinocytes. Although we did not formally show that this results in increased migration of the cells, this is extremely likely, because we earlier demonstrated a RhoB/ROCK/pMLC/migration pathway in *RHOA* KO keratinocytes (18).

Expression of *RHOA* E40Q in the absence of WT *RHOA*, therefore, results in an altered Rho signaling landscape in which Rho signaling is partially increased due to overcompensation by RhoB (ROCK, migration) and partially decreased (cell proliferation, genome maintenance) when RhoB is not able to compensate for the loss of *RHOA*, at least in the presence of RhoA E40Q.

Is RhoA-dependent control of cell cycle regulation and genome maintenance genes of relevance for the majority of HNSCCs that do not harbor the *RHOA* E40Q mutation? Our bioinformatic analysis of HNSCC transcriptomes suggested this is the case. Firstly, *RHOA* expression was strongly decreased in the majority of HNSCC. This decrease correlated with an increase of RhoB protein, indicating biological relevance of the change. Secondly, the expression of *RHOA* in HNSCC correlated with the expression of cell cycle regulators and DNA repair genes. Finally, low expression of replication stress and DNA repair genes, which had decreased expression in E40Q keratinocytes, strongly correlated with decreased overall survival of patients with HNSCC, indicating their disease importance.

Although we initially expected that *RHOA* E40Q is a GOF mutation, our data now suggest it is, rather, an LOF mutation. The absence of a phenotype in HET mice and a rather normal transcriptome of primary keratinocytes from these mice, which express WT *RHOA* together with *RHOA* E40Q, argue against a GOF of *RHOA* E40Q. An LOF of *RHOA* E40Q is further supported by the reduced ability to load GTP, the insufficient ability to repress *RHOB*, the similar effects of *RHOA* E40Q and *RHOA* KO point mutations in CRISPR Select^TIME^ cell fitness assays, and the similar gene expression changes observed in E40Q keratinocytes and in HNSCC with low levels of WT *RHOA* mRNA, suggesting a reduced RhoA function in both cases. Of note, even mice with a keratinocyte-restricted deletion of *Rhoa* alone showed a tumor-prone phenotype by increased formation of malignant cancer in response to DMBA/TPA treatment (18). In this model, loss of *Rhoa* resulted in increased proliferation and migration of keratinocytes; however, replication stress and DNA repair were not investigated. The more severe phenotype in E40Q mice could be explained if one assumes that *RhoA* E40Q prevents rescue of Rho signaling by competition with RhoB and RhoC restricted to the genome stability pathway.

Is the cancer-promoting role of *RHOA* E40Q on a *RHOA* KO background unique to *RHOA* E40Q, or could there be other *RHOA* mutants with a similar effect? In HNSCC, T37S and G62E are candidates for such driver mutations with a strong context dependency. As we present here, both are LOF mutations and they occur in HNSCC together with a heterozygous loss of *RHOA*, like *RHOA* E40Q. In bladder cancer, *RHOA* E47K is mostly associated with a heterozygous loss of *RHOA* (TCGA PanCancer dataset). If *RHOA* E47K is an LOF, it might promote cancer formation by similar pathways as *RHOA* E40Q. In line with this speculation, bladder cancer shows copy number alterations of RhoA signaling genes like those observed in HNSCC, with preferential amplification of *RHOB*, *ROCK1*, and *GDI1*, and preferential deletion of *RHOA* and *DIAPH2*.

In conclusion, *RHOA* E40Q presents the unexpected feature of an LOF point mutation that promotes cancer more than a complete loss of RhoA, but only in the absence of WT RhoA. Conceptually, we describe here a different type of driver gene. Classical oncogenes or tumor suppressors directly regulate cancer-relevant pathways. In contrast, a highly context-dependent driver gene mutation such as *RHOA* E40Q has an effect only or mostly in the presence of a suitable genetic background. Moreover, demonstrating that Rho signaling affects genome maintenance, in addition to invasion and proliferation, opens treatment options by taking advantage of the synthetic lethality concept (31). Future research should explore the details of the Rho signaling network controlling the expression of cell cycle regulators and DNA repair genes in HNSCC and translate the findings into therapies for HNSCC.

## Methods

### Sex as a biological variable.

Both male and female mice were included in this study, and primary murine keratinocytes were isolated from mice of both sexes.

### Cell lines.

H376, SCC27 cells (obtained from K. Jensen, Novo Nordisk Foundation Center for Stem Cell Medicine) and HEK293T cells (ATCC, CRL-3216) were grown in DMEM (Thermo Fisher Scientific, 31966021) with 10% vol/vol FBS (Cytiva, SV30160.03) and 100 U/mL penicillin/streptomycin (P/S) (Thermo Fisher Scientific, 15140122). SCC12 cells were grown in DMEM/F12, HEPES (Thermo Fisher Scientific, 31330038) containing 10% vol/vol FBS, and 100 units/mL P/S. CJM cells were obtained from RIKEN (RCB1034) and maintained in Ham’s F10 Nutrient Mix (Thermo Fisher Scientific, 11550043) containing 10% vol/vol FBS and 1% vol/vol P/S (100 units/mL). iCas9 MCF10A cells with Cas9 expressed from a stably integrated TRE3G Edit-R inducible Lentiviral Cas9 construct (Horizon, CAS11229) were cultured in DMEM/F12, HEPES supplemented with 5% (vol/vol) horse serum (Thermo Fisher Scientific, 26050088), 10 μg/mL insulin (Sigma, I1882), 20 ng/mL EGF (Peprotech, AF-100-15), 0.5 μg/mL hydrocortisone (Sigma, H0888), and 100 ng/mL cholera toxin (Sigma, C8052). All cells were cultured in a humidified 37°C incubator with 5% CO_2_.

### Generation of KO cell lines.

KO cell lines for H376, SCC12, SCC27, and CJM cells were generated by CRISPR/Cas9 genome editing. sgRNAs targeting the coding region were designed using crispor.tefor.net and selected for the low frequency of predicted off-target effects and a location close to the transcription start site. The sgRNA for RhoA [RhoA_E2_1: 5′ tttcccattacagGTAGAGT(TGG) 3′ (+)] was cloned into the puromycin resistance–containing lentiCRISPRv2 vector (32). To generate RhoA and RhoB double KO cells, an sgRNA targeting RhoB but not RhoA [RhoB_1.3: 5′ AGGAGGACTACGACCGCCTG(CGG) 3′ (+)] was cloned into the LentiCas9-Blast vector (Addgene, 52962). Lentiviral particles were produced by transfecting HEK293T cells with pVSVG (Addgene, 8454), pPAX-2 (Addgene, 12260), and polyethylenimine (Sigma-Aldrich, P3143) in Opti-MEM reduced serum medium (Thermo Fisher Scientific, 31985070). Viral supernatants were harvested, filtered (0.45 μm), and used to transduce H376, CJM, SCC12, SCC27, and MCF10A. Puromycin or blasticidin selection began 48–72 hours after transduction (puromycin: H376, HaCaT, CJM, MCF10A: 2.5 μg/mL; SCC12: 1.5 μg/mL; SCC27: 3 μg/mL; blasticidin: H376: 5 μg/mL; CJM: 4 μg/mL) and continued until all control cells were eliminated. KO efficiency of polyclonal cells was validated by Western blot.

### Generation of overexpression cell lines.

Mutations for E40Q, T37S, and G62E were introduced into HA-tagged human RhoA cDNA in pcDNA3.1(+) (19) via PCR, using phosphorylated full-match and mismatch primers amplifying the whole plasmid. The linear product was purified and ligated. WT and mutant RhoA cDNAs were amplified using the Phusion High-Fidelity PCR Kit (Thermo Fisher Scientific, F553L) and introduced into a lentiviral expression vector co-expressing GFP (Addgene, 12252). Viral particles were produced as described in the preceding paragraph. After expansion, transduced cells were sorted twice via flow cytometry for GFP^+^ cells, using the BD FACSMelody (BD Biosciences) system. RhoA expression was determined by Western blotting.

Using PCR, a Myc-tagged human RhoB was prepared and cloned into pcDNA3.1(+). The expression vector was transiently transfected to H376 WT, RhoA KO, and RhoA KO/E40Q cells using Lipofectamine LTX (Thermo Fisher Scientific, A12621) following the instructions of the manufacturer. Cells were seeded for staining 1 day after transfection and stained on day 2.

### Western blotting.

Cells at 70%–80% confluence were washed with ice-cold PBS and lysed in RIPA buffer (Thermo Fisher Scientific, 89900) supplemented with PhosphoSTOP (Roche, 04906845001), protease inhibitor cocktail (Roche, 11873580001), 1 mM PMSF (Merck, 10837091001), and 1 U/mL Benzonase nuclease (Merck, 70746-4). Cells were lysed with 100 μL of RIPA-based extraction buffer per well of a 6-well plate, collected by scraping, then incubated for 30 minutes on ice, and centrifuged at 14,000*g* for 15 minutes at 4°C. Protein concentration of the supernatant was determined using the BCA Protein Assay Kit (Thermo Fisher Scientific, 23227). Samples were mixed with NuPAGE Sample Reducing Agent (Thermo Fisher Scientific, NP0009) and NuPAGE LDS Sample Buffer (Thermo Fisher Scientific, NP0007), denatured at 95°C for 5 minutes, cooled on ice, and loaded onto 12.5% Bis-Tris gels. Electrophoresis was performed at 20 mA/gel, followed by semi-dry transfer to ethanol-activated PVDF membranes. Transfer quality was verified by Ponceau S staining (Thermo Fisher Scientific, A40000279). Membranes were blocked in 5% skim milk powder (Merck Millipore, 999999-99-4) in TBST for 1 hour at room temperature (RT), incubated overnight at 4°C with primary antibodies diluted in 5% BSA/TBST, and incubated for 1 hour at RT with HRP-conjugated secondary antibodies diluted in 5% skim milk and TBST. The following antibodies were used: RhoA (Cell Signaling Technology, 2117; 1:1,000), RhoB (Cell Signaling Technology, 63876; 1:1,000), pMLC (Cell Signaling Technology, 3674; 1:1000), pCof (Cell Signaling Technology, 3311; 1:1,000), pERK (Cell Signaling Technology, 9101; 1:1,000), pAKT (Cell Signaling Technology, 9271; 1:1,000), HA-Tag (Cell Signaling Technology, 3724; 1:1,000), and GAPDH (Sigma-Aldrich, G9545; 1:10,000). As secondary antibodies, HRP-coupled horse anti-mouse IgG (Vector Laboratories, PI-2000) or goat anti-rabbit IgG (Vector Laboratories, PI-1000) were used (1:1,000). Detection was performed by chemiluminescence using HRP substrate (Millipore, WBLUF0500). Proteins were then visualized with the ChemiDoc MP Imaging System (Bio-Rad). Each membrane had its own GAPDH loading control. Representative loading controls are shown in the figures. Quantification was performed using Fiji and normalized to GAPDH.

### Wound-closure assay.

Cell migration was evaluated using a scratch wound assay in a 96-well plate format. Cells were seeded in duplicate in complete growth medium at densities optimized to reach 100% confluency the following day, ensuring a well-defined wound area. The following seeding densities were used: SCC27, SCC12: 50,000 cells/well; H376: 60,000 cells/well; and CJM: 100,000 cells/well. After overnight incubation to allow cell attachment and monolayer formation, the medium was replaced with 100 μL of serum-free medium (DMEM or F10, depending on the cell line) to reduce proliferation during the assay. To prevent medium evaporation, 100 μL of PBS was added to the unused peripheral wells. Scratches were made using an Incucyte 96-well Woundmaker Tool (Sartorius) to create uniform and symmetrical wounds. After scratching, wells were gently washed once with PBS to remove detached cells, and 130 μL of fresh serum-free medium was added. Either DMSO (control) or 10 μM ROCKi (Y-27632 dihydrochloride; Tocris Bioscience, 1254) was applied immediately after scratching. Wound closure was then monitored using the IncuCyte S3 Live-Cell Analysis System (Sartorius) for 24 hours.

### Mice.

Mice with a conditional expression of HA-tagged *RHOA* E40Q were generated by introducing the corresponding cDNA together with an EF1α promoter and a Lox-Stop-Lox cassette into the ROSA26 locus by CRISPR/Cas9-mediated genome editing (Supplemental Figure 8). These mice were crossed with mice with a keratinocyte-restricted inactivation of the endogenous *Rhoa* gene (*Rhoa*^fl/fl^-K5-Cre (33) to obtain (a) mice with a keratinocyte-restricted loss of *Rhoa* and expression of *RHOA* E40Q (*Rhoa*^fl/fl^, E40Q/E40Q, K5-Cre; short: E40Q); (b) mice that express both *RHOA* E40Q and WT *Rhoa* (*Rhoa*^fl/+^, E40Q/E40Q, K5-Cre; short: HET); and (c) control mice (*Rhoa*^fl/fl^ E40Q/E40Q), which do not express *RHOA* E40Q and only express WT *Rhoa*. Animals were generated by the Transgenic Mouse Core Facility of the University of Copenhagen and were kept according to national and European animal welfare laws in an Association for Assessment and Accreditation of Laboratory Animal Care–accredited animal house under specific pathogen–free conditions.

### Histological analysis.

Paraffin sections of mouse skin fixed in 4% paraformaldehyde (PFA) in PBS were stained with H&E according to standard protocols. Epidermal thickness was measured on H&E-stained sections (using Fiji; ImageJ, NIH) as the distance from the basement membrane to the upper boundary of the viable epidermis, excluding the stratum corneum. Measurements were performed in interfollicular epidermis, avoiding hair follicles. Measurements were obtained in at least 3 locations per section and averaged to generate a single value per mouse.

### Immunofluorescence staining of mouse skin sections.

After euthanasia, ears, back skin, perianal skin, or tumor tissues were embedded in OCT mounting medium (VWR, 361603E), snap-frozen, and stored at –80°C. Frozen sections (8 μm) were prepared using a cryostat and mounted onto glass slides. Sections were fixed in 4% PFA for 10 minutes at RT, washed with PBS, permeabilized with 0.3% Triton X-100 in PBS for 10 minutes, and blocked with 5% BSA in PBS for 1 hour at RT. Primary antibodies were diluted in blocking buffer and incubated overnight at 4°C in a humidified chamber. After washes with PBS, secondary antibodies were incubated for 1 hour at RT in the dark. Nuclei were counterstained with DAPI (1:1,000 in PBS). Samples were mounted in fluorescence mounting medium (DAKO, Agilent Technologies, S3023).

The following primary antibodies were used: rabbit anti-γH2AX (Cell Signaling Technology, 2577; 1:500), rat anti-Ki67 (eBioscience, 14-5698-82; 1:100), and rabbit anti-cytokeratin 5 (Thermo Fisher Scientific, MA5-16372; 1:30). The following secondary antibodies were used: Alexa Fluor 568 goat anti-rabbit (Invitrogen, A-11011; 1:500), Cy5 goat anti-rat IgG (Invitrogen, A10525; 1:500), and Alexa Fluor 647 goat anti-rabbit (Invitrogen, A21245; 1:500).

Images were acquired using a ZEISS laser-scanning confocal microscope with Airyscan super-resolution and ZEN Blue software using a ×25 water-immersion objective and identical acquisition settings. To ensure comparability between samples, identical acquisition settings were used for all experiments. Images were quantified in QuPath (version 0.4.3). Nuclear segmentation was performed using the StarDist deep-learning-based algorithm to enable accurate recognition of nuclei. After segmentation, a Random Forest classifier was trained and applied to identify Ki67-positive and γH2AX-positive cells.

### Primary murine keratinocyte isolation and culture.

Primary murine keratinocytes were isolated from the back skin of 3-week-old mice, as previously described (34) but with the following modifications: Epidermal sheets were digested with 0.8% trypsin for 40 minutes and cells were resuspended in full keratinocyte medium supplemented with 0.2 mM CaCl_2_ and 2.5 μM ROCKi to promote cell attachment. The medium was replaced the following day with standard full keratinocyte medium.

### Inhibitor treatment and ionizing radiation.

For inhibitor experiments, primary murine keratinocytes were treated with either ROCKi or the formin inhibitor SMIFH2 (MedChemExpress, HY-16931) at a final concentration of 10 μM for 1 hour or 24 hours. For ionizing radiation experiments, cells were irradiated, 1 day after seeding, with 2 Gy using a Faxitron CP-160 X-ray apparatus (Faxitron Bioptics).

### Immunofluorescence staining of primary murine keratinocytes.

Primary murine keratinocytes were isolated as described under *Primary murine keratinocyte isolation and culture* and cultured for 2 days before being reseeded onto round glass coverslips (VWR, 631-1578) in 6-well plates. Immunofluorescence experiments were performed 24 hours after reseeding. After the treatment, the medium was quickly removed and the cells were incubated in pre-extraction buffer (25 mM HEPES, pH 7.5, 50 mM NaCl, 1 mM EDTA, 3 mM MgCl_2_, 300 mM sucrose, and 0.5% Triton X-100) on ice for 3 minutes and then fixed in 4% PFA for 15 minutes at RT, followed by permeabilization with 0.5% Triton X-100 for 20 minutes, and blocking in 2% BSA containing 0.1% Triton X-100 in PBS for 30 minutes at RT. Primary antibodies diluted in blocking buffer were incubated overnight at 4°C, followed by a 1-hour incubation with secondary antibodies at RT in the dark. Nuclei were stained with DAPI (1:1,000 in PBS). Samples were mounted in fluorescence mounting medium (DAKO; Agilent Technologies, S3023) and stored at 4°C.

The following primary antibodies were used: rabbit anti-γH2AX (Cell Signaling Technology, 2577; 1:500), mouse anti-γH2AX (Cell Signaling Technology, 80312S; 1:500), and rabbit anti-phospho-RPA32 (Ser33) (Fortis Life Sciences, A300-246A; 1:500). As secondary antibodies we used Alexa Fluor 488 goat anti-mouse (Invitrogen, A11029; 1:1,000), Alexa Fluor 488 goat anti-rabbit (Invitrogen, A11034; 1:1,000), and Alexa Fluor 568 goat anti-rabbit (Invitrogen, 11011; 1:1,000). Imaging was performed on the Olympus ScanR High-content screening station (×20). Quantification was performed using Olympus ScanR Analysis 2.8.1.

### siRNA transfection.

Primary keratinocytes were seeded in 6-well plates and transfected with a SMARTpool siRNA targeting mouse *Rhob* (Dharmacon/Horizon Discovery, L-064118-00-0005) or a nontargeting control siRNA (Dharmacon/Horizon Discovery, D-001810-10-05) using Lipofectamine RNAiMAX (Thermo Fisher Scientific, 13778075) according to the manufacturer’s instructions. After 24 hours, cells were reseeded onto round glass coverslips (VWR, 631-1578) and cultured overnight before immunofluorescence staining or Western blotting to verify *Rhob* knockdown efficiency.

### Cell cycle analysis.

Cell cycle analysis was performed by quantitative image analysis of DAPI-stained nuclei based on DNA content alone or by using the Click-iT EdU Cell Proliferation Kit for Imaging with Alexa Fluor 647 (Invitrogen, C10340) according to the manufacturer’s instructions. Images were acquired using an Olympus ScanR High-Content Screening Station equipped with a ×20 objective and analyzed using Olympus ScanR Analysis 2.8.1 software.

### RNA-seq.

Total RNA was extracted from primary murine keratinocytes using the RNeasy Kit (Qiagen) according to the manufacturer’s instructions. RNA integrity was assessed with an Agilent 2100 Bioanalyzer (Agilent Technologies), and only samples with RNA integrity number >9.5 were used for library preparation. RNA samples were fragmented, and first-strand cDNA was synthesized using random hexamer primers, followed by the second-strand cDNA synthesis. Libraries were prepared through end repair, A-tailing, adapter ligation, size selection, amplification, and purification. Library quality was evaluated using fluorometric quantification, real-time PCR, and Bioanalyzer analysis for fragment-size distribution. Following quality control, libraries were pooled based on effective concentration and target-sequencing depth and sequenced on an Illumina NovaSeq X Plus platform using 150 bp paired-end reads (PE150).

Raw sequencing reads were processed using fastp (35) to remove adaptor sequences, poly-N reads, and low-quality reads, generating high-quality clean reads. Clean reads were aligned to the *Mus musculus* reference genome (mm39) using HISAT2 (version 2.2.1) (36) with default parameters. Gene-level read counts were quantified using featureCounts (version 2.1.1) (37), and gene expression levels were normalized as fragments per kilobase of transcript per million mapped reads.

DEG analysis was performed using DESeq2 (version 1.42.0) (38). *P* values were adjusted using the Benjamini-Hochberg method to control the FDR. Genes with a *P* value ≤0.05 and |log2 fold change| ≥1 were considered differentially expressed. Functional enrichment analyses were conducted using clusterProfiler to identify significantly enriched Reactome pathways (adjusted *P* ≤ 0.05).

### GSEA.

GSEA was performed using GSEA software (version 4.3.3; Broad Institute) on ranked bulk transcriptomic profiles from primary keratinocytes and cohorts of patients with HNSCC (39). For comparisons between *RhoA* E40Q heterozygous and homozygous keratinocytes, genes were ranked using the Signal2Noise metric between conditions. For continuous analyses using *RHOA* expression as a continuous variable in patient transcriptomic datasets, genes were ranked based on Spearman’s correlation coefficients. The enrichment scores were calculated with 1,000 phenotype permutations. Gene sets were obtained from the Reactome v2026 database, and pathways with FDR *q* value <0.05 were considered significant.

### Patient omics datasets.

Publicly available HNSCC omics datasets were obtained for this study, including transcriptomic data from the HNSCC-TCGA_GDC_2025 cohort (39) (*n* = 528 tumor samples), as well as transcriptomic and proteomic data from the Clinical Proteomic Tumor Analysis Consortium (CPTAC) HNSCC cohort (23) (CPTAC-pan; *n* = 108 tumors and 46 matched adjacent normal tissues) and the HNSCC cohort 2 (*n* = 110 tumors and 63 matched adjacent normal tissues) (24).

### Gene signature–based patient stratification and survival analysis.

Data on patients in the HNSCC-TCGA_GDC_2025 cohort (25) were stratified, using hierarchical clustering analysis, based on gene expression signatures. Survival outcomes between groups were evaluated using Kaplan-Meier survival analysis, and statistical significance was determined by the log-rank test. Heatmaps and survival plots were generated using the pheatmap and ggplot2 packages in R.

Additional methods are provided in the Supplemental Methods.

### Statistics.

Data were evaluated using GraphPad Prism 10 software and presented as mean ± SD of at least 3 independent experiments. Statistical significance was determined either by the 2-tailed Student *t* test for data with only 2 groups, Welch’s *t* test, or by 1-way ANOVA with Tukey’s post hoc test for experiments with multiple groups. Transcriptomic gene associations were analyzed using Spearman’s correlation. Significant differences are indicated in the figures or their legends.

### Study approval.

The animal studies were approved by the Animal Experiments Inspectorate (Dyreforsøgstilsynet), Glostrup, Denmark (approval 2022-15-0202-00167).

### Data availability.

The RNA-seq raw and processed data have been deposited in the Gene Expression Omnibus under accession number GSE331033. This article does not report original code. Any additional information required to reanalyze the data reported in this article or materials are available from the lead contact upon request. Further information can be found online. Values underlying graphed data in main and supplemental figures are shown in the Supporting Data Values file. Uncropped blot images are provided as a supplemental file.

## Author contributions

JN, QW, XH, and CB conceptualized the study. SW, CM, XH, JBA, and CSS provided methodology. JN, QW, IC, EPK, LPN, MFP, SH, and THLP performed experiments. YL and JBA performed the meta-analysis. JN, QW, YL, and CB wrote the manuscript. The co–first authors JN and QW contributed equally and are listed in alphabetical order. JN initiated the project and contributed to the scientific direction of the study.

## Conflict of interest

The authors have declared that no conflict of interest exists.

## Funding support

- Novo Nordisk Foundation (grant NNF20OC0061734 to CB).
- European Union’s Horizon 2020 research and innovation program under the Marie Skłodowska-Curie grant agreement (no. 101034291 to QW).

## Supplementary Material

Supplemental data

Unedited blot and gel images

Supporting data values

## Figures and Tables

**Figure 1:**
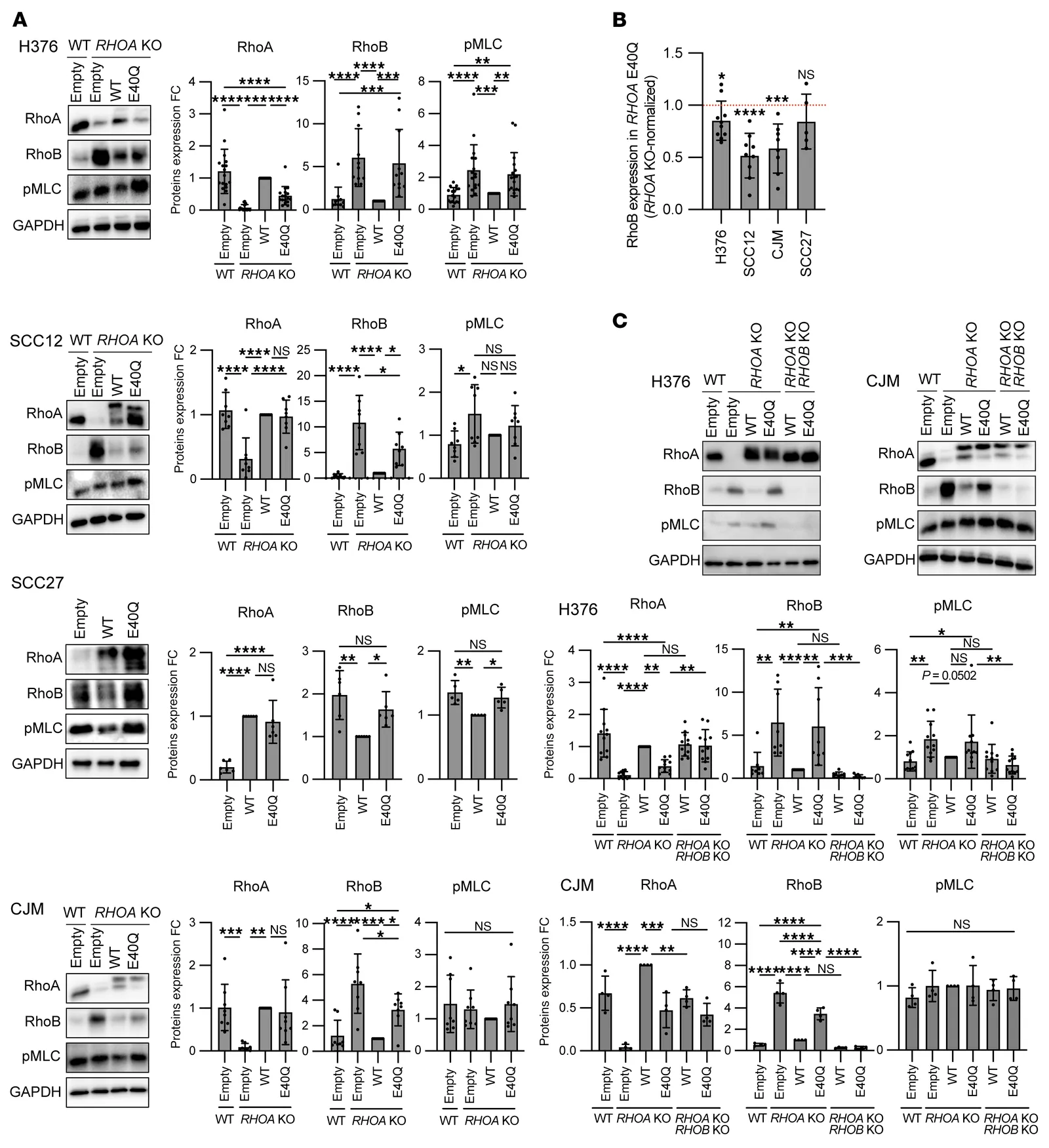
*RHOA* E40Q has similar signaling effects as *RHOA* KO. Quantification and representative examples of Western blots for RhoA, RhoB, pMLC, and GAPDH (loading control) with indicated cell types. Shown are WT cells transduced with empty vector (empty), or *RHOA* KO transduced with empty vector, *RHOA* WT (WT), or E40Q. Data are shown as fold change relative to *RHOA* KO + *RHOA* WT. Error bars represent SD of the mean. (**A**) H376 (*n* = 13–17), SCC12 (*n* = 8), SCC27 (*n* = 6), and CJM (*n* = 8). Statistical significance was determined using 1-way ANOVA. (**B**) Quantification of RhoB expression in *RHOA* KO cells transduced with *RHOA* E40Q relative to RhoB expression in RhoA KO cells. Statistical significance was assessed using an unpaired *t* test. (**C**) As additional cell types, *RHOA* KO + *RHOA* WT and E40Q with a *RHOB* KO were tested. H376 (*n* = 8–11), CJM (*n* = 4). The data for WT and *RHOA* KO for these figures are also included in **A**. Statistical significance was determined using 1-way ANOVA. *P < 0.05, **P < 0.01, ***P < 0.001, ****P < 0.0001.

**Figure 2:**
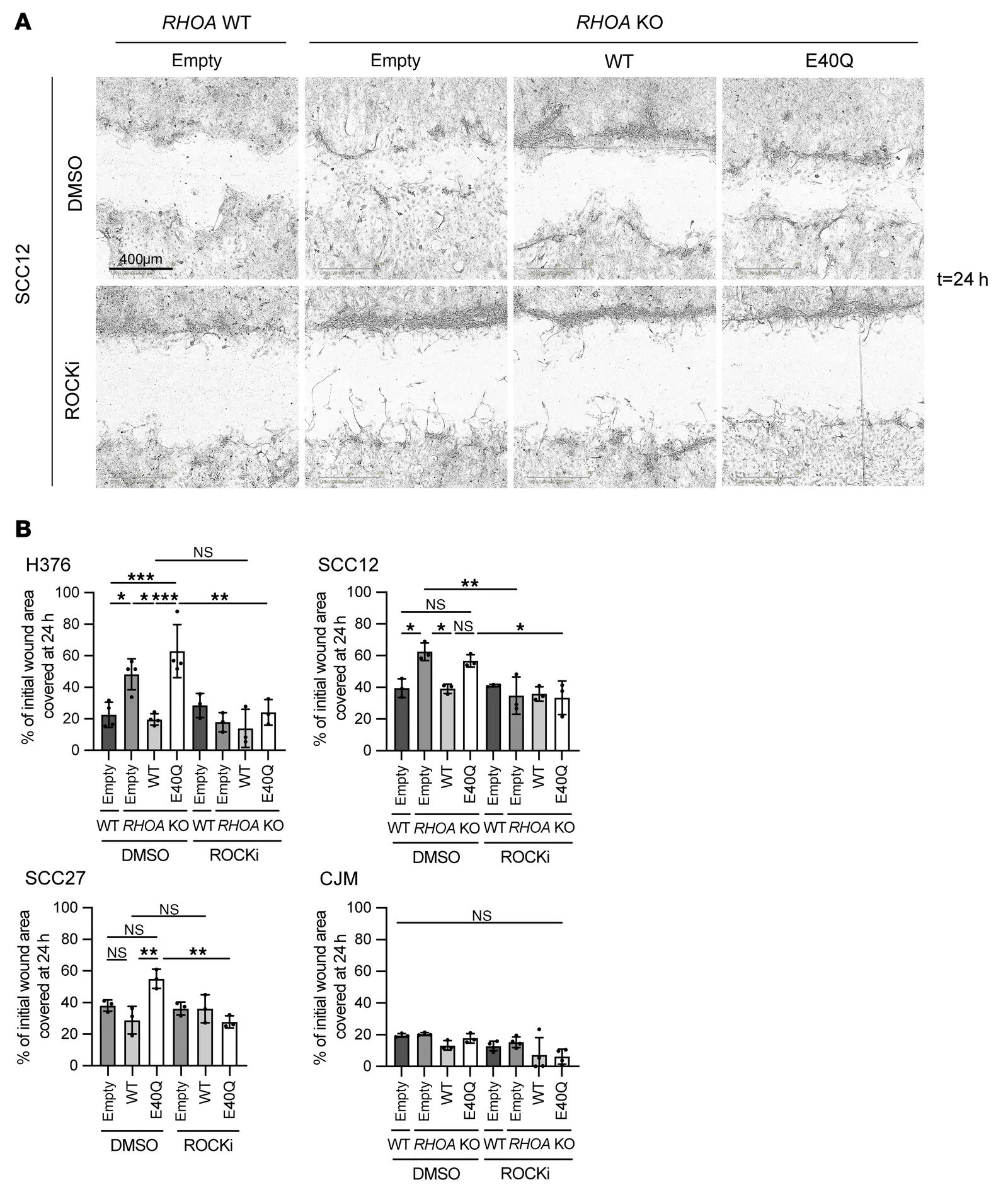
RhoA E40Q does not rescue increased ROCK-dependent migration of *RHOA* KO SCC cell lines. Scratch wound assays were induced in the indicated cell types in the presence of 10 mM ROCKi or DMSO as control. (**A**) Representative images of SCC12 cells showing wound closure after 24 hours, with or without ROCKi. (**B**) Migration quantified by the percentage of the initial wound area covered by cells after 24 hours. Error bars represent SD of the mean. Statistical significance was determined using 1-way ANOVA. Cells were seeded in duplicate; *n* = 3–4. *P < 0.05, **P < 0.01, ***P < 0.001.

**Figure 3:**
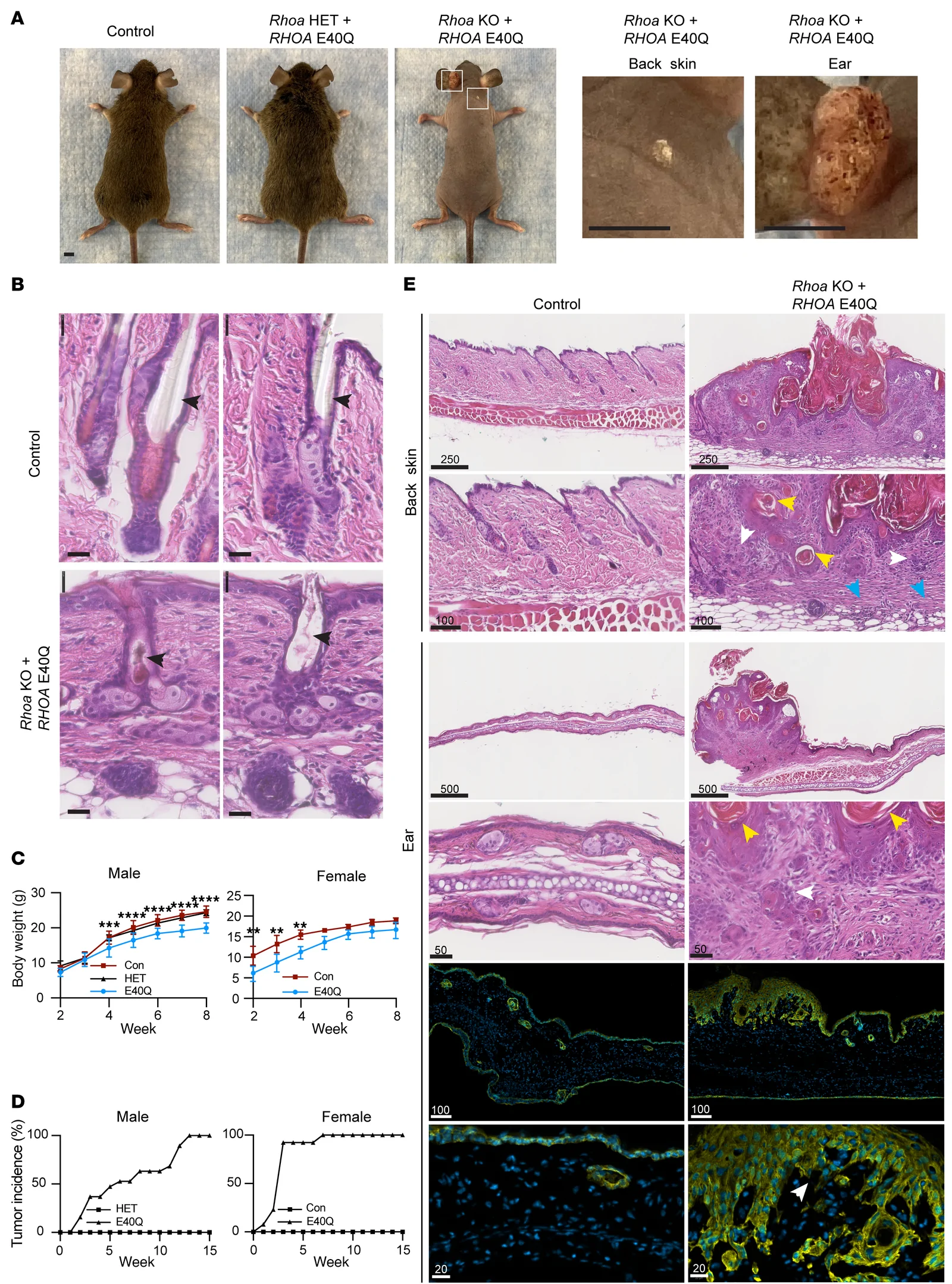
Mice expressing *RHOA* E40Q in keratinocytes in the absence of endogenous *Rhoa* develop SCC in the skin. Phenotype analysis of WT control, HET, and E40Q mice. (**A**) Representative images of indicated 8-week-old mice showing defective fur formation and tumor formation in ear and back skin of E40Q mice. Scale bar: 0.5 cm. (**B**) Representative images of H&E-stained sections of back skin of 8-week-old mice indicate malformed hair shafts in E40Q mice but not in controls (black arrowheads). Scale bar: 25 mm. (**C**) Body weight of indicated mice (*n* = 8/9/5 Control/HET/E40Q male mice; *n* = 4/4 Control/E40Q female mice). Error bars represent SD of the mean. Statistical significance was determined using 2-way ANOVA. (**D**) Tumor incidence of indicated mice (*n* = 15/19 Control/HET/E40Q male mice; *n* = 18/13 Control/E40Q female mice). (**E**) Representative images of H&E-stained sections of back skin and ears of 8-week-old mice and of ear sections stained for the keratinocyte marker keratin 5 (2 bottom rows). For E40Q mice, tumor-containing sections were selected. No tumors were present in control mice. The scale bar size (in mm) is indicated in the images. Yellow arrowheads: keratin pearls; white arrowheads: invasive cells; blue arrowheads: inflammatory infiltrate). **P < 0.01, ***P < 0.001, ****P < 0.0001.

**Figure 4:**
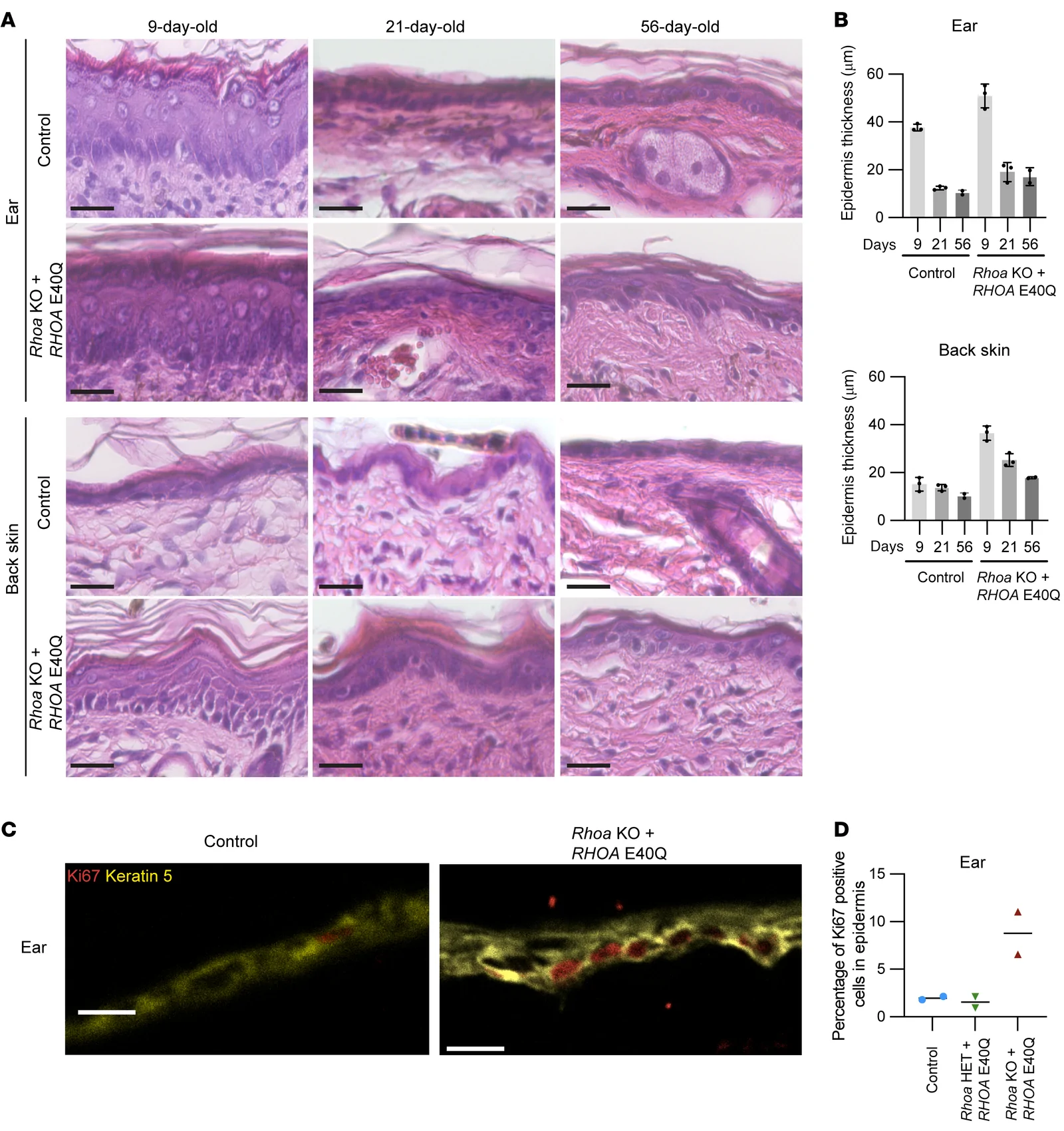
Epidermal hyperplasia in mice expressing *RHOA* E40Q in keratinocytes in the absence of endogenous *Rhoa*. (**A**) Representative images of H&E-stained tumor-free sections of ears and back skin of control mice at 9, 21, and 56 days old and E40Q mice. Scale bar: 25 mm. (**B**) Quantification of epidermal thickness in the mice in **A**. Data are reported as mean with SD (*n* = 3/3/2 mice analyzed at 9, 21, and 56 days of age, respectively). (**C**) Representative immunofluorescence images of ear sections from 56-day-old E40Q mice stained for Ki67 (red) and keratin 5 (yellow). Scale bars: 8 μm. (**D**) Quantification of percentage of Ki67-positive cells in the basal keratinocyte layer of indicated 56-day-old mice (*n* = 2/2/2 Control/HET/E40Q mice, respectively).

**Figure 5:**
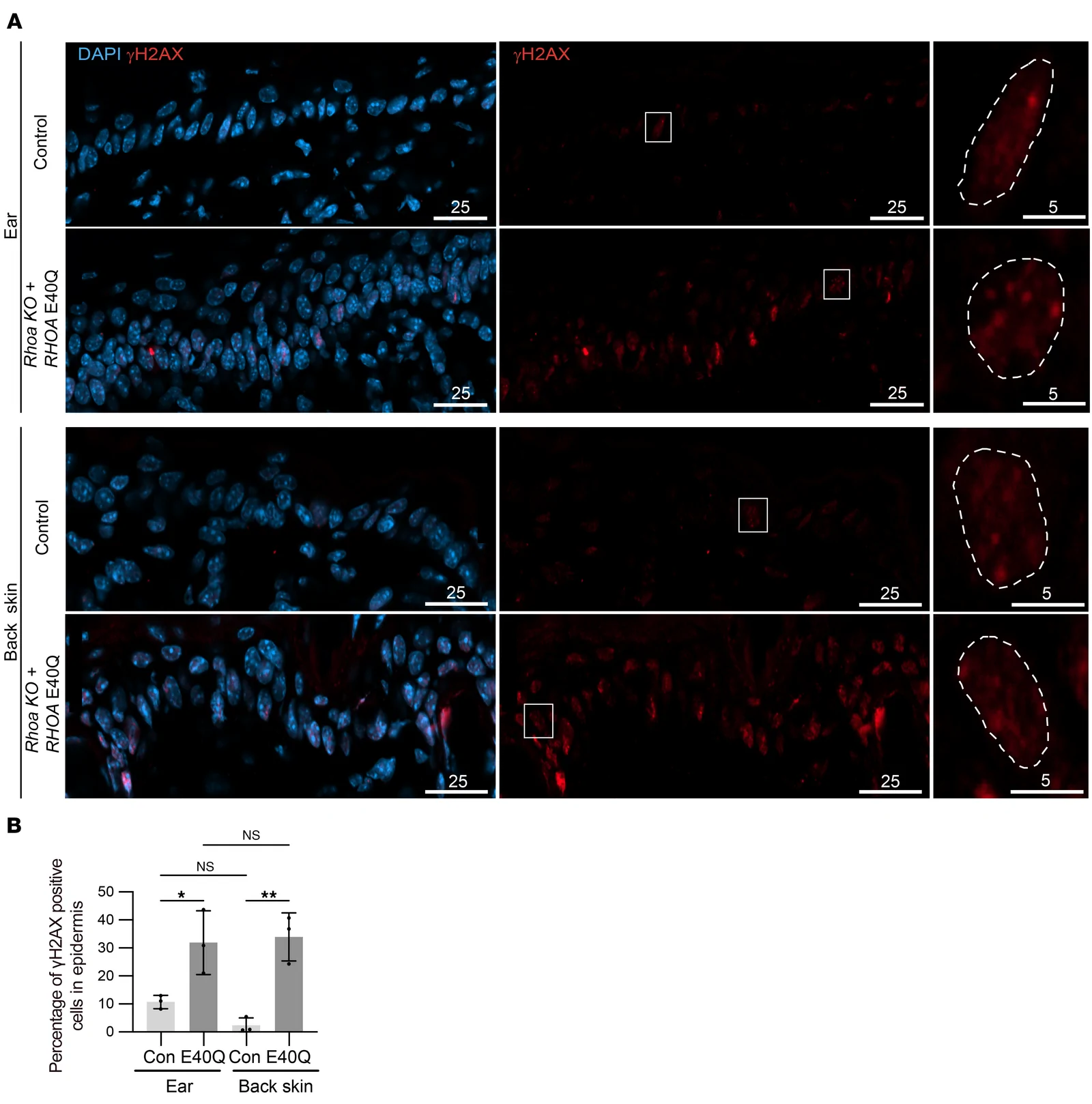
Increased frequency of γH2AX-positive cells in the epidermis of 9-day-old mice expressing *RHOA* E40Q in the absence of endogenous *Rhoa*. (**A**) Representative immunofluorescence images of ear and back skin sections from 9-day-old control and E40Q mice stained for γH2AX (red) and DAPI (blue). Scale bar size (in mm) is indicated in the images. (**B**) Quantification of the percentage of γH2AX-positive cells in the epidermis of ear and back skin sections shown in **A**. Data are presented as mean ± SD. Each dot represents 1 mouse. For each mouse, more than 200 nuclei were investigated. Statistical significance was determined by ordinary 1-way ANOVA (*n* = 3). *P < 0.05, **P < 0.01.

**Figure 6:**
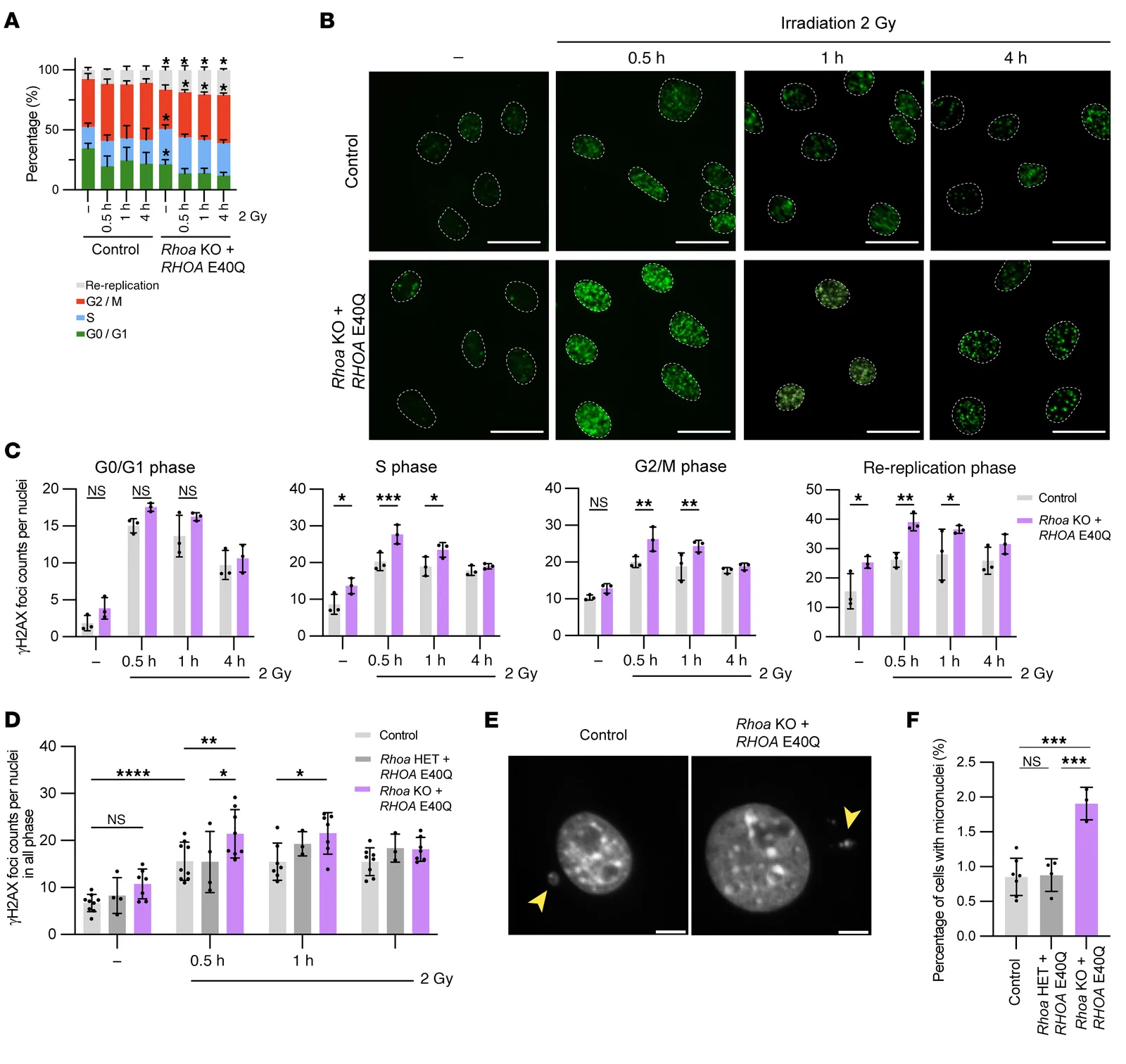
RhoA E40Q affects genome maintenance in primary keratinocytes. (**A**–**F**) Primary murine keratinocytes isolated from 3-week-old E40Q, HET, or control mice were irradiated with 2 Gy and analyzed at indicated time points. Data are presented as mean ± SD. Statistical significance was determined using 1-way ANOVA. (**A**) Cell cycle distribution (*n* = 3/3 Control/E40Q mice, respectively) (**B**) Immunofluorescence staining for γH2AX in primary murine keratinocytes. Nuclei were identified by DAPI and outlined with dashed white lines. Scale bar: 30 μm. (**C**) Quantification of γH2AX foci per nucleus analyzed according to cell cycle phase (*n* = 3/3 Control/E40Q mice, respectively). (**D**) Quantification of γH2AX foci across all cell cycle phases in primary murine keratinocytes (*n* = 9/9/3–4 Control/HET/E40Q mice, respectively). (**E**) Representative images of micronuclei in primary murine keratinocytes. Arrowheads indicate micronuclei. Scale bar: 20 μm. (**F**) Frequency of micronuclei in primary murine keratinocytes. Each dot represents keratinocytes derived from a single mouse. More than 1,000 cells were analyzed per mouse (*n* = 7/4/3 Control/HET/E40Q mice, respectively). *P < 0.05, **P < 0.01, ***P < 0.001, ****P < 0.0001.

**Figure 7:**
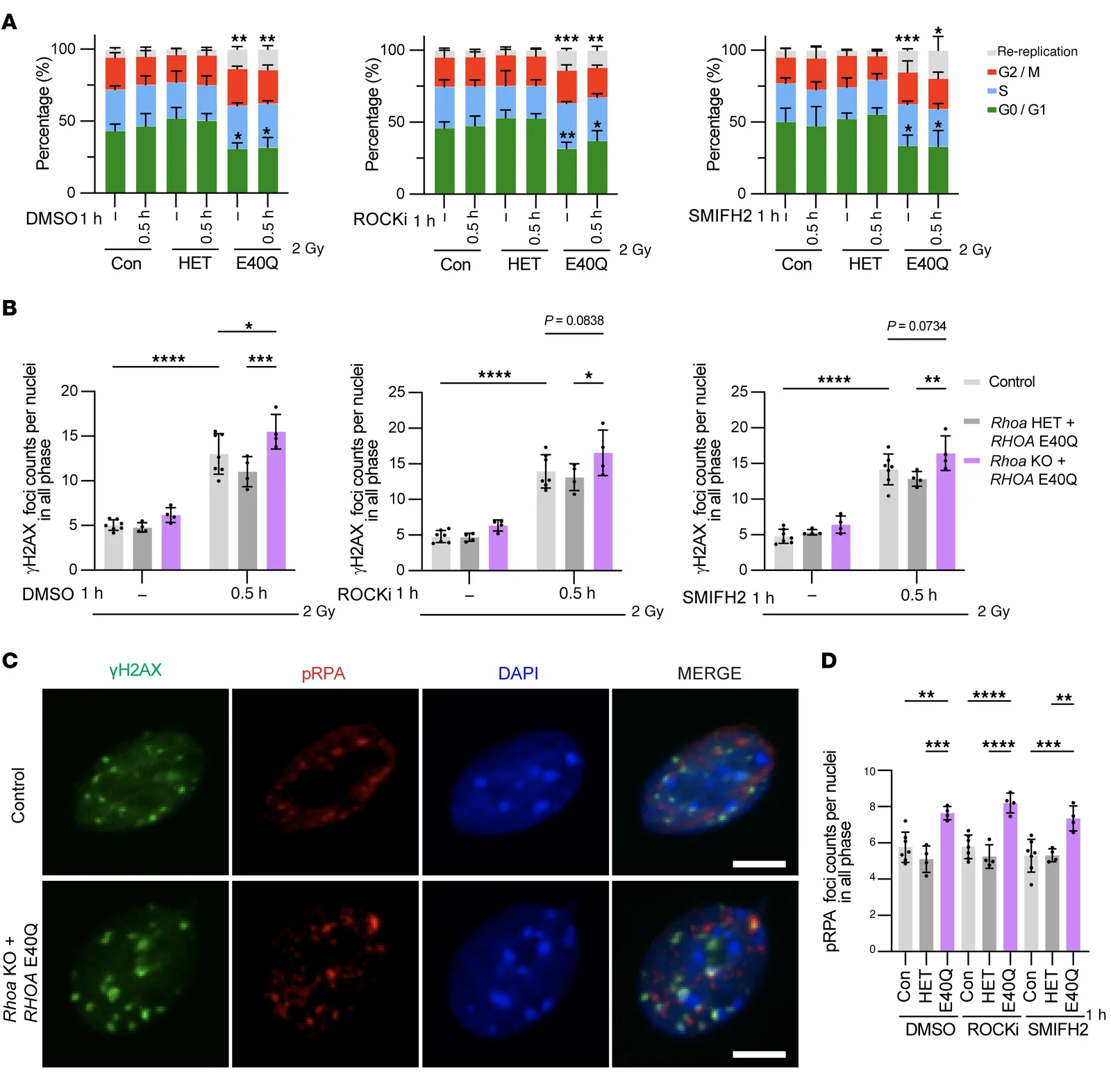
Replication stress induced by *Rhoa* E40Q is not rescued by inhibition of ROCK or formin signaling. (**A**) Cell cycle distribution of primary murine keratinocytes from control (Con), HET, and E40Q mice treated with DMSO, ROCKi Y-27632 (10 μM), or SMIFH2 (10 μM) for 1 hour prior to exposure to 2 Gy of irradiation. Cells were classified into G0/G1, S, G2/M, and re-replication phases based on DNA content and EdU incorporation (*n* = 7/4/4 Control/HET/E40Q mice, respectively). (**B**) Quantification of γH2AX foci in primary murine keratinocytes treated with DMSO, ROCKi, or SMIFH2 and exposed to 2 Gy of irradiation for 0.5 hours (*n* = 7/4/4 Control/HET/E40Q mice, respectively). (**C**) Representative images of γH2AX and pRPA immunofluorescence staining in primary murine keratinocytes treated with DMSO, ROCKi, or SMIFH2. Scale bars: 20 μm. (**D**) Quantification of pRPA foci in primary murine keratinocytes treated with DMSO, ROCKi or SMIFH2 (*n* = 7/4/4 Control/HET/E40Q mice, respectively). Each dot represents 1 mouse. More than 1,000 cells were analyzed per mouse. In all quantifications, data are presented as mean ± SD. Statistical significance was determined by 2-way ANOVA. *P < 0.05, **P < 0.01, ***P < 0.001, ****P < 0.0001.

**Figure 8:**
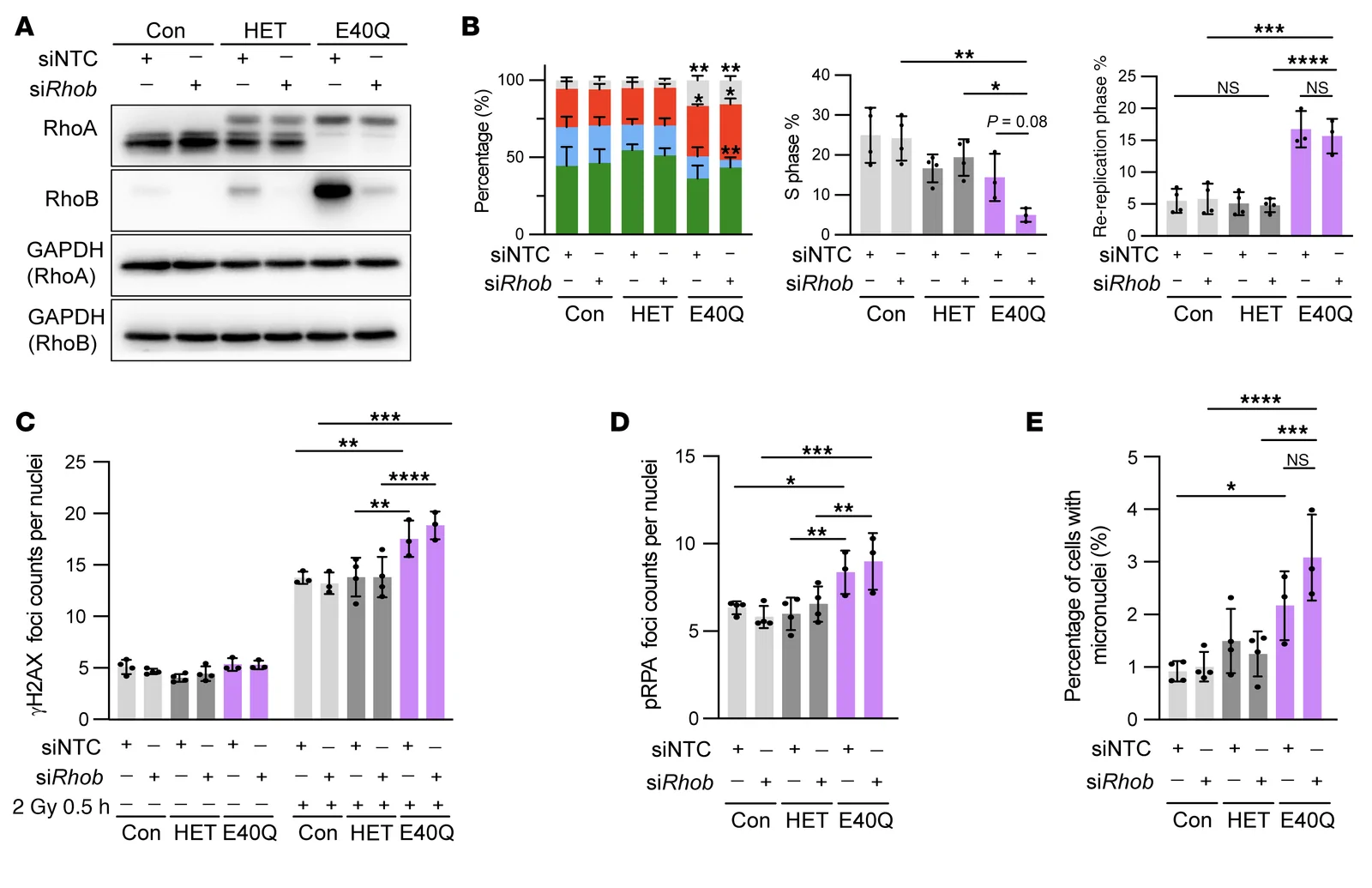
*Rhob* depletion reduces S-phase cells but does not rescue replication stress or genome instability in *RhoA* E40Q keratinocytes. (**A**) Representative blots showing RhoA and RhoB protein levels in primary murine keratinocytes transfected with nontargeting control siRNA (siNTC) or *Rhob*-targeting siRNA (si*Rhob*). GAPDH was used as a loading control (*n* = 3/3/3 Control/HET/E40Q mice, respectively). (**B**) Cell cycle phase distribution of primary murine keratinocytes transfected with siNTC nontargeting control or si*Rhob*. (**C**) Quantification of γH2AX foci counts per nuclei. Cells were exposed to 2 Gy of irradiation for 0.5 hours prior to analysis. (**D**) Quantification of pRPA foci counts per nuclei. (**E**) Quantification of percentage of cells with micronuclei. Each dot represents 1 mouse. In all quantifications, data are presented as mean ± SD. Statistical significance was determined using 2-way ANOVA (*n* = 3–4/3–4/3 Control/HET/E40Q mice, respectively). *P < 0.05, **P < 0.01, ***P < 0.001, ****P < 0.0001.

**Figure 9:**
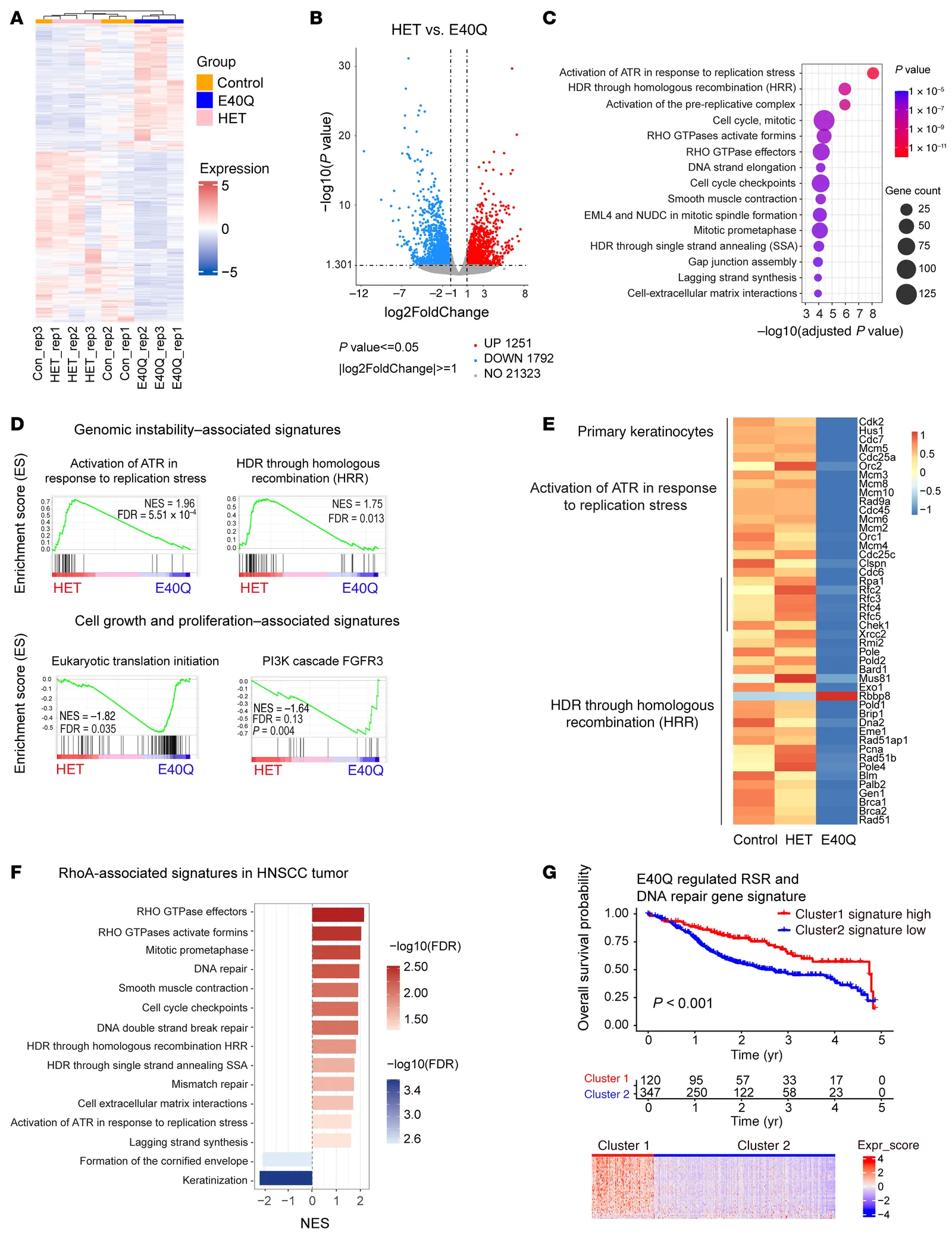
Transcriptomic analyses of E40Q primary keratinocytes and HNSCC tumor. (**A**) Heatmap of significant DEGs among primary murine keratinocytes from WT (control), HET, and E40Q mice. (**B**) Volcano plot of significantly DEGs in HET versus E40Q. (**C**) Top 15 reactome-defined enriched gene sets (*P* < 0.05) among significantly DEGs in E40Q compared with the HET primary murine keratinocytes. (**D**) GSEA of genomic instability and cell growth–related signatures comparing HET versus E40Q primary murine keratinocytes. (**E**) Heatmap showing the expression of significantly dysregulated genes involved in the replication stress response (RSR) and DNA repair pathway. (**F**) Indicated gene sets that are significantly positively (NES > 0, FDR < 0.05) or negatively (NES < 0, FDR < 0.05) associated with *RHOA* expression in patient transcriptomic data. (**G**) Overall survival (upper panel) and mRNA expression (lower panel) of patients with HNSCC classified by the E40Q-regulated RSR and DNA repair gene signature.

## Version 1 08/25/2026

In-Press Preview

## Version 2 10/15/2026

Electronic publication
