# Supplementary material for: RhoA WT allele loss unleashes the cancer driver function of RhoA E40Q
Source: J Clin Invest. 2026 Aug 25;136(20):e201975. doi: 10.1172/JCI201975 (PMC13668625; doi:10.1172/JCI201975)
Supplement: Supplementary file 1 [file jci-136-201975-s152.pdf]

Supplemental Material

RhoA wild-type allele loss unleashes the cancer driver function of RhoA E40Q

Justine Noujarède[1,*], Qiuyue Wang[1,*], Eleftherios Panagiotis Kokkinogenis[1], Yuewan Luo[1], Ivona Cudina[1], Simon Willaume[1], Clémence Mooser[1], Lap Phuoc Nguyen[1,2], Mads Frederik Poulsen[1],Simon Heijmerikx[1], Thu Han Le Phan[1], Xiubin He[1], Jesper Bøje Andersen[1], Claus Storgaard Sørensen[1], Cord Brakebusch[1,**]

[1]University of Copenhagen, Biotech Research and Innovation Center (BRIC), Ole Maaløes Vej 5, 2200 Copenhagen, Denmark; [2]Current address Faculty of Basic Sciences, Can Tho University of Medicine and Pharmacy, Can Tho City, Vietnam;

*equal contribution; **corresponding author. Email: cord.brakebusch@bric.ku.dk

This PDF file includes:

Supplemental Figures 1-17

Supplemental Tables 1-3

Supplemental Methods

References

Supplemental Figures

Supplemental Figure 1

A

TCGA Patient ID	*RHOA* point mutation	Domain	Copy nr	MYC AMP	PIK3CA AMP	PIK3CB AMP	FGF4 AMP
CN-6998	*E40Q*	Switch I	HET DEL				
CV-7102	*E40Q*	Switch I	HET DEL				
CV-7410	*E40Q*	Switch I	HET DEL				
DQ-5625	*E40Q*	Switch I	Diploid				
CN-A6V3-01	*T37S*	Switch I	HET DEL				
BB-8596-01	*G62E*	Switch II	HET DEL				
F7-A624-01	*F106L*		Diploid				
TN-A7HL-01	*R145T*		Diploid				
KU-A66S-01	*K164N*		Diploid				

B

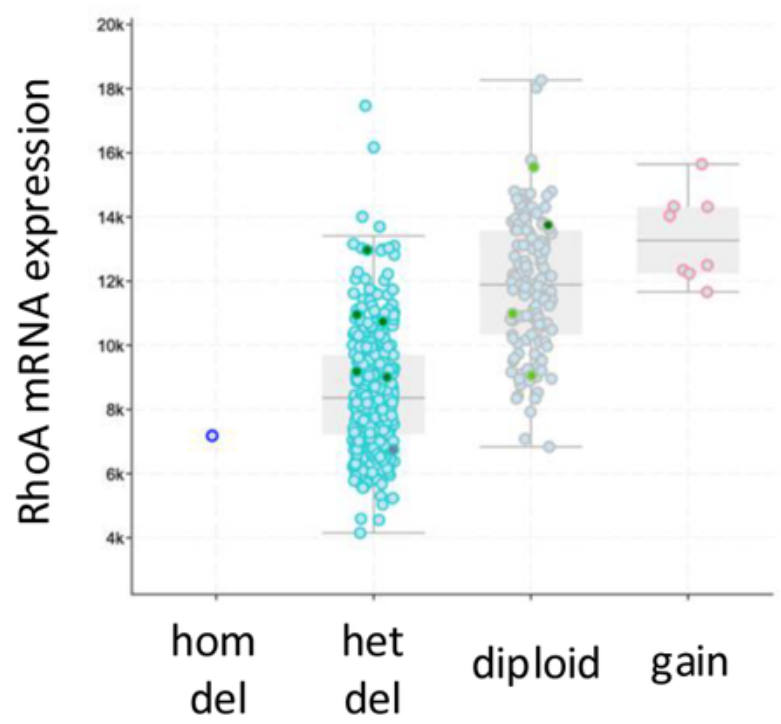

C

TCGA HNSCC	n		*MYC* AMP
All	496	Frequency	9.3%
E40Q	4	Frequency	75%
		Enrichment	8.1
		p value (Welch's t-test)	7.7 E-2
E40Q, T37S, G62E	6	Frequency	67%
		Enrichment	7.1
		p value (Welch's t-test)	4.1 E-2

D

RHOA signaling gene	*PREX2*	*RHPN1*	*ECT2*	*ARHGAP31*	*MYLK*	*ARHGEF17*
AMP frequency	3%	8%	14%	3%	3%	4%
Co-AMP driver gene	MYC	MYC	PIK3CA	PIK3CA	PIK3CA	FGF3
Co-AMP frequency	79%	79%	97%	86%	86%	95%

Supplemental Figure 1. *RHOA* E40Q HNSCC of the TCGA Pancancer Atlas are frequently associated with MYC amplification. (A) Features of HNSCC with point mutations in *RHOA*. **(B)** RhoA mRNA expression in HNSCC with respect to copy number alterations of *RHOA* gene (Dark green dots: missense mutation with suspected driver mutation; light green dots: missense mutation of unknown significance; light blue circle: heterozygous deletion; dark blue circles: homozygous deletion; red circles: gain). **(C)** Enrichment of MYC amplification in HNSCC with the *RHOA* point mutations E40Q, T37S, and G62E. Statistical significance was determined by Welch's t-test. **(D)** Co-amplification of Rho GTPase regulators and effectors with known oncogenes in HNSCC.

Supplemental Figure 2

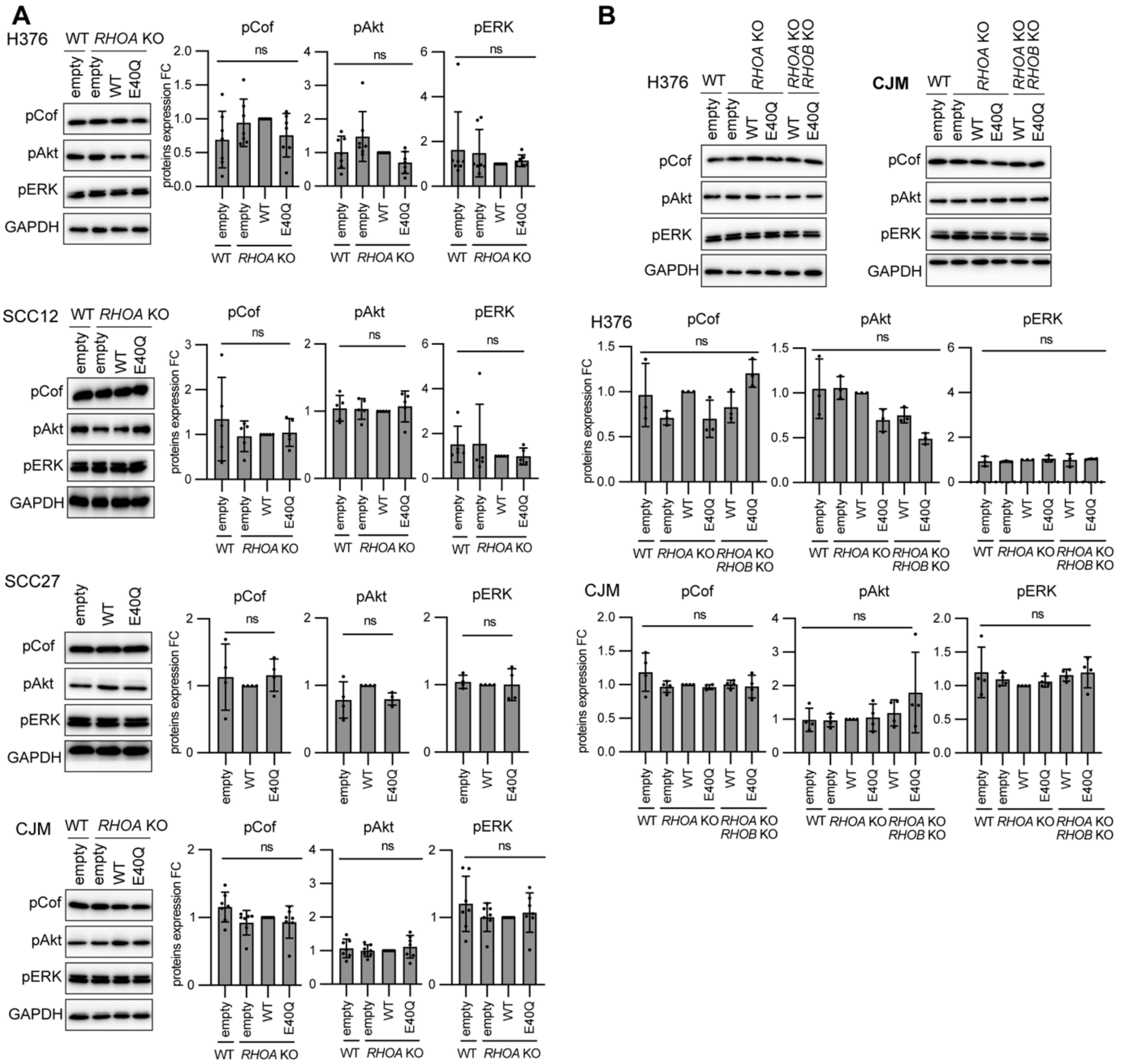

Supplemental Figure 2. RhoA E40Q is not affecting pCofilin, pERK, and pAkt in HNSCC cell lines. Quantification and representative examples of Western blots for pCofilin (pCof), pAkt, pERK, and GAPDH (loading control) with indicated cell types. Shown are wild type (WT) transduced with empty vector (empty), or *RHOA* knockout (*RHOA* KO) transduced with empty, *RHOA* WT (WT), or *RHOA* E40Q (E40Q). Data are shown as fold change relative to (*RHOA* KO + *RHOA* WT). Error bars represent standard deviation (SD) of the mean. Statistical significance was determined using one-way ANOVA **(A)** (n (H376): 7; n (SCC12): 5; n (SCC27): 4; n (CJM): 7). **(B)** As additional cell types, (*RHOA* KO *+ RHOA* WT) and (*RHOA* KO *+ RHOA* E40Q) with a *RHOB* KO were tested. Data for WT and *RHOA* KO were also included in (A) (n (H376): 3; n (CJM): 4).

Supplemental Figure 3

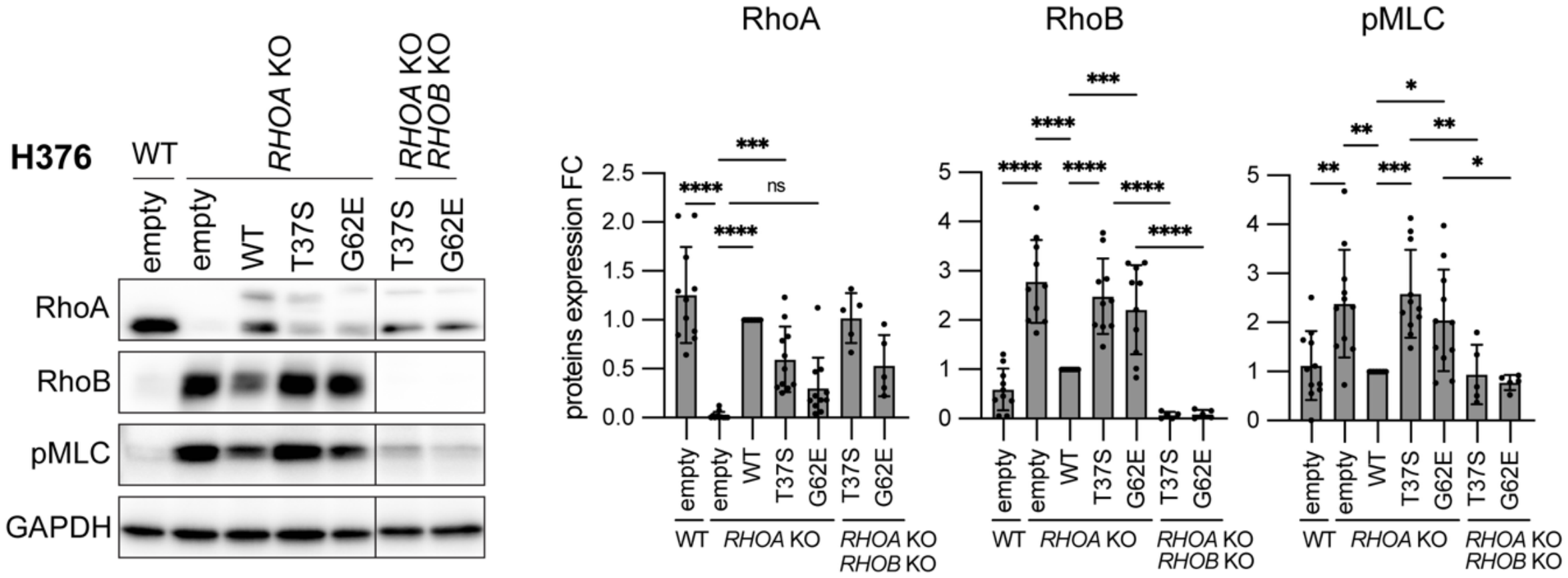

Supplemental Figure 3. RhoA T37S and RhoA G62E show similar signaling effects as RhoA KO. Representative Western blots of RhoA, RhoB, pMLC, and GAPDH in H376 WT, *RHOA* KO, or (*RHOA* KO + *RHOB* KO) cells transduced with empty vector, *RHOA* WT, *RHOA G62E*, or *RHOA T37S*. Data are shown as fold-change relative to (*RHOA* KO + *RHOA* WT). Quantification and representative examples of Western blots for RhoA, RhoB, pMLC, and GAPDH for the indicated cells. Error bars represent SD of the mean. Statistical significance was determined using one-way ANOVA (n: 5-6).

Supplemental Figure 4

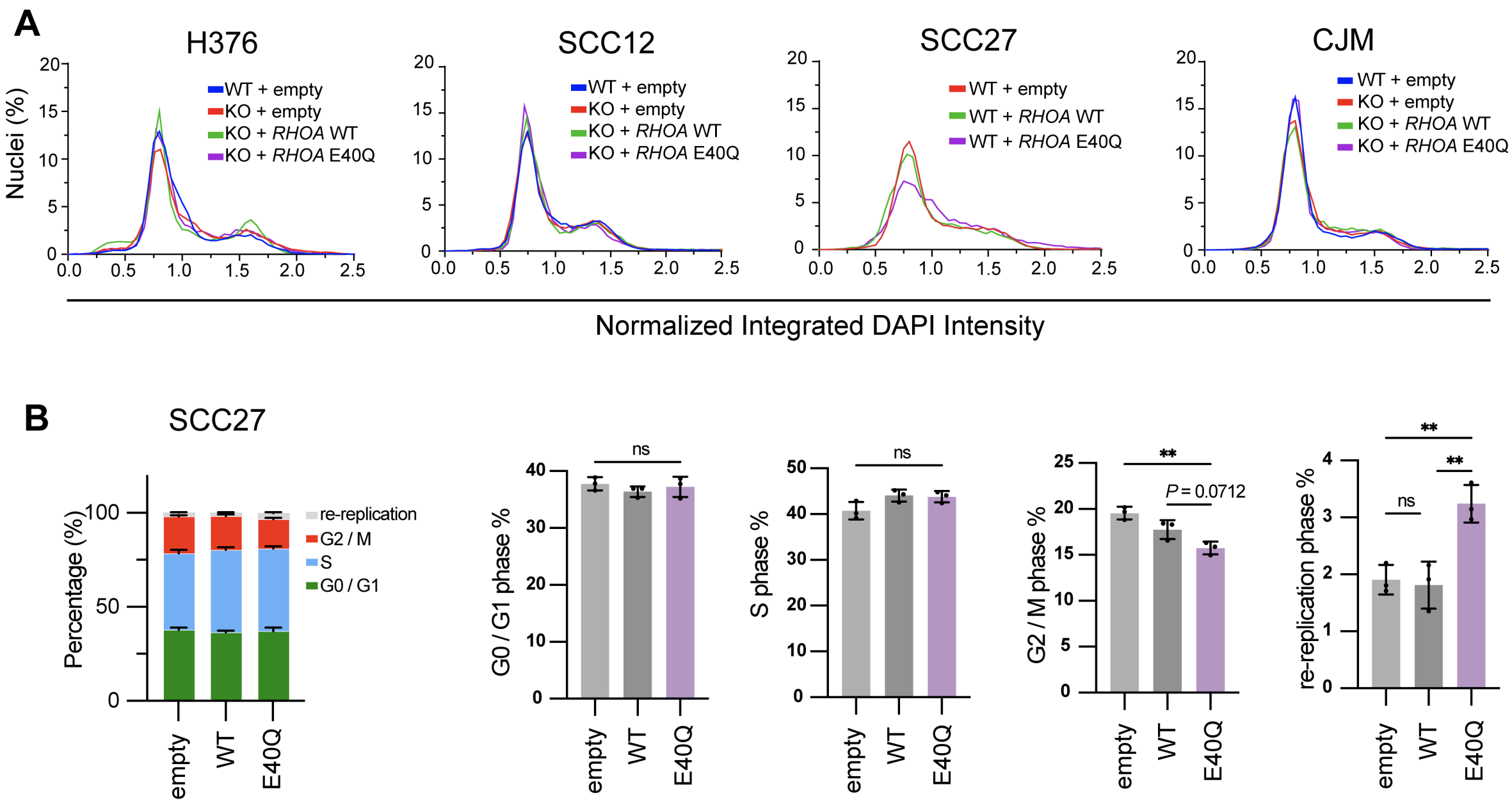

Supplemental Figure 4. RhoA E40Q is not strongly affecting cell cycle. (A) Cell cycle analysis by DAPI staining for the indicated cell lines. Integrated DAPI intensity values were normalized for (WT + empty) cells of each experiment (n: 9-15). **(B)** Quantification of cell cycle distribution in SCC27 cells transduced with empty vector, *RHOA* WT, or *RHOA* E40Q. Cells were classified as G0/G1, S phase, G2/M, or re-replicating populations. Data represent mean ± SD. Statistical significance was determined by one-way ANOVA (n:3).

Supplemental Figure 5

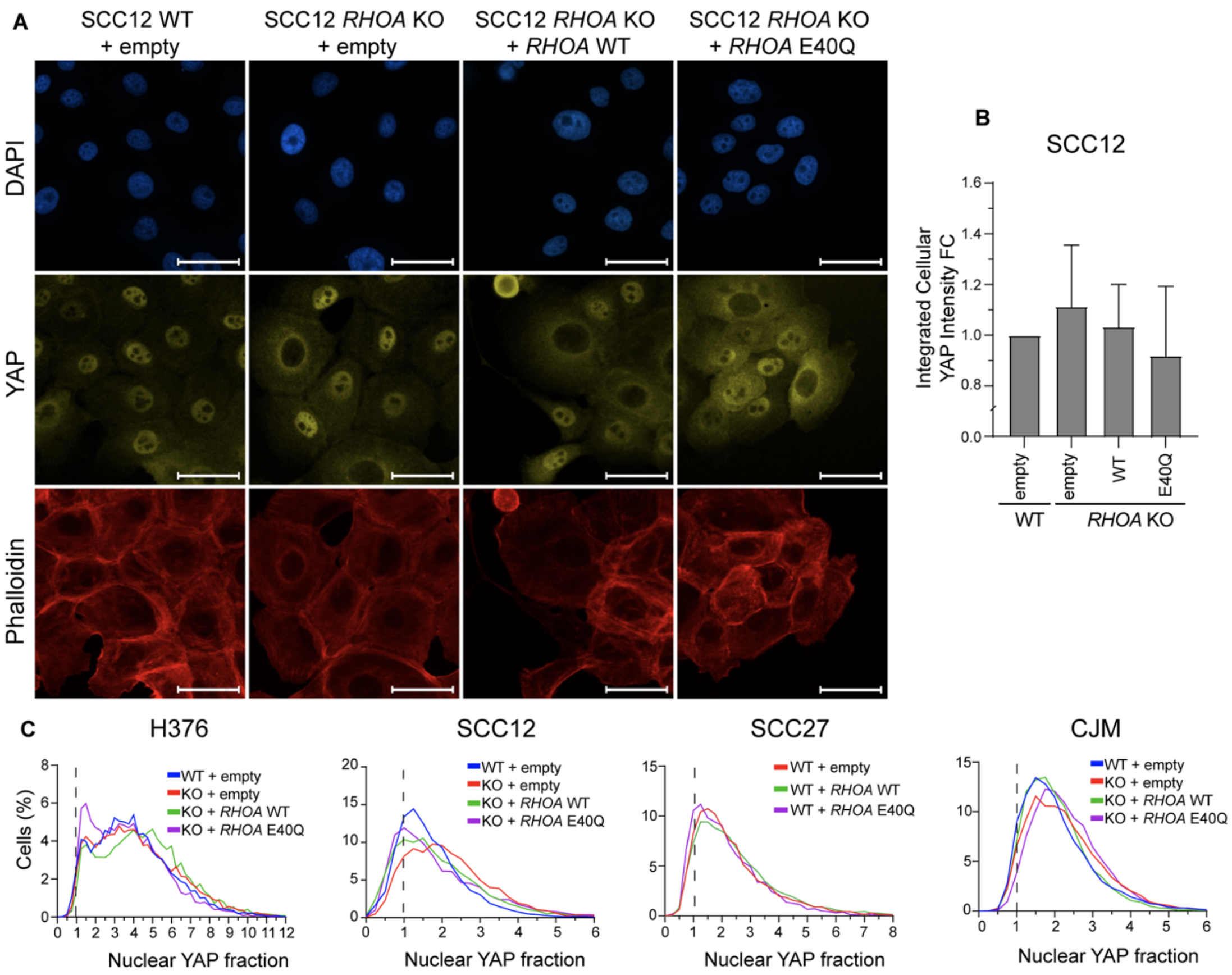

Supplemental Figure 5. RhoA E40Q does not strongly influence expression and nuclear fraction of YAP. SCC12 cells transduced with empty vector and *RHOA* KO SCC12 transduced with empty vector, *RHOA* WT, or *RHOA* E40Q, were analyzed by confocal fluorescence microscopy after staining for YAP, DNA (DAPI), and F-actin (fluorescently labelled phalloidin). **(A)** Representative images. Scale bar: 50 µm. **(B)** Quantification of cellular YAP integrated intensity in the indicated cell lines, shown as fold change (FC) relative to WT + empty. Error bars indicate SD (n: 3). **(C)** Quantification of nuclear fraction of YAP for the indicated cell lines. Dashed line indicates equal distribution between nucleus and cytoplasm (n: 3-5).

Supplemental Figure 6

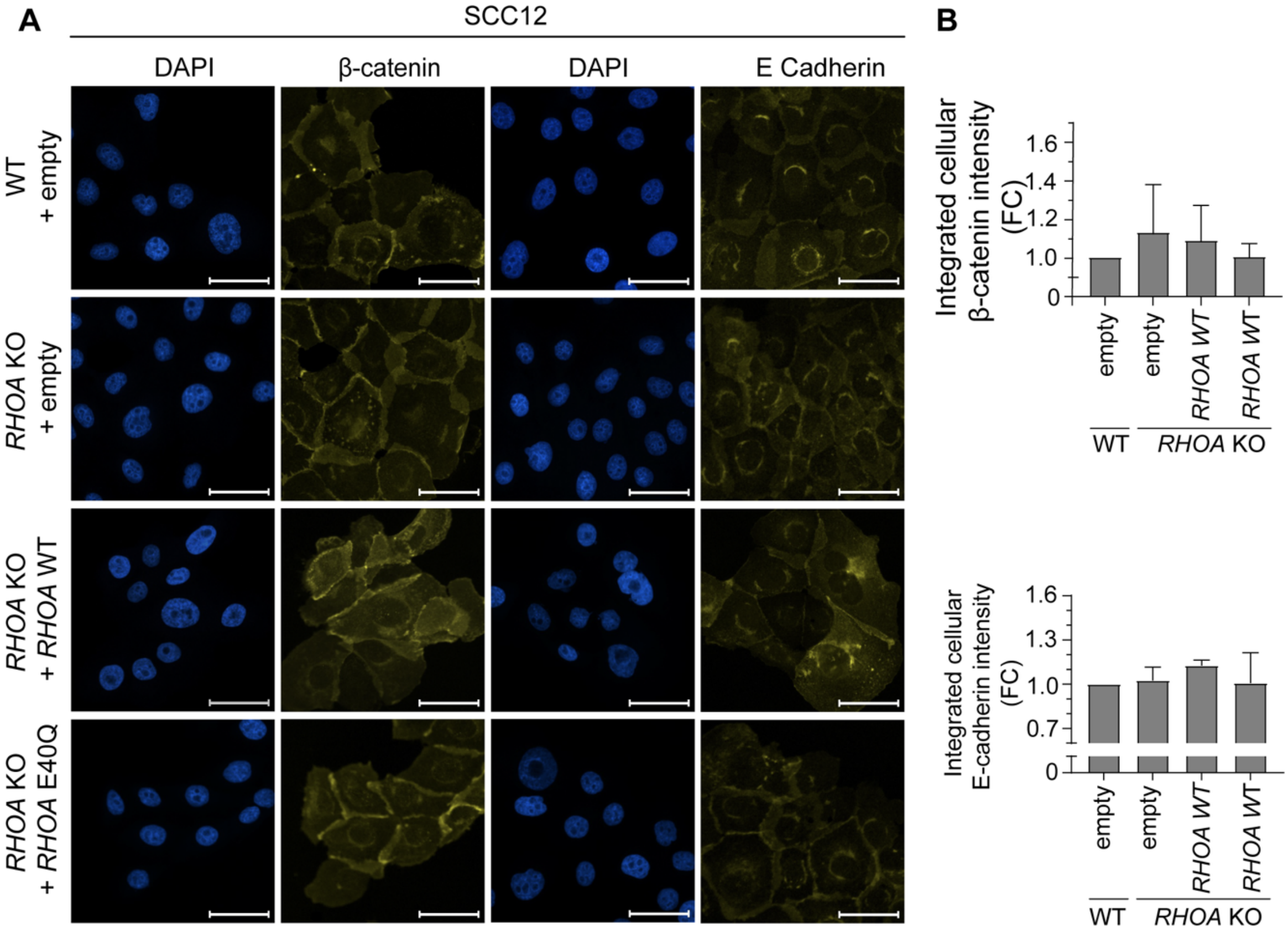

Supplemental Figure 6. RhoA E40Q is not altering expression of the epithelial markers β-catenin and E-cadherin in SCC12 cells. SCC12 cells transduced with empty vector and *RHOA* KO SCC12 transduced with empty vector, *RHOA* WT, or *RHOA* E40Q, were analyzed by confocal fluorescence microscopy after staining for DNA (DAPI), β-catenin, and E-cadherin. **(A)** Representative images. Scale bar: 50 µm. **(B)** Quantification of integrated cellular intensities. Error bars represent SD of the mean. Statistical significance was determined using one-way ANOVA (n: 3).

Supplemental Figure 7

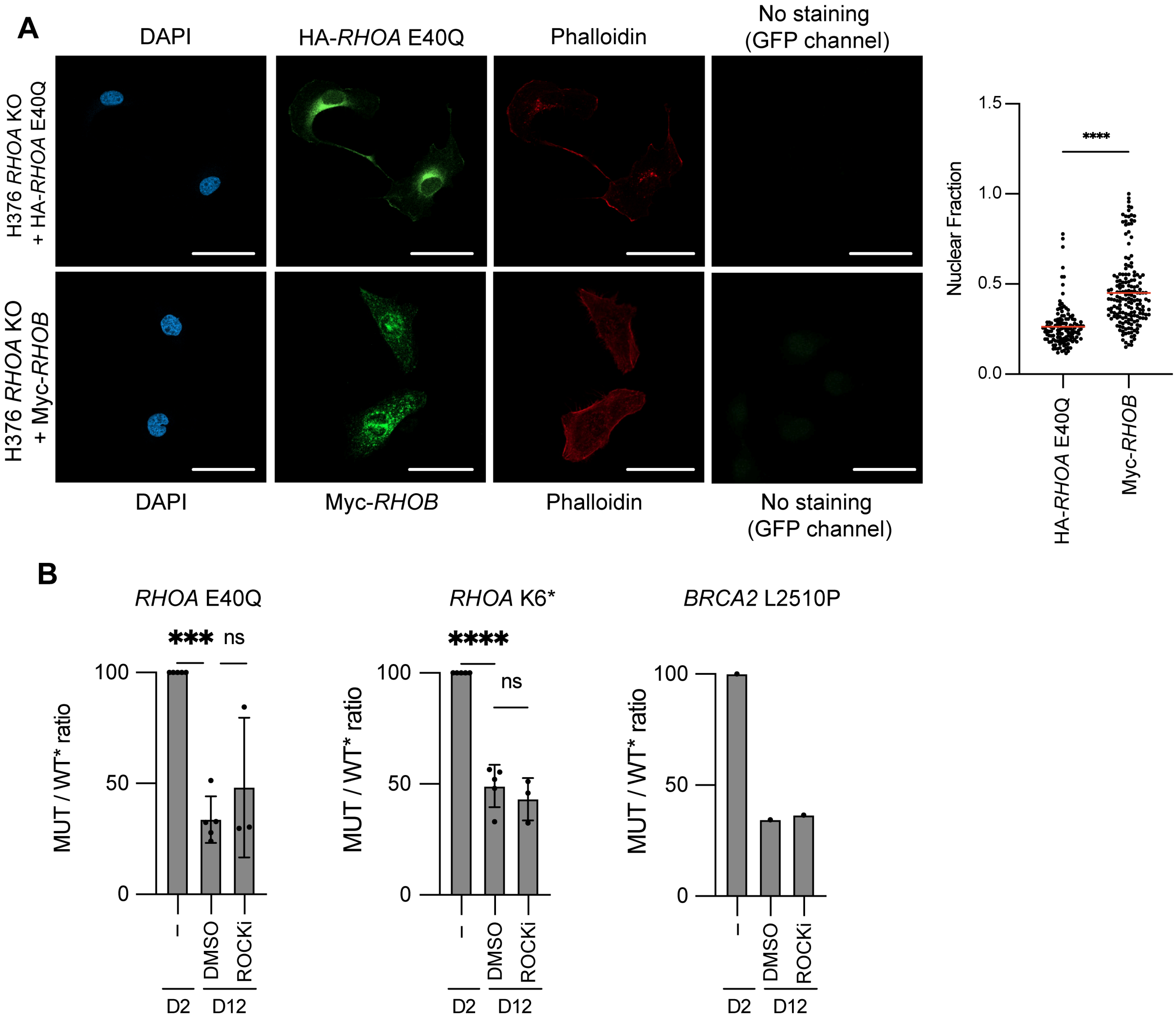

Supplemental Figure 7. RhoA E40Q reduces cellular fitness in breast epithelial cells. (A) *RHOA* KO H376 cells transduced with Myc-*RHOB* or HA-*RHOA* E40Q were stained for DNA (DAPI), HA, Myc, F-actin (fluorescently labelled phalloidin), or left unstained and analyzed by confocal fluorescence microscopy. Scale bar: 50 µm. Shown are representative images and quantification of the nuclear fraction of HA- *RHOA* E40Q and Myc- *RHOB*. Error bars represent SD of the mean. Statistical significance was assessed using an unpaired Student's t-test. Each dot represents a single cell. Shown are the results of 4 independent experiments (n (HA- *RHOA* E40Q): 133; n (Myc- *RHOB*): 175). **(B)** CRISPR-Select^{TIME} assay testing cellular fitness of breast epithelial iCas9-MCF10A cells with the indicated point mutations introduced to the endogenous genes. Fitness was compared in a competitive growth assay to WT cells with a silent mutation in the *RHOA* gene (WT'). MUT/WT' ratio was measured at day 2 (D2) and day 12 (D12) after transfection, with or without 10 µM ROCK inhibitor treatment. Error bars

represent SD. Statistical significance was determined using one-way ANOVA (n (*RHOA* E40Q, *RHOA* K6*): 5; n (*BRACA2* L2610P): 1).

Supplemental Figure 8

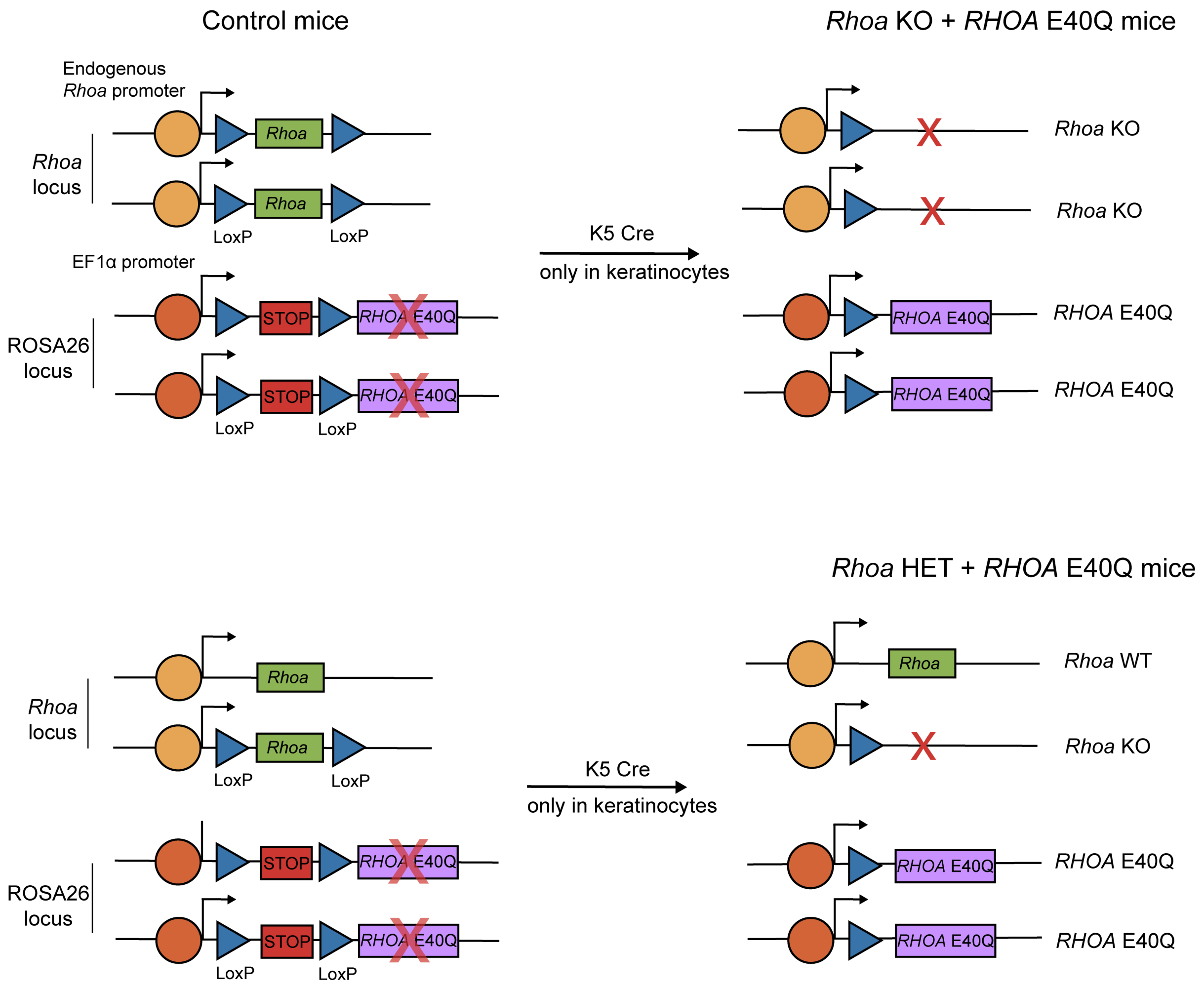

Supplemental Figure 8. Schematic presentation of genome of genetically modified mice. Shown are control, (*Rhoa* KO + *RHOA* E40Q), and (*Rhoa* HET + *RHOA* E40Q) mice. Both the *Rhoa* and the ROSA26 locus were modified. The endogenous *Rhoa* gene was changed to a conditional KO and a conditional knock-in for *RHOA* E40Q was introduced into the ROSA26 locus. In the absence of Cre recombinase, normal levels of endogenous *Rhoa* are produced but no *RHOA* E40Q (control mice). In the presence of a keratinocyte restricted Cre recombinase (K5Cre), the endogenous *Rhoa* is deleted and *RHOA* E40Q is expressed in keratinocytes (*Rhoa* KO + *RHOA* E40Q). In (*Rhoa* HET + *RHOA* E40Q) mice, *RHOA* E40Q is expressed at a similar level as in (*Rhoa* KO + *RHOA* E40Q), but one RhoA WT allele is present.

Supplemental Figure 9

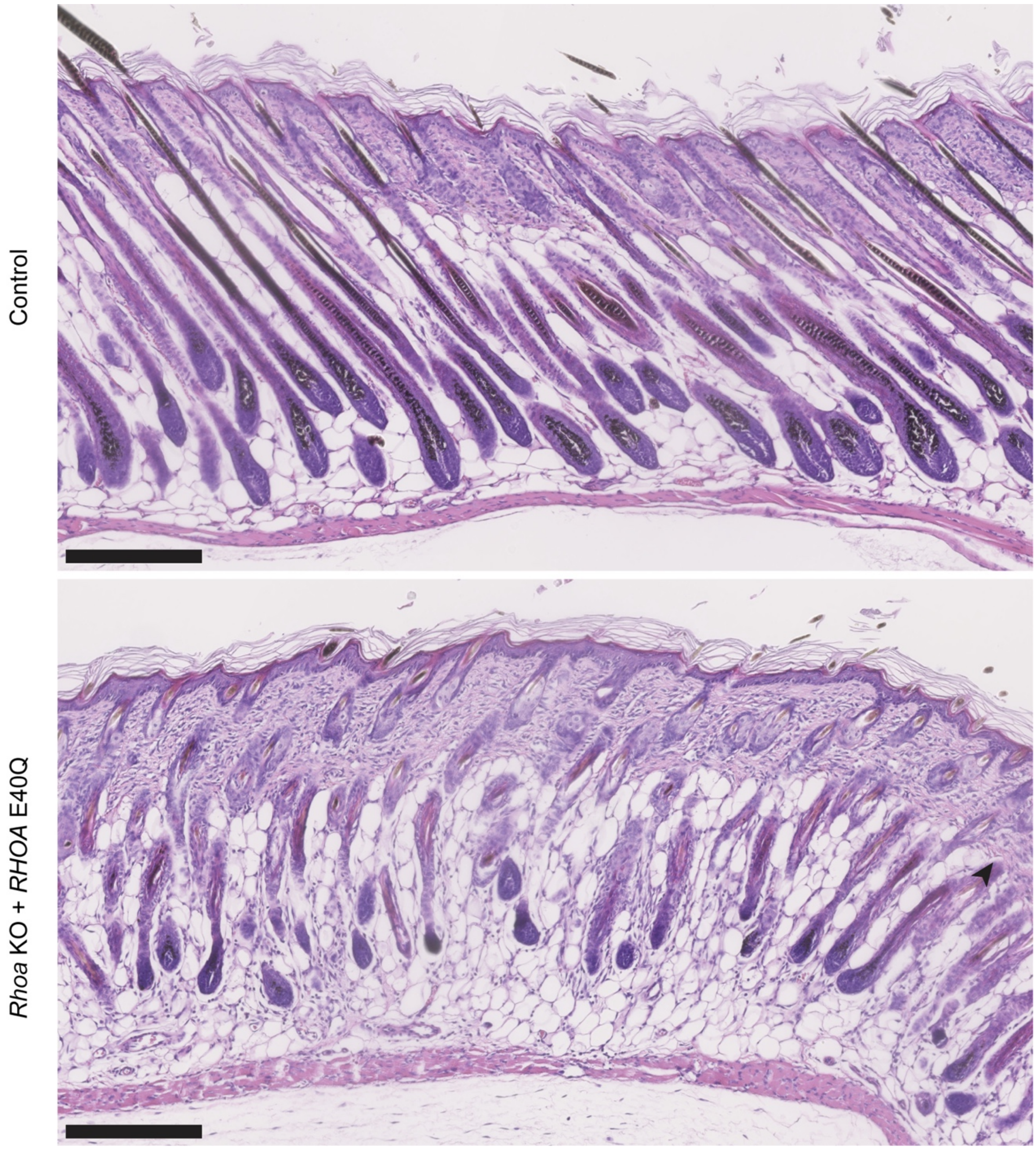

Supplemental Figure 9. Altered hair follicle morphology in 9 d old (*Rhoa* KO + *RHOA* E40Q) mice. Representative H/E images of back skin from 9 d old Control and (*Rhoa* KO + *RHOA* E40Q) mice. Altered hair shaft morphology and hair follicle abnormalities were observed in (*Rhoa* KO + *RHOA* E40Q) skin, whereas only focal inflammatory infiltrates were detected. Scale bars: 250 µm.

Supplemental Figure 10

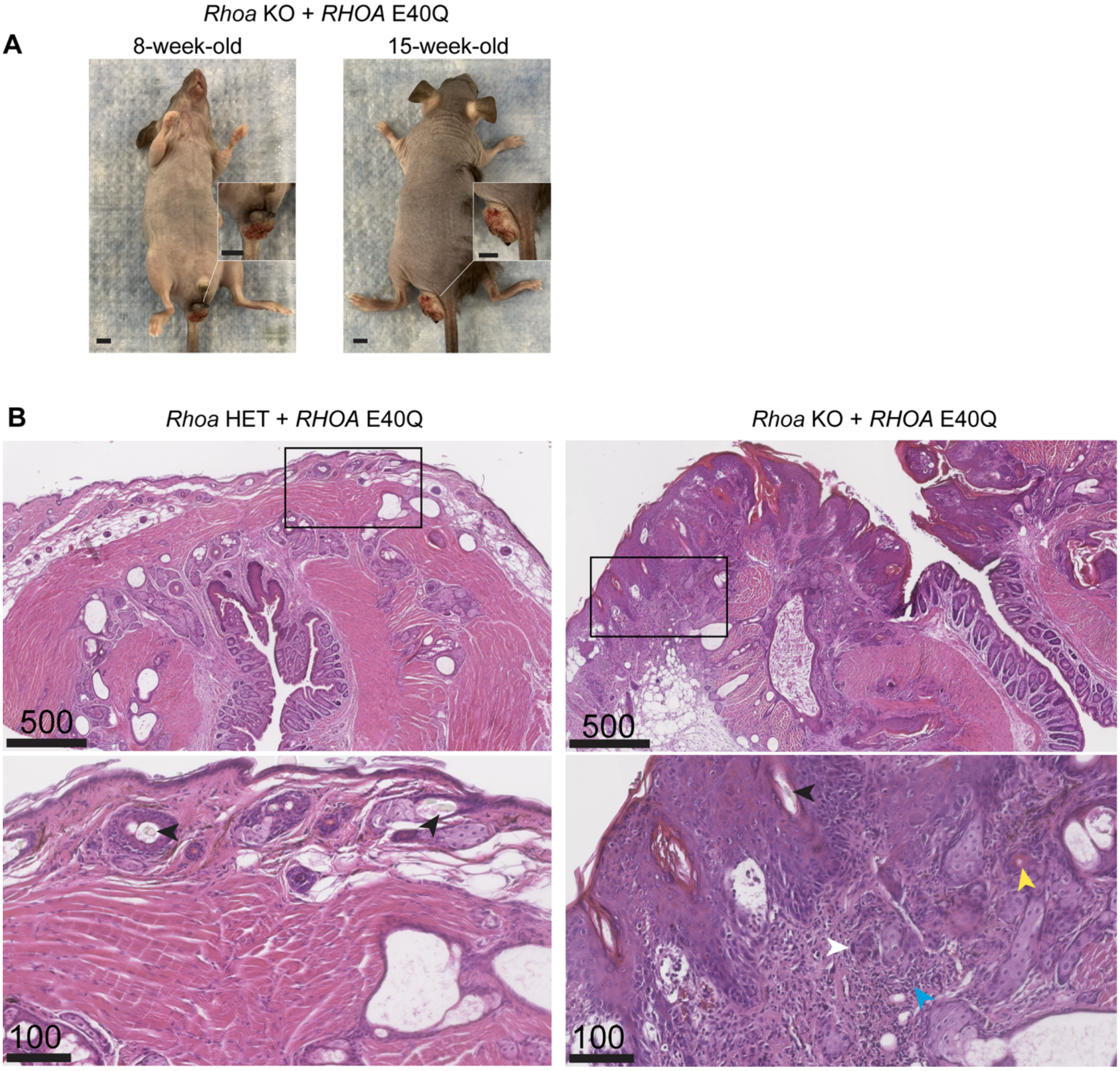

Supplemental Figure 10. Perianal squamous cell carcinoma in (*Rhoa* KO + *RHOA* E40Q) mice. (A) Representative image of a (*Rhoa* KO + *RHOA* E40Q) mice showing a tumor in the perianal region. An enlarged view of the tumor is shown in the inset. Scale bar: 0.5 cm. **(B)** H/E images of the perianal region from a (*Rhoa* HET + *RHOA* E40Q) mouse and of a perianal tumor from a (*Rhoa* KO + *RHOA* E40Q) mouse, both 8 w old. Scale bar size in mm is indicated in the images (Black arrowheads: hair shafts; yellow arrowhead: keratin pearls; white arrowhead: invasive cells; blue arrowhead: inflammatory infiltrate).

Supplemental Figure 11

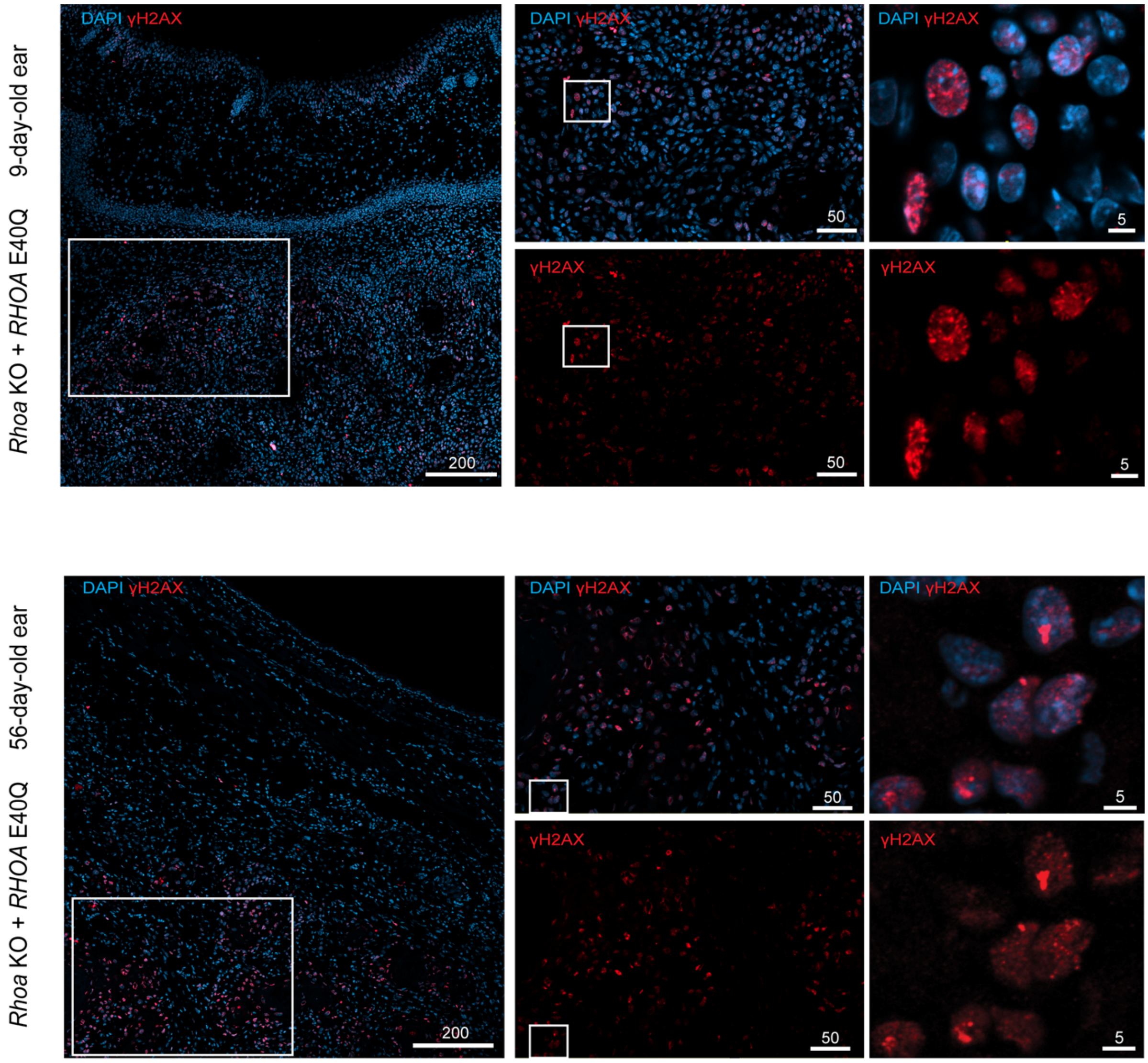

Supplemental Figure 11. High frequency of cells with high number of nuclear γH2AX foci in ear cancers from (*Rhoa* KO + *RHOA* E40Q) mice. Representative confocal images of γH2AX immunofluorescence staining of ear tumors from 9 d old and 56 d old (*Rhoa* KO + *RHOA* E40Q) mice. Insets indicate progressively enlarged views of the boxed regions.

Supplemental Figure 12

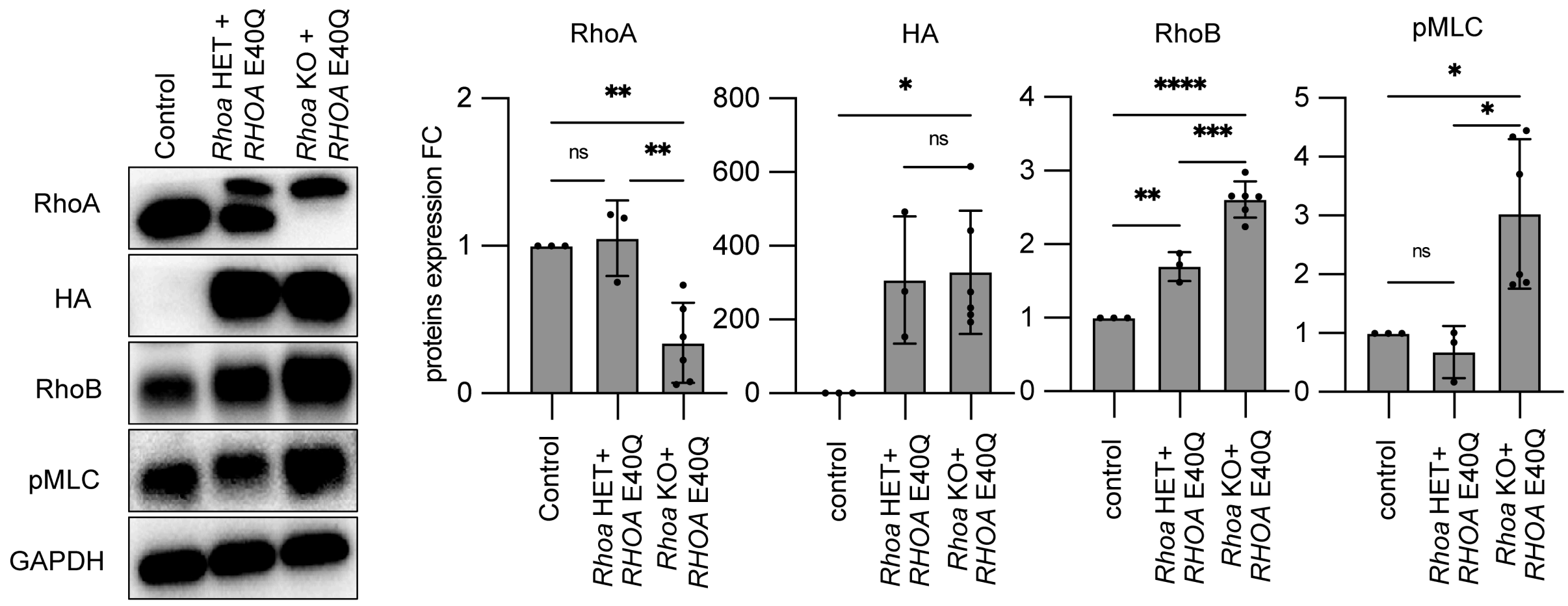

Supplemental Figure 12. Increased RhoB expression and pMLC levels in primary keratinocytes expressing *RHOA* E40Q in the absence of endogenous *Rhoa*. Representative blots and quantification of RhoA, HA-tagged RhoA E40Q, RhoB, and pMLC in Control, (*Rhoa* HET + *RHOA* E40Q), and (*Rhoa* KO + *RHOA* E40Q) primary murine keratinocytes. Data were normalized to the GAPDH loading control and to keratinocytes from control mice. Data are presented as mean ± SD. Statistical significance was determined using one-way ANOVA. Each dot represents one mouse (n: 3/3/6).

Supplemental Figure 13

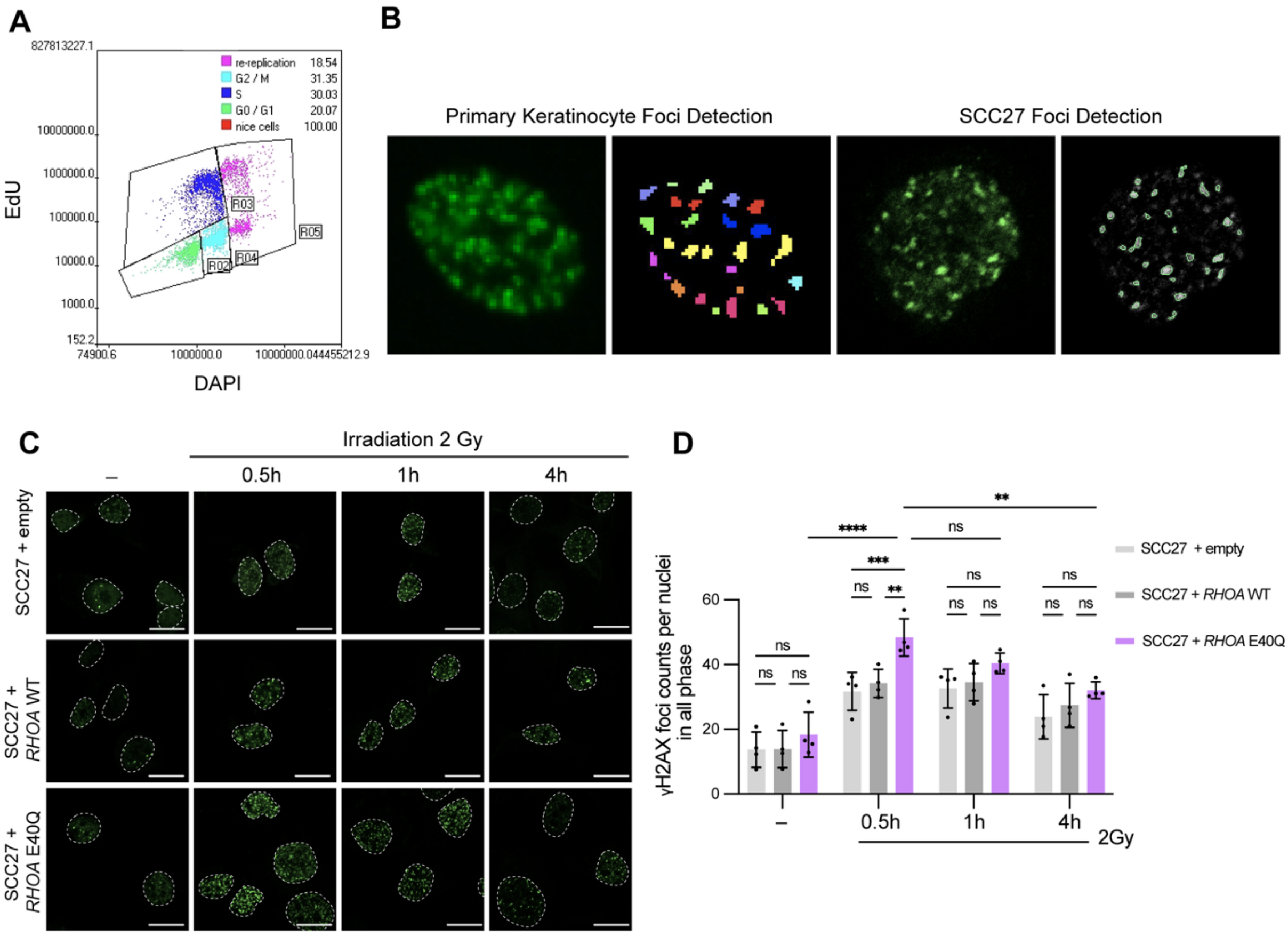

Supplemental Figure 13. Cell cycle analysis workflow and γH2AX foci quantification. (A) Representative example of cell cycle analysis showing the gating strategy used to classify cells into G0/G1, S, G2/M, and re-replication populations based on DAPI and EdU incorporation. **(B)** Representative examples of γH2AX foci detection. Left panels show γH2AX foci identification in primary murine keratinocytes using ScanR Analysis Software (Olympus). Right panels show γH2AX foci identification in SCC27 cells using CellProfiler. **(C)** Representative immunofluorescence images of γH2AX staining in SCC27 cells transduced with empty vector, *RHOA* WT, or *RHOA* E40Q, which were irradiated with 2Gy and analyzed at indicated time points. Nuclei as identified by DAPI were indicated by dashed white lines. Scale bar: 20 µm. **(E)** Quantification of γH2AX foci across all cell cycle phases in SCC27 cells (n: 3). Data represent mean ± SD. Statistical significance was determined using two-way ANOVA.

Supplemental Figure 14

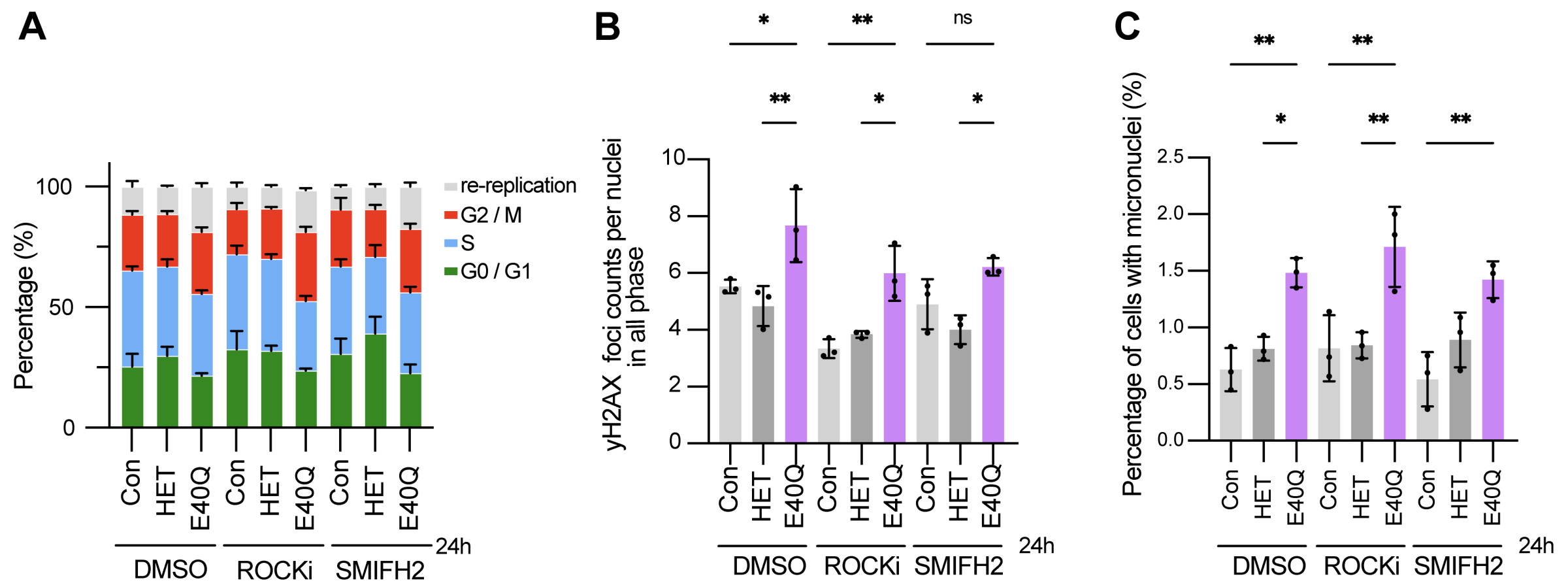

Supplemental Figure 14. RhoA E40Q regulates genome maintenance in the absence of RhoA WT independent of ROCK and DIAPH. (A) Cell cycle distribution of primary murine keratinocytes from wildtype (Con), *Rhoa* HET + *RHOA* E40Q (HET), and *Rhoa* KO + *RHOA* E40Q (E40Q) mice treated with DMSO, ROCK inhibitor Y-27632 (ROCKi, 10 µM), or SMIFH2 (10 µM) for 24 h. Cells were classified into G0/G1, S, G2/M, and re-replication phases based on DNA content and EdU incorporation. **(B)** Quantification of γH2AX foci in primary murine keratinocytes treated with DMSO, ROCKi, or SMIFH2 for 24 h (n:3). **(C)** Quantification of percentage of cells with micronuclei in primary murine keratinocytes treated with DMSO, ROCKi, or SMIFH2 for 24 h (n:3). All data are presented as mean ± SD. Statistical significance was determined using two-way ANOVA.

Supplemental Figure 15

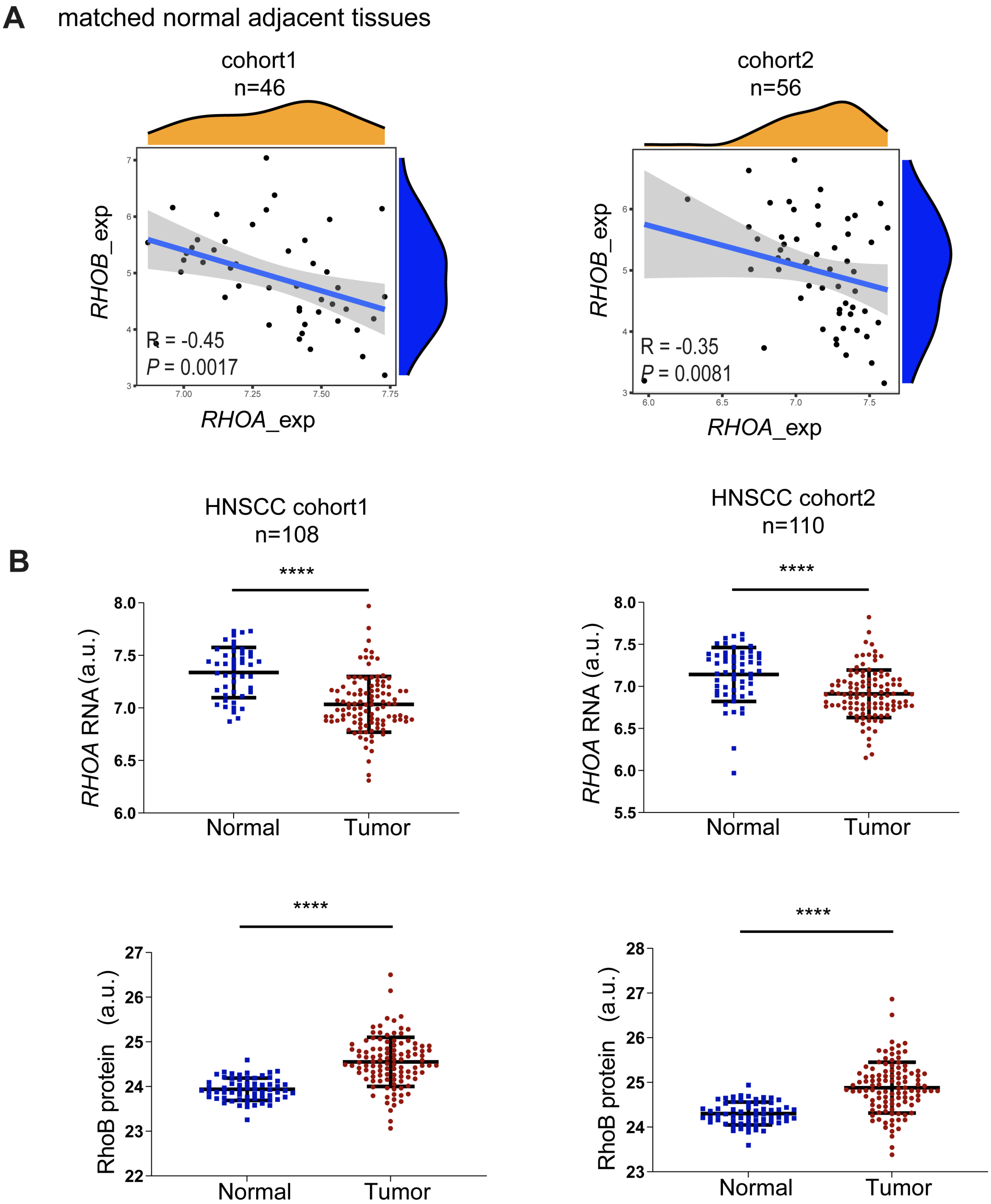

Supplemental Figure 15. Expression of *RHOA* and *RHOB* in HNSCC tumor and normal tissue. (A) Correlation of *RHOA* and *RHOB* expression in HNSCC tumor transcriptomic data. **(B)** *RHOA* RNA expression (upper panel) and RhoB protein level (lower panel) in tumor tissue in two independent HNSCC cohorts.

Supplemental Figure 16

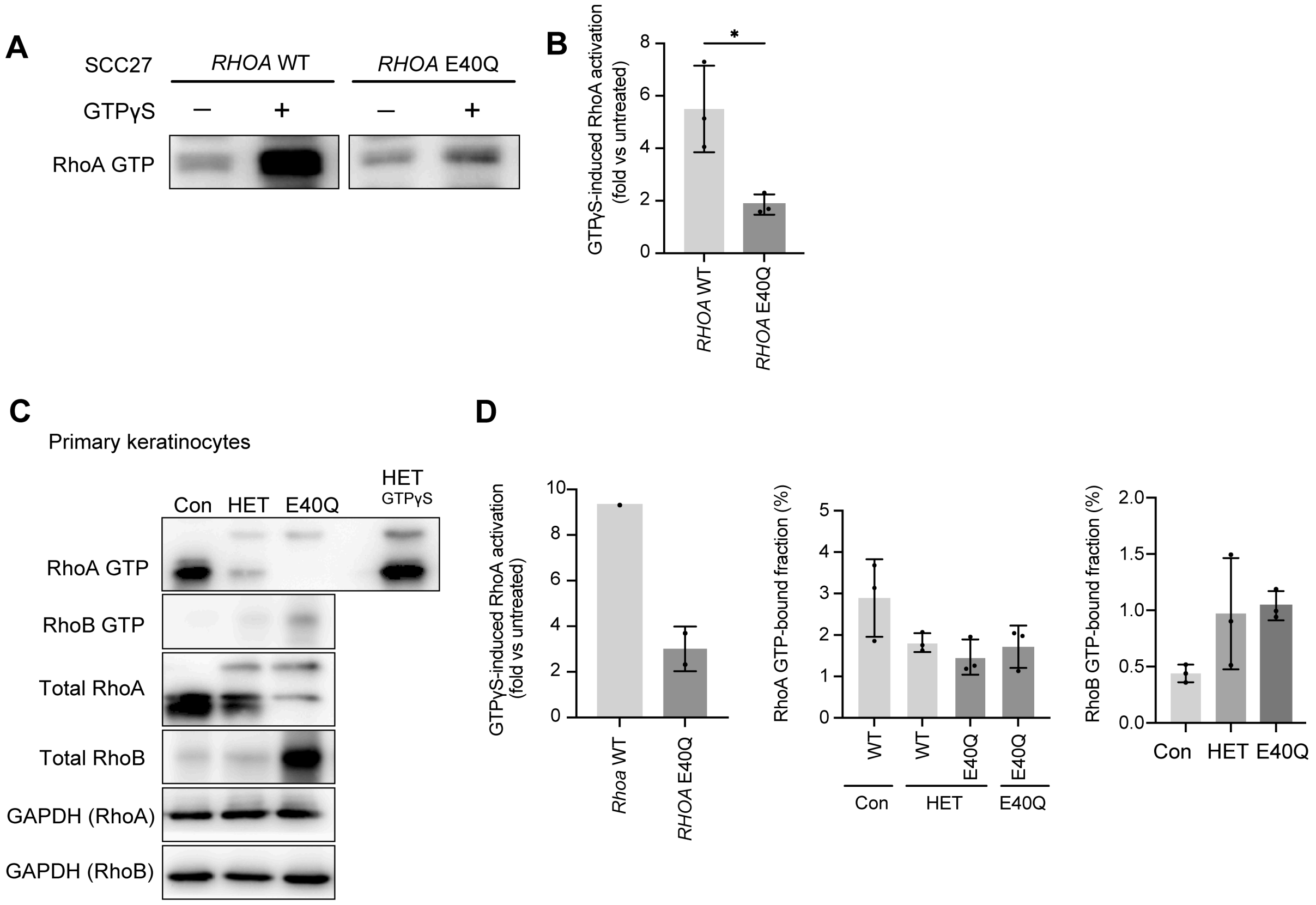

Supplemental Figure 16. RhoA E40Q exhibits reduced responsiveness to GTPγS stimulation. (A-B) Lysates of SCC27 cells expressing *RHOA* WT or *RHOA* E40Q were incubated with or without GTPγS, which irreversibly locks RhoA in the GTP-bound form. GTP-bound RhoA was then precipitated using Rhotekin-RBD beads. **(A)** Representative SCC27 RhoA pull-down assays. **(B)** Quantification of GTPγS-induced RhoA activation expressed as fold change relative to untreated cells. Data are presented as mean ± SD. Each dot represents one independent experiment**.** Statistical significance was determined using an unpaired two-tailed Student's t test. (n:3) **(C-D**) RhoA and RhoB pull-down assays with primary murine keratinocytes isolated from Control (Con), *Rhoa* HET + *RHOA* E40Q (HET), and *Rhoa* KO + *RHOA* E40Q mice (E40Q). Data are presented as mean ± SD. Each dot represents one mouse. Statistical significance was determined using one-way ANOVA, (n:3). **(C)** Representative pull-down assays showing GTP-bound RhoA and RhoB, total RhoA, total RhoB, and GAPDH loading controls. A GTPγS-treated HET sample is included as a positive control. **(D)** Quantification of the GTPγS-induced RhoA activation relative to untreated cells, RhoA GTP-bound fraction, and RhoB GTP-bound fraction.

Supplemental Figure 17

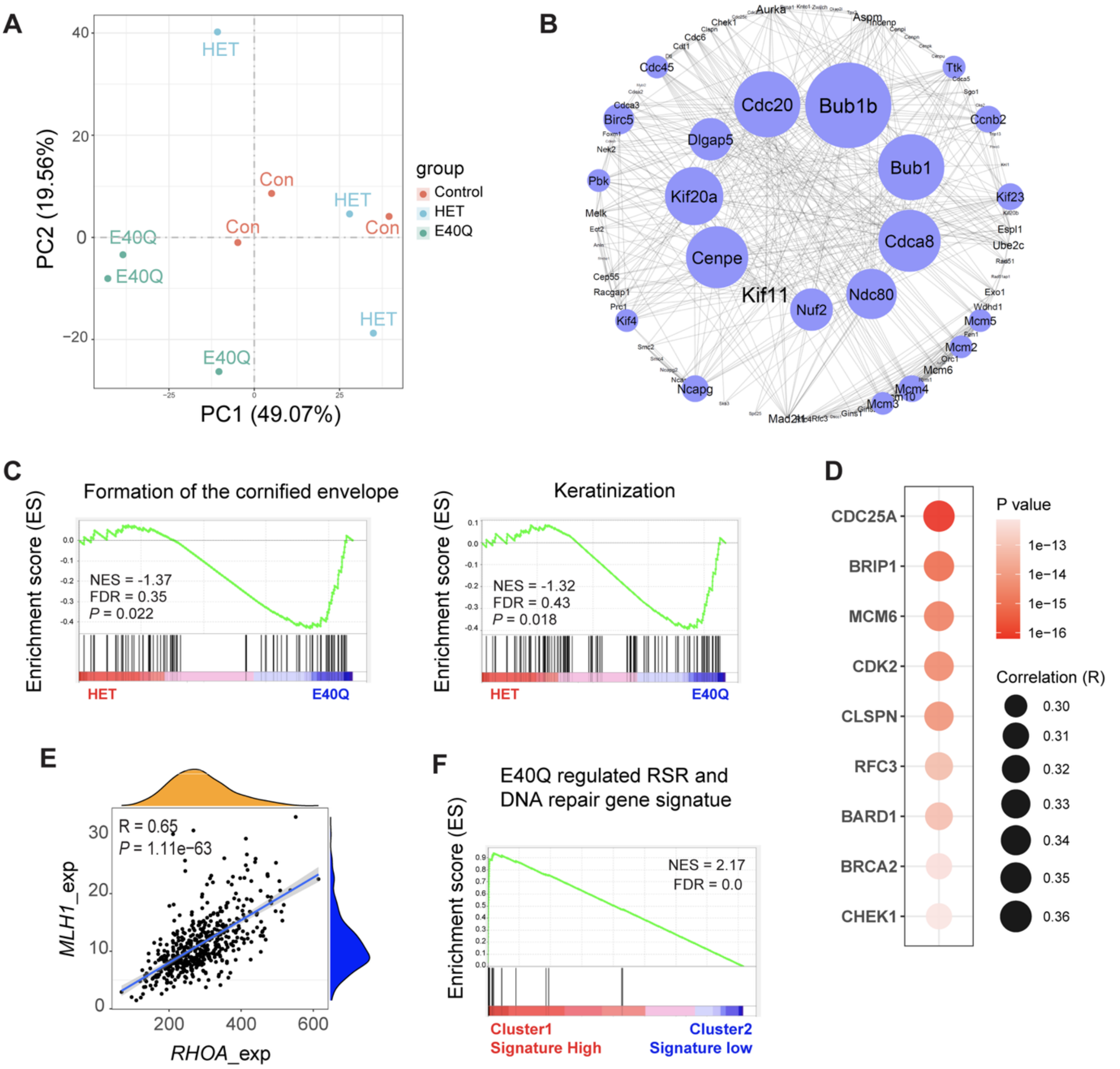

Supplemental Figure 17. Transcriptomic analyses of E40Q primary keratinocytes cells and HNSCC tumor. (A) PCA plot among replicates of wildtype (Control), *Rhoa* HET + *RHOA* E40Q (HET), and *Rhoa* KO + *RHOA* E40Q (E40Q). **(B)** Protein–protein interaction (PPI) network of the largest subnetwork derived from RNA-seq differentially expressed genes (DEGs) following MCODE clustering analysis. Nodes located at the center represent hub genes identified by CytoNCA analysis. **(C)** Gene set enrichment analysis (GSEA) of signatures comparing HET versus E40Q primary murine keratinocytes. **(D)** Correlation of genes *RHOA* expression with indicated genes in HNSCC tumor transcriptomic data. **(E)** Association of *RHOA* expression with *MLH1* in HNSCC tumor transcriptomic data. **(F)** Gene set enrichment analysis (GSEA) confirming the indicated signature significantly highly expressed in the cluster1.

Supplemental Tables

Supplemental Table 1

Functional gene group	Gene name	Amp (%)	Hom del (%)		Functional gene group	Gene name	Amp (%)	Hom del (%)
**Rho effectors**	*ROCK1*	1,8	0,2		**Rho GAPs**	*ARAP2*	0,4	0,2
	ROCK2	0	0			*ARHGAP1*	1,6	0
	DIAPH1	0	0,2			*ARHGAP5*	0,8	0,2
	DIAPH2	1,2	5			*ARHGAP6*	0,4	2
	DIAPH3	0,2	0,8			*ARHGAP8*	0	0
	RHPN1	8,0	0			*ARHGAP10*	0,4	0,4
	RHPN2	1	0,2			*ARHGAP11A*	0,4	0
	RTKN	0,2	0,6			*ARHGAP11B*	0,4	0
	RTKN2	0,8	0			*ARHGAP19*	0	0,4
	PKN1	0,4	0			*ARHGAP20*	0	1,2
	PKN2	0	0			*ARHGAP21*	0,4	0,6
	PKN3	0,8	0			*ARHGAP22*	0,8	0,2
	CIT	0,6	0			*ARHGAP23*	0,6	0
	KTN1	1	0			*ARHGAP26*	0	0,6
	FMNL1	0,2	0			*ARHGAP28*	1,8	0
						ARHGAP29	0	0
**Rho GTPases**	*RHOA*	0	0,2			*ARHGAP30*	0,8	0
	RHOB	0,2	0			*ARHGAP31*	2,8	0
	RHOC	0	1,6			*ARHGAP35*	0	0
	RAC1	1,8	0			*ARHGAP40*	0,6	0
	CDC42	0	0			*DLC1*	0	3
						GMIP	0,4	0
**GDIs**	*GDI1*	3	0			*MYO9A*	0,2	0
	GDI2	0,2	0,6			*MYO9B*	0,2	0
	ARHGDIG	0,2	0			*STARD8*	1	0,8
						STARD13	0,2	0,2
**Rho GEFs**	*AKAP13*	0,2	0,2			*TAGAP*	0	0,6
	ARHGEF1	0,2	0					
	ARHGEF2	0,2	0		**Contraction**	*MYLK*	2,8	0
	ARHGEF3	0	0,8			*PPP1R12A*	0,2	0,2
	ARHGEF4	0,2	0			*MYH9*	0	0,2
	ARHGEF5	0,2	0,2			*MYH10*	0	0,2
	ARHGEF10	0	4			*MYH14*	0,2	0
	ARHGEF11	0,6	0			*MYL6*	0,2	0
	ARHGEF12	0	0,8			*MYL6B*	0,2	0
	ARHGEF15	0	0			*MYL9*	1,6	0
	ARHGEF17	4	0,4			*MYL12A*	4	0
	ARHGEF19	0	0,2			*MYL12B*	4	0
	ARHGEF25	0,4	0					
	ARHGEF28	0,2	0,2					
	ECT2	14	0					
	MCF2	1,4	0,2					
	MCF2L	1,8	0,2					
	NET1	0,2	0,6					
	NGEF	0	1,6					
	PLEKHG5	0,4	0,6					
	PLEKHG6	1,2	0					
	PREX2	2,8	0					
	RASGRF2	0,1	0,2					
	VAV1	0	0,4					
	VAV2	1	0,2					
	VAV3	0	0,8					

Supplemental Table 2

A

**Variants targeted**	**crRNA sequences**
RhoA KO (K6*)	5’ CACAGTGTTTGAGAACTATG 3’
RhoA E40Q	5’ CAACAATCACCAGTTTCTTC 3’

B

<table>
<tr><th></th><th>ssODNs repair template sequences</th></tr>
<tr><td rowspan="4">RhoA E40Q</td><td>MUT</td></tr>
<tr><td>5’TTCAGCAAGGACCAGTTCCCAGAGGTGTATGTGCCCACAGTGTTTCAGAACTATGTCGCA GATATCGAGGTGGATGGAAAGCAGGTGAGTATA 3’</td></tr>
<tr><td>WT’</td></tr>
<tr><td>5’TTCAGCAAGGACCAGTTCCCAGAGGTGTATGTGCCCACAGTGTTTGAAAACTATGTCGCA GATATCGAGGTGGATGGAAAGCAGGTGAGTATA 3’</td></tr>
<tr><td rowspan="4">RhoA KO (K6*)</td><td>MUT</td></tr>
<tr><td>5’CAGGTAATATCTGTGTTTTGTGTTTCAGCAATGGCTGCCATCCGGTAGAAACTGGTGATT GTTGGTGATGGAGCCTGTGGAAAGACATGCTTG 3’</td></tr>
<tr><td>WT’</td></tr>
<tr><td>CAGGTAATATCTGTGTTTTGTGTTTCAGCAATGGCTGCCATCCGGAAAAAACTGGTGATTGT TGGTGATGGAGCCTGTGGAAAGACATGCTTG</td></tr>
<tr><td rowspan="4">RhoA E40Q</td><td>MUT</td></tr>
<tr><td>5’TTCAGCAAGGACCAGTTCCCAGAGGTGTATGTGCCCACAGTGTTTCAGAACTATGTCGCA GATATCGAGGTGGATGGAAAGCAGGTGAGTATA 3’</td></tr>
<tr><td>WT’</td></tr>
<tr><td>5’TTCAGCAAGGACCAGTTCCCAGAGGTGTATGTGCCCACAGTGTTTGAAAACTATGTCGCA GATATCGAGGTGGATGGAAAGCAGGTGAGTATA 3’</td></tr>
</table>

Supplemental Table 3

	PCR Primer for CRSPR select
	RhoA KO (K6*)
**Forward**	5’ ATGTGTCATCCTGTAAGCTACCT 3’
**Reverse**	5’ TATACTCACCTGCTTTCCATCCA 3’
**Adaptor+ forward**	5’ ACACTCTTTCCCTACACGACGCTCTTCCGATCTATGTGTCATCCTGTAAGCTACCT 3’
**Adaptor+ reverse**	5’ TGACTGGAGTTCAGACGTGTGCTCTTCCGATCTTATACTCACCTGCTTTCCATCCA 3’
	RhoA E40Q
**Forward**	5’ GACATGCTTGCTCATAGTCTTCA 3’
**Reverse**	5’ GCTCTAATTCTCTACATGCTCCA 3’
**Adaptor+ forward**	5’ ACACTCTTTCCCTACACGACGCTCTTCCGATCTGACATGCTTGCTCATAGTCTTCA 3’
**Adaptor+ reverse**	5’ TGACTGGAGTTCAGACGTGTGCTCTTCCGATCTGCTCTAATTCTCTACATGCTCCA 3’

Supplemental Methods

Immunofluorescence staining of cell lines

Cells were seeded on 8-well Permanox slides (Thermo Fisher Scientific, #177445), glass slides (Lab-Tek, Thermo Fisher Scientific, #154534), or on round coverslips (VWR, #631-1578) in 6-well plates the day before the experiment. Cells were fixed in 4% paraformaldehyde (PFA) in PBS (v/v) for 15 min at RT, followed by three times 5min PBS washes. Permeabilization and blocking were performed in PBS containing 3% BSA and 0.1% saponin or 0.1% Triton X-100 in PBS (v/v) for 20 min. Primary antibodies were incubated for 1 h at RT in blocking buffer, followed by 1 h incubation with secondary antibodies at RT in the dark. Nuclei were stained with DAPI (Thermo Fisher Scientific, #D1306. 1:1000 dilution) for 10 min at RT.

The following primary antibodies were used: rabbit anti-YAP (Cell Signaling Technology, #14074, 1:100), rabbit anti-β-catenin (Santa Cruz, #sc-7199, 1 :200), rabbit anti-E-cadherin (Cell Signaling Technology, #3195, 1:200), mouse anti-Myc-Tag (9B11) (Cell Signaling Technology, #2276, 1:200), mouse anti-HA-Tag (6E2) (Cell Signaling Technology, #2367, 1:200) or rabbit anti-HA-Tag (C29F4) (Cell Signaling Technology, #3724, 1:200), and rabbit anti-phospho-Histone γH2AX (Ser139) (Cell Signaling Technology, #2577, 1:500). The following secondary antibodies were used: goat anti-rabbit Alexa Fluor 488 (Thermo Fisher Scientific, #A-11034, 1:400), goat anti-mouse Alexa Fluor 488 (Thermo Fisher Scientific, #A-11001, 1:400), goat anti-rabbit Alexa Fluor 568 (Thermo Fisher Scientific, #A-11011, 1:500), and goat anti-mouse Alexa Fluor 568 (Thermo Fisher Scientific, #A-11031, 1:1000). Alexa Fluor 647–conjugated phalloidin (Thermo Fisher Scientific, #A22287, 1:1000) was used to detect F-actin. Samples were mounted in fluorescence mounting medium (DAKO, Agilent Technologies, #S3023), sealed with nail polish when indicated, and stored at 4 °C. Imaging was performed on the Zeiss confocal LSM800 microscope (40X or 63X). Quantification was performed using CellProfiler (1). The nuclear fraction was calculated by dividing the integrated intensity in the nucleus by the integrated intensity in the whole cell.

CRISPR-Select assay

The CRISPR-Select assay was conducted as described earlier (20). crRNAs mediating cutting within 10 bp of the site of mutation for KO (K6*), E40Q, or a silent wild-type mutation (WT’) were designed using Benchling (Supplemental Table 2A). ssODN repair templates contained

the respective test mutations (*RHOA* KO (K6*), *RHOA* E40Q, or a silent mutation (WT')), homologous arms of 45 nt (Supplemental Table 2B), and a silent mutation in the crRNA seed region to minimize recutting. To induce Cas9 expression in iCas9-MCF10A, 1 µg/ml doxycycline (Sigma-Aldrich, #D9891) was added to the culture medium 24 h prior to transfection with crRNA, trans-activating CRISPR RNA (tracrRNA), and ssODNs using RNAiMAX (Thermo Fisher Scientific, #13778150). Cells were seeded into 10 cm dishes and maintained for 12 days, with passaging performed when confluency reached around 80%. The medium with 10 µM ROCKi was refreshed every two days. The percentages of test mutation to silent WT' mutation were determined at 2 and 12 days after transfection by a two-step protocol PCR protocol for amplicon NGS.

In the first-round PCR, primers specific to the target site included overhangs with binding sites for second-round primers using Phusion U Green Multiplex PCR Master Mix (Thermo Fisher Scientific, #F564L). In the second-round PCR, primers included overhangs with both sample-specific barcodes and adaptors required for NGS (Supplemental Table 3). Subsequently, the amplicon sequencing library was prepared with the MiSeq Reagent Kit v2 (Illumina, #MS-102-2002) and sequencing was carried out on an Illumina MiSeq system following the manufacturer's protocol. Sequencing depth per sample ranged from 20,000 to 200,000 reads. The resulting NGS data were analyzed using the CRISPResso2 online tool.

Rho activation analysis by RhoA pull-down assay

Primary murine keratinocytes were seeded in 10 cm dishes. The culture medium was replaced the following day, and cells were harvested 48 h after plating. RhoA and RhoB activity assays were performed using a RhoA Pull-Down Activation Assay Biochem Kit (Cytoskeleton, #BK036) according to the manufacturer's instructions. Briefly, 200 µg of total cell lysate was incubated with 15 µL of Rhotekin-RBD agarose bead slurry for 60 min at 4°C to precipitate GTP-bound Rho proteins. Beads were collected by centrifugation and washed according to the manufacturer's protocol. Pulled-down proteins and corresponding input lysates (10 µg) were analyzed by western blotting using anti-RhoA or anti-RhoB antibodies as described above. GTPγS-loaded lysates supplied with the kit were used as positive controls. RhoA activity was quantified using Fiji as the percentage of GTP-bound RhoA relative to total RhoA in the corresponding input lysate.

Protein-protein interaction (PPI) network analysis

Differentially expressed genes identified in *RhoA* E40Q homozygous keratinocytes were submitted to the STRING database (https://string-db.org/) using the highest confidence threshold (interaction score ≥ 0.900) to construct PPI networks. Networks were imported into Cytoscape (v3.10.2) (2), and subnetworks were identified using the MCODE plugin. Interaction centrality scores were analyzed using the CytoNCA plugin. Hub genes were identified and ranked with the Maximal Clique Centrality (MCC) algorithm implemented in the CytoHubba plugin. PPI networks were visualized in Cytoscape, with node size scaled according to interaction degree derived from the calculated scores.

References

1. Stirling DR, et al. CellProfiler 4: improvements in speed, utility and usability. *BMC Bioinformatics.* 2021;22(1):433.
2. Shannon P, et al. Cytoscape: a software environment for integrated models of biomolecular interaction networks. *Genome Res.* 2003;13(11):2498-504.
