# Supplementary material for: RhoA WT allele loss unleashes the cancer driver function of RhoA E40Q
Source: J Clin Invest. 2026 Aug 25;136(20):e201975. doi: 10.1172/JCI201975 (PMC13668625; doi:10.1172/JCI201975)

Original unedited blots

Figure 1A

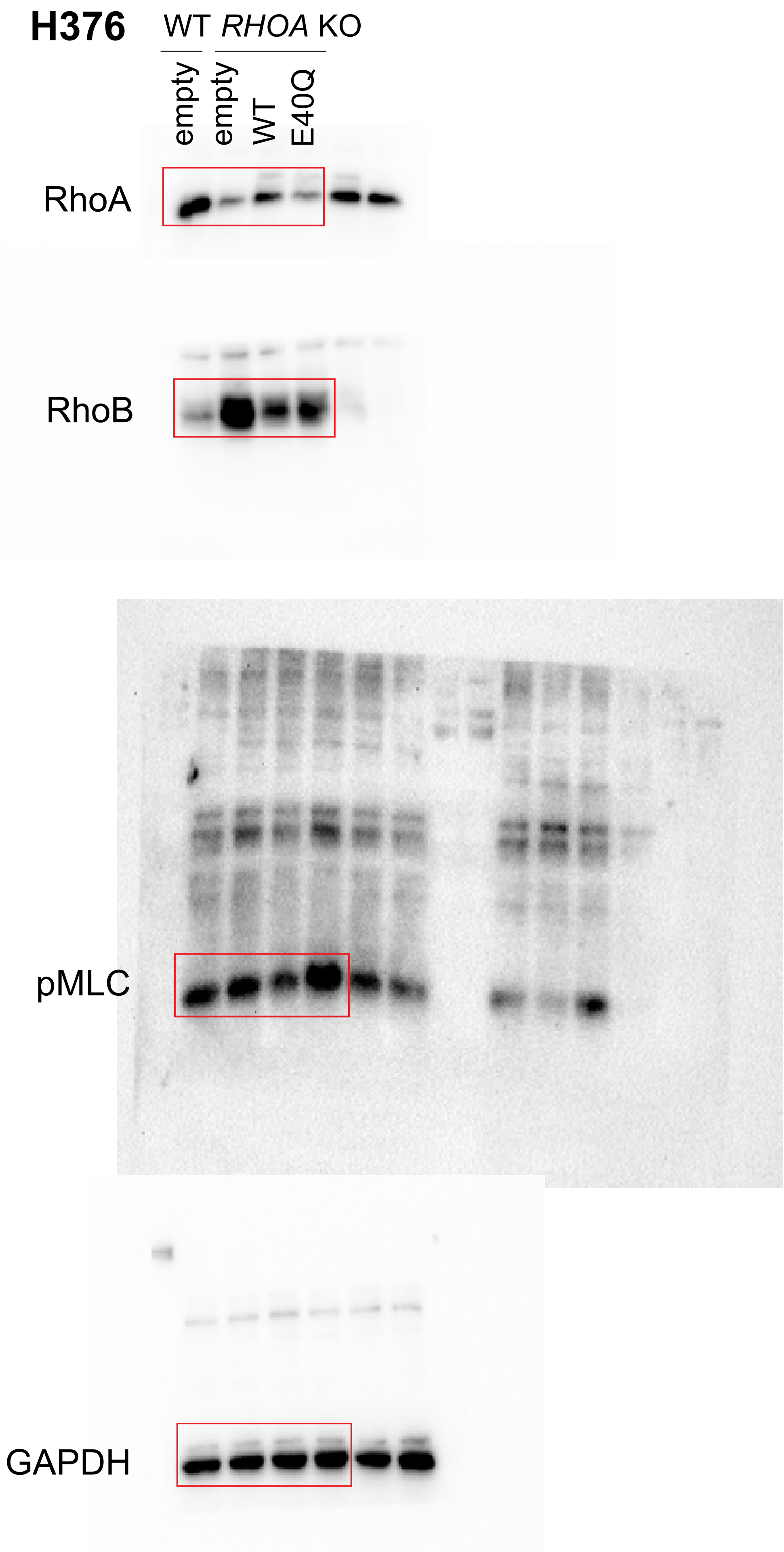

Figure 1A

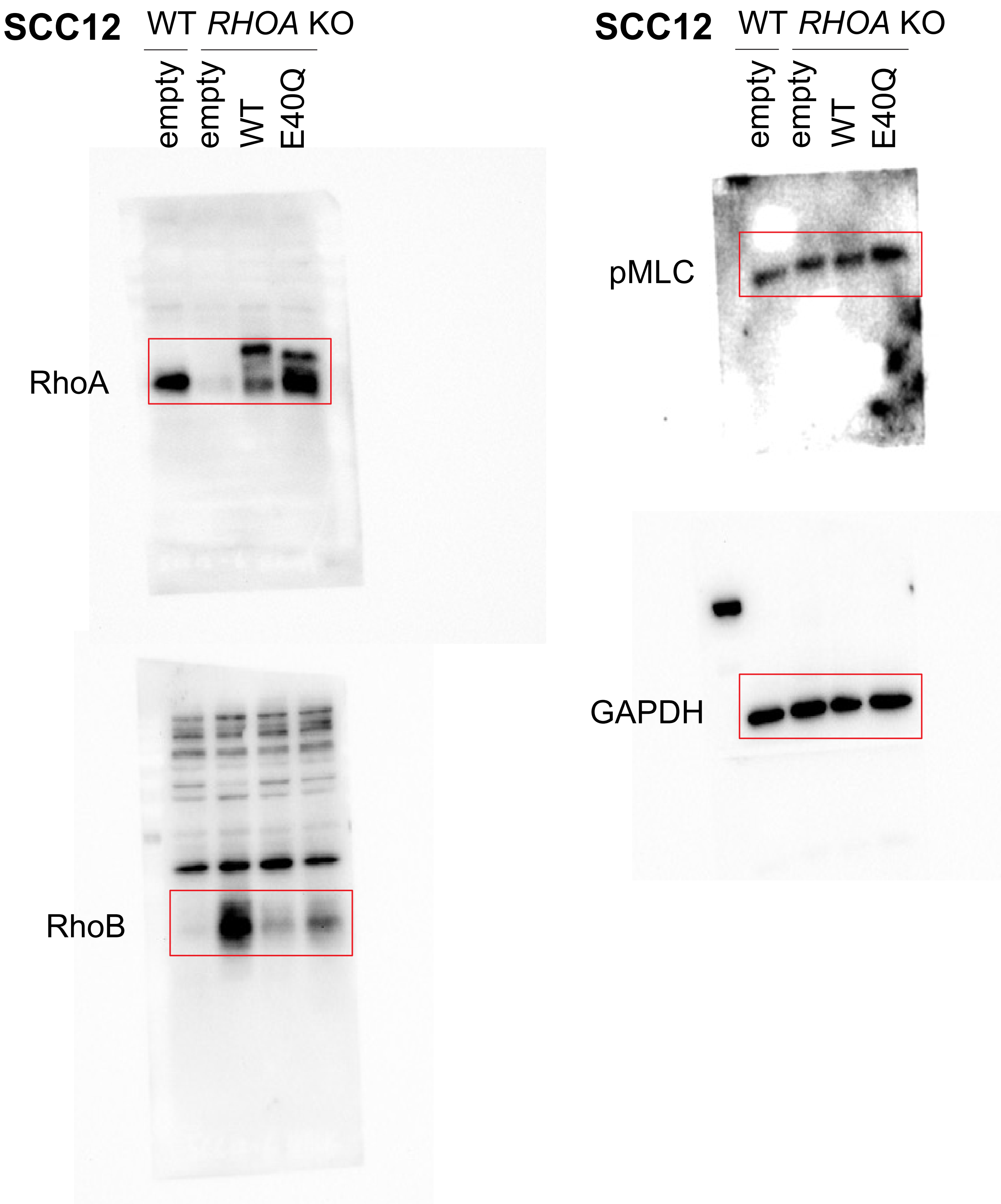
SCC12
WT
RHOA KO
empty
empty
WT
E40Q
RhoA
RhoB
SCC12
WT
RHOA KO
empty
empty
WT
E40Q
pMLC
GAPDH

Figure 1A

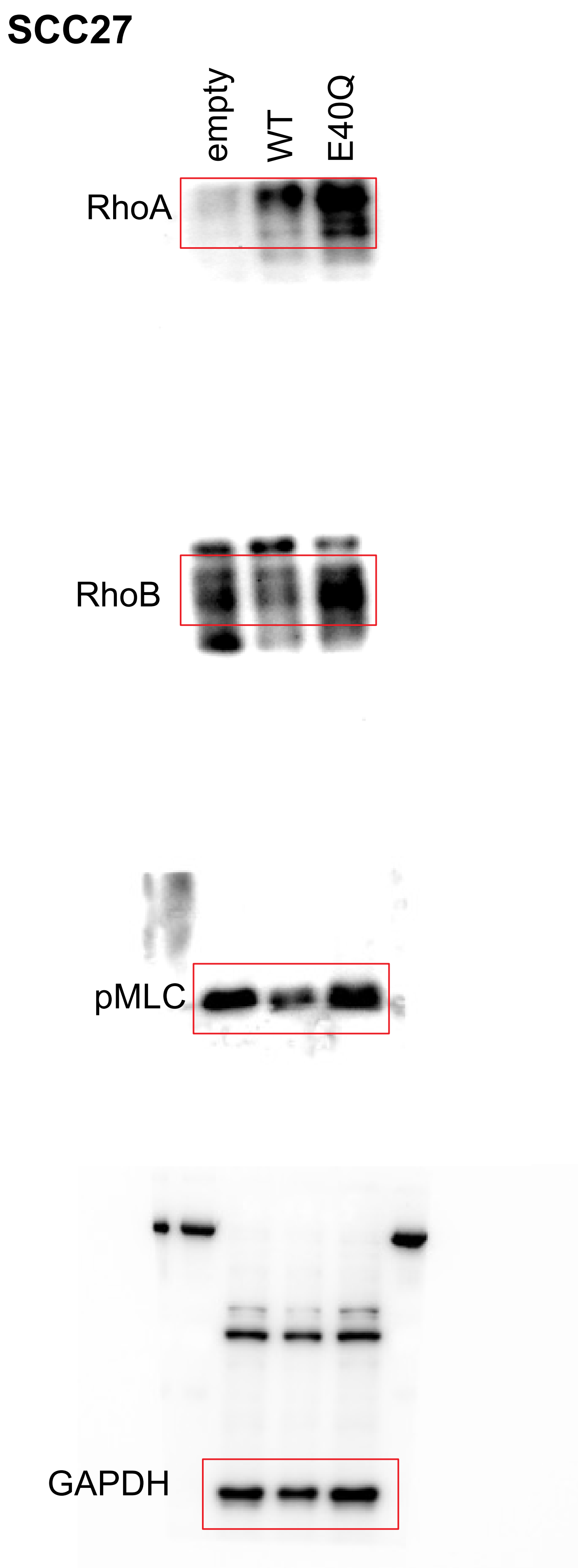

Figure 1A

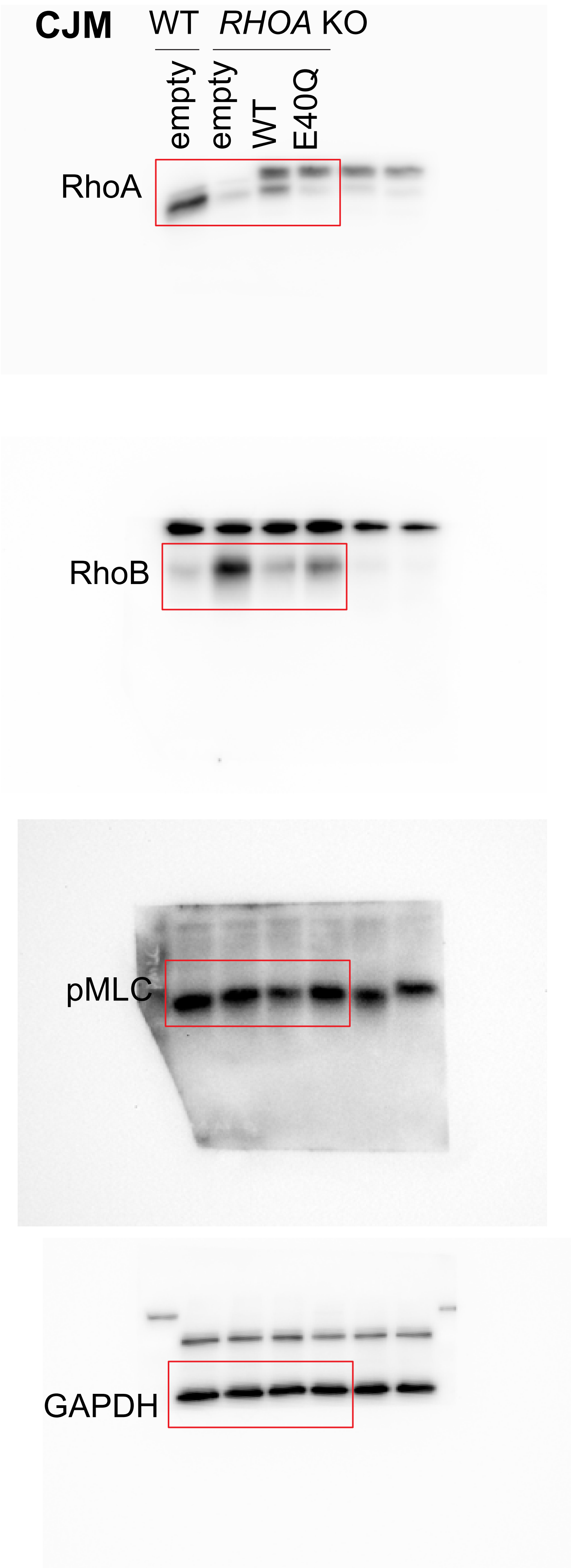

Figure 1C

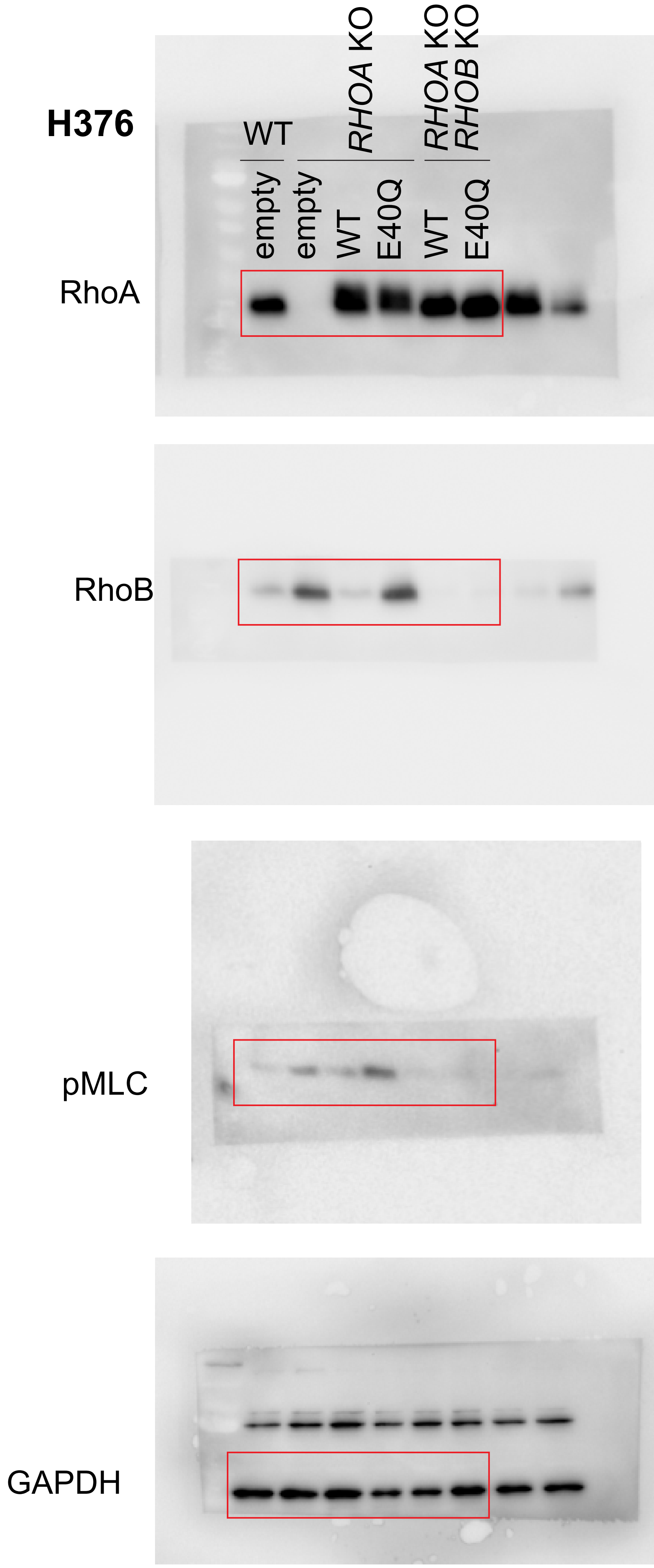
H376
WT
RHOA KO
RHOA KO
RHOB KO
empty
empty
WT
E40Q
WT
E40Q
RhoA
RhoB
pMLC
GAPDH

Figure 1C

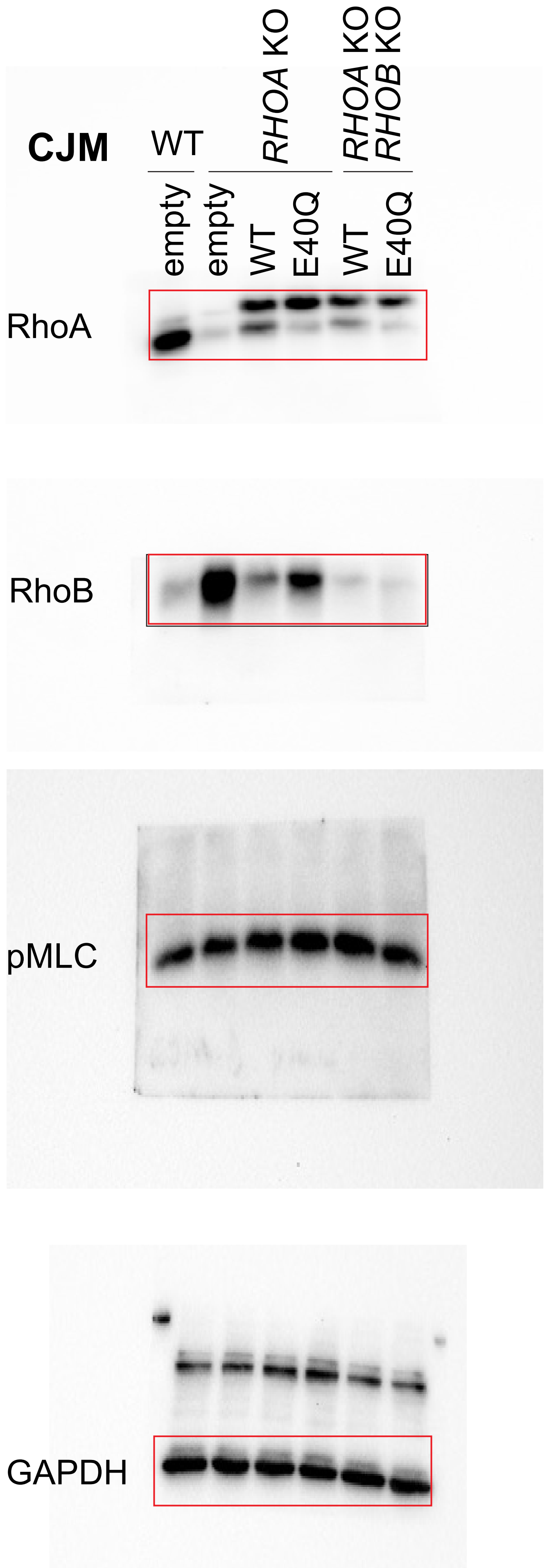
CJM
WT
RHOA KO
RHOA KO
RHOB KO
empty
empty
WT
E40Q
WT
E40Q
RhoA
RhoB
pMLC
GAPDH

Figure 8A

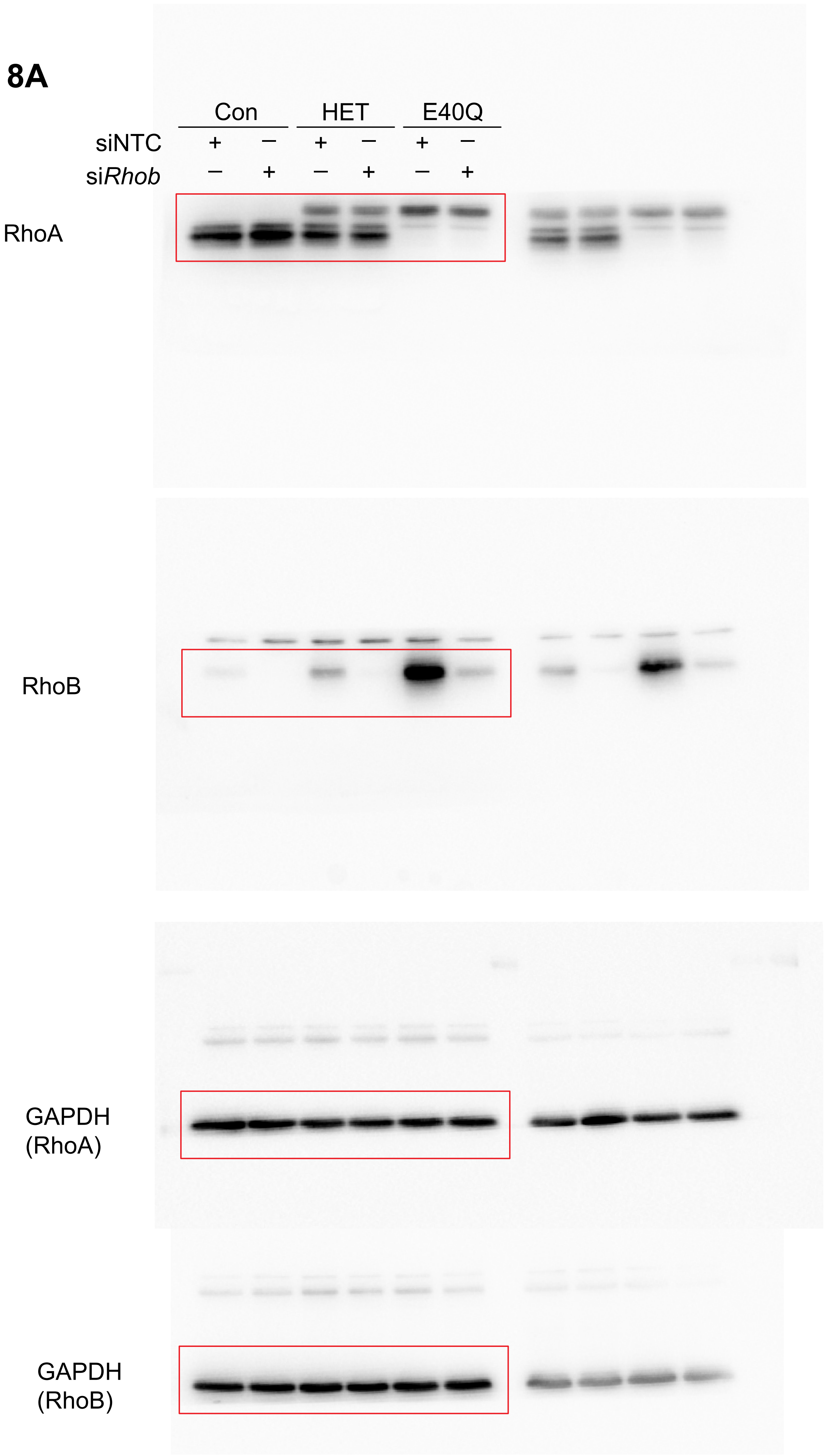

SFigure 2A

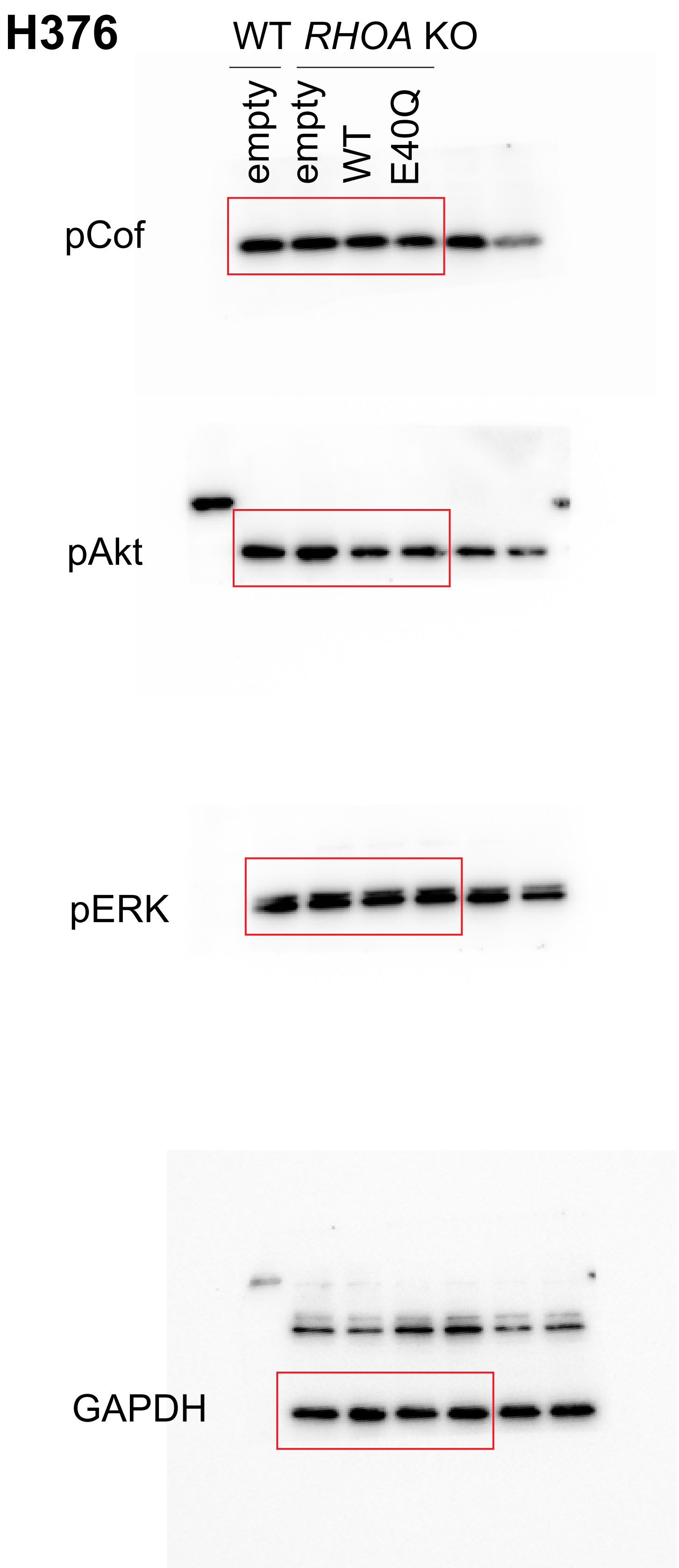

SFigure 2A

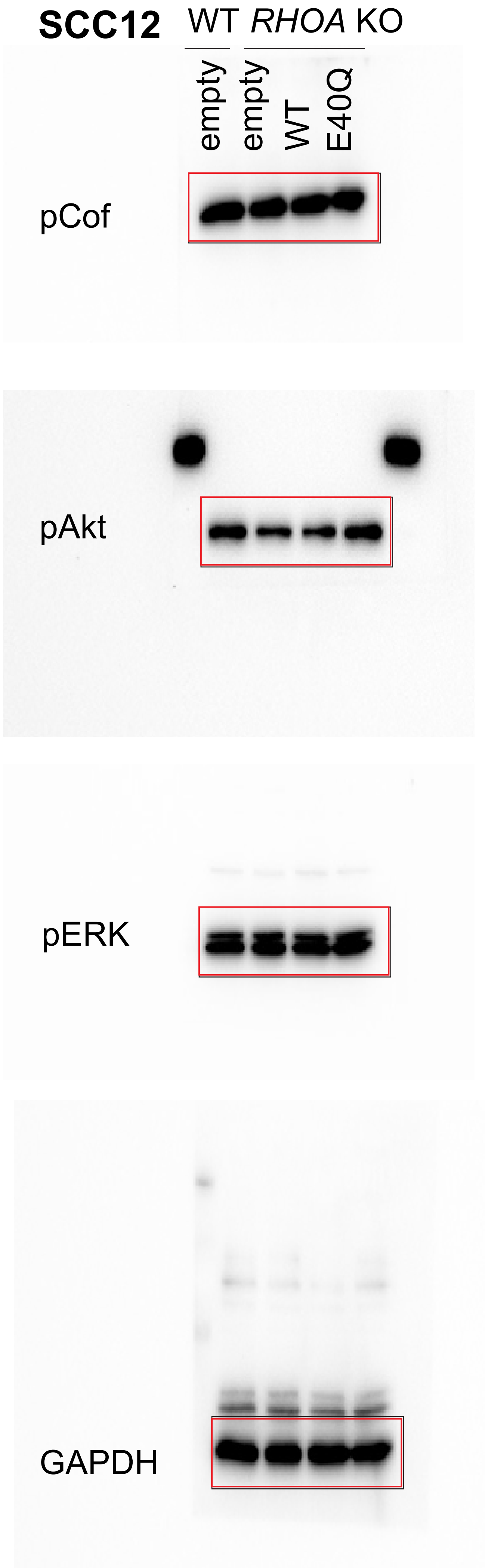

SFigure 2A

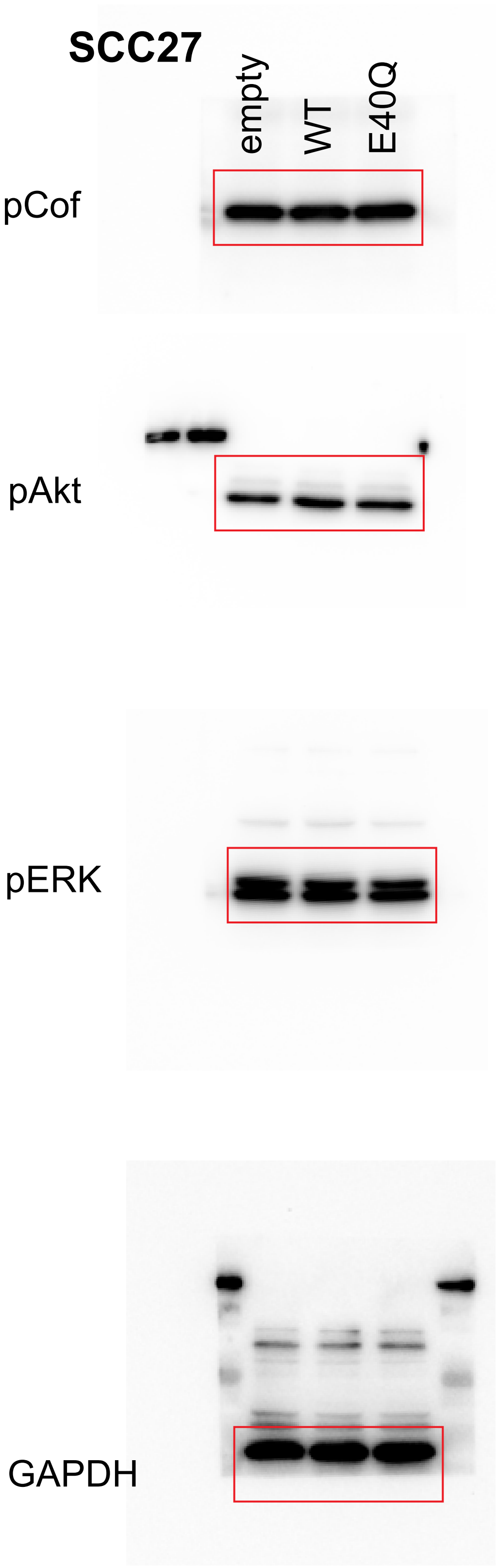

SFigure 2A

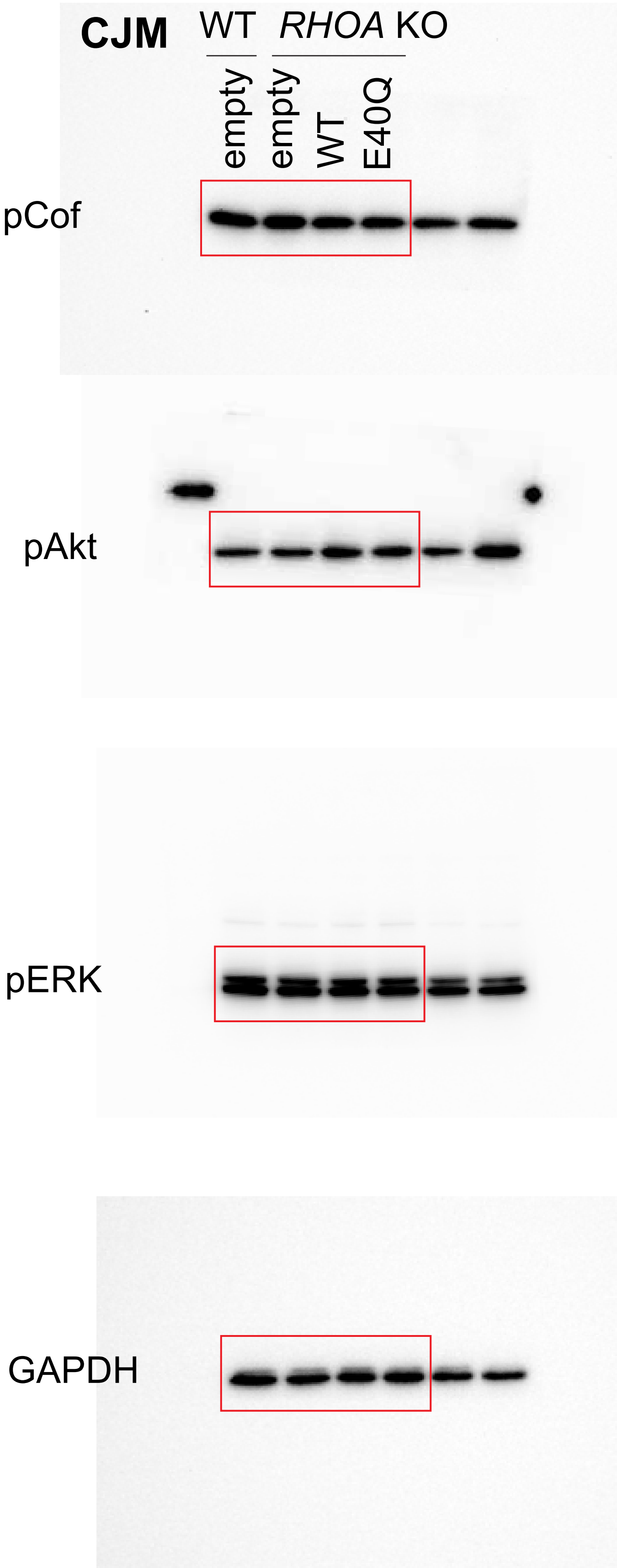

SFigure 2B

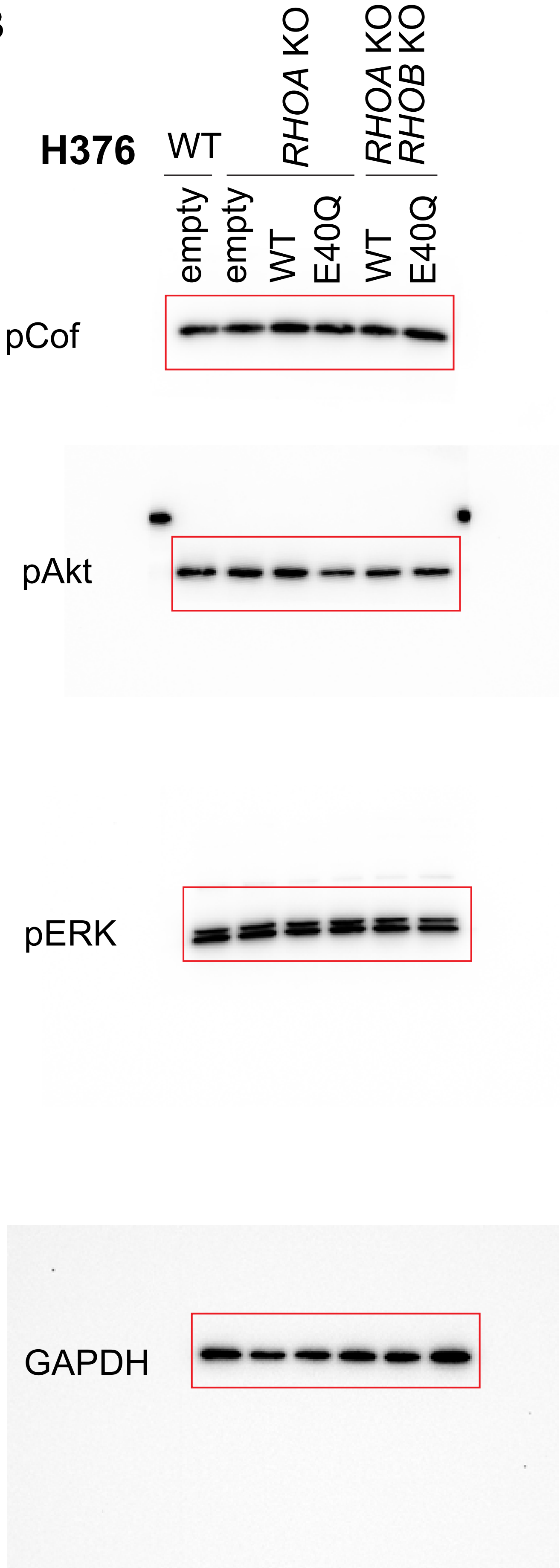
H376
WT
RHOA KO
RHOA KO
RHOB KO
empty
empty
WT
E40Q
WT
E40Q
pCof
pAkt
pERK
GAPDH

SFigure 2B

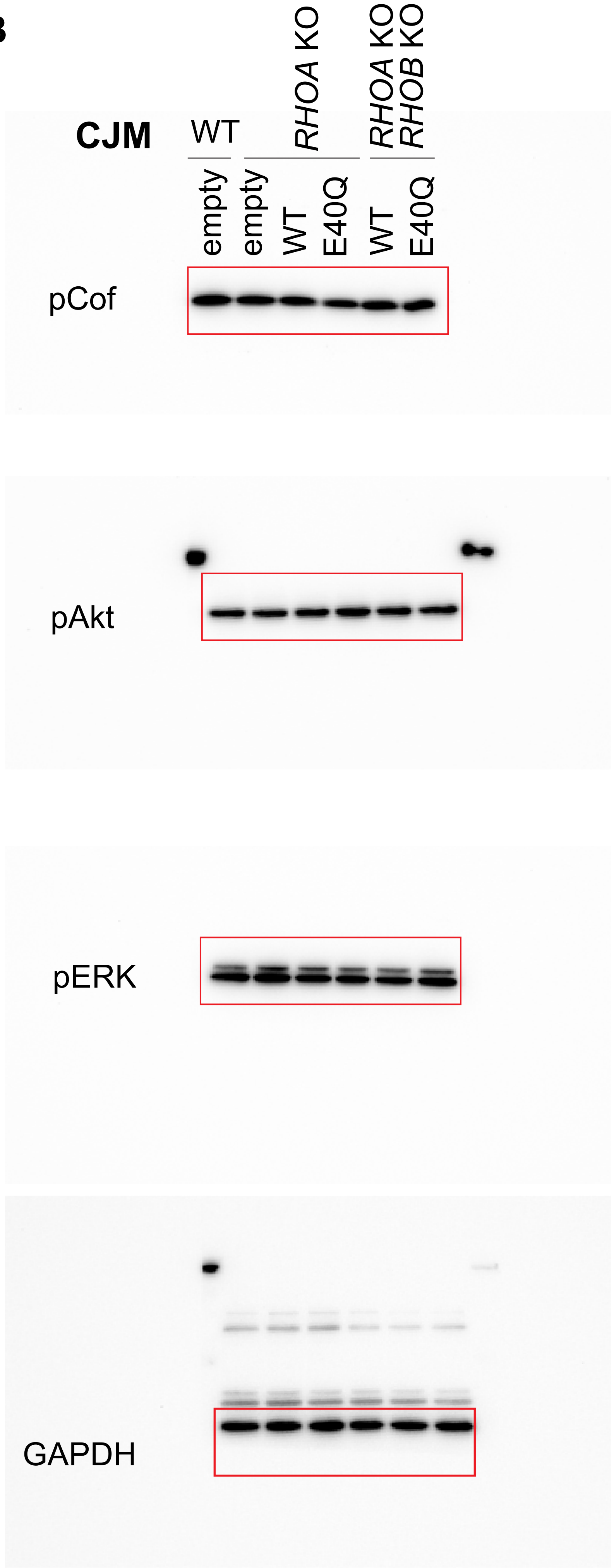
CJM
WT
RHOA KO
RHOA KO
RHOB KO
empty
empty
WT
E40Q
WT
E40Q
pCof
pAkt
pERK
GAPDH

SFigure 3

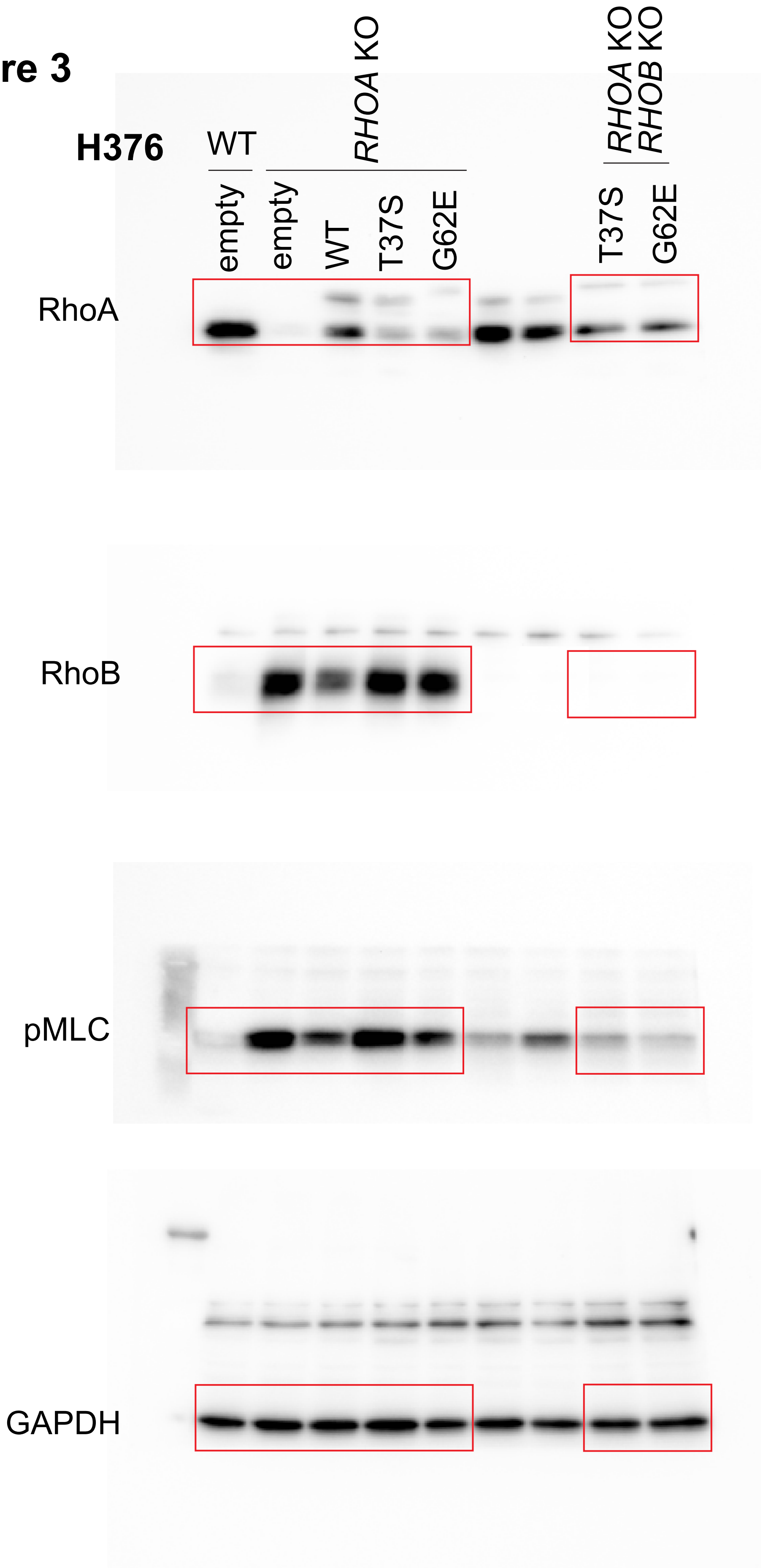
H376
WT
RHOA KO
RHOA KO
RHOB KO
empty
empty
WT
T37S
G62E
T37S
G62E
RhoA
RhoB
pMLC
GAPDH

SFigure 12

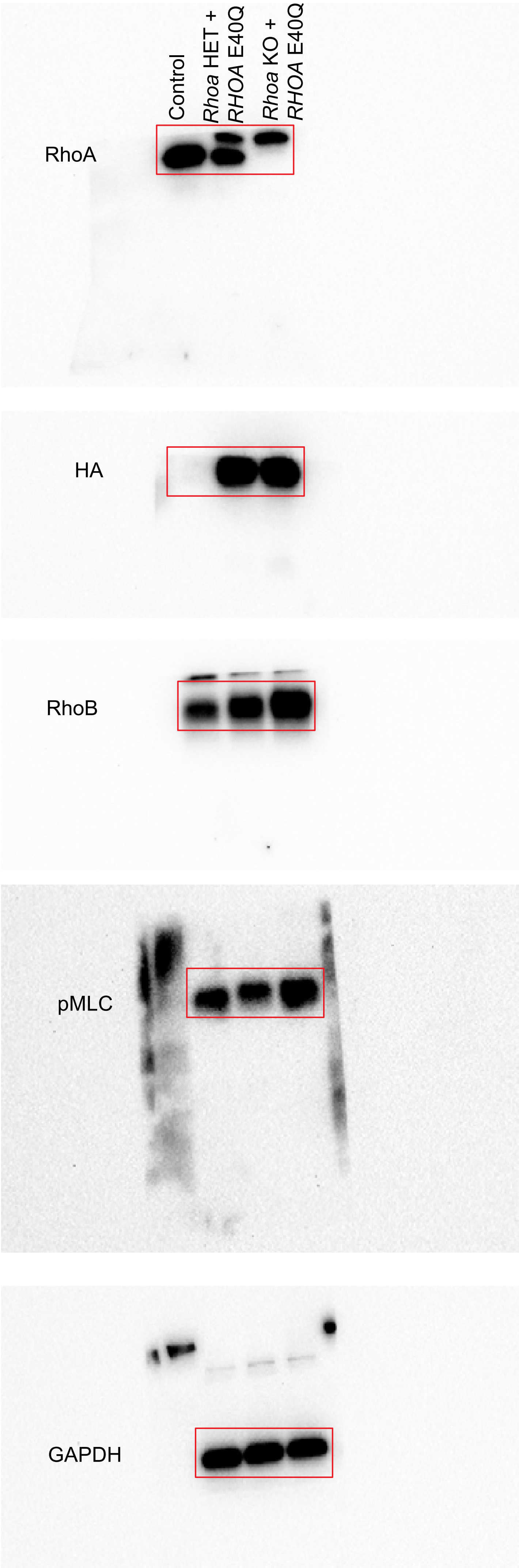

Control
Rhoa HET + *RHOA* E40Q
Rhoa KO + *RHOA* E40Q
RhoA
HA
RhoB
pMLC
GAPDH

SFigure 16A

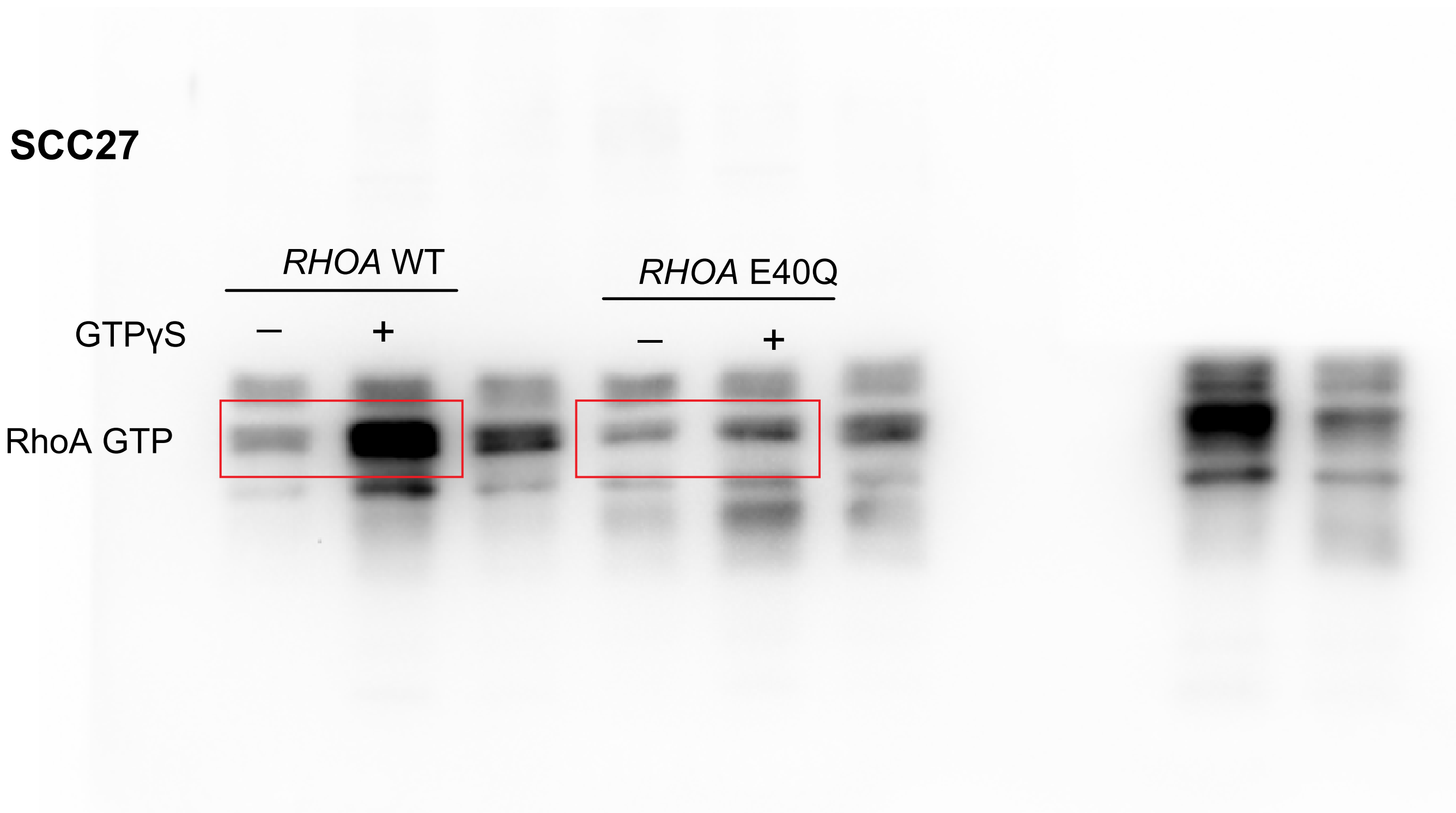

SFigure 16C

Primary keratinocytes

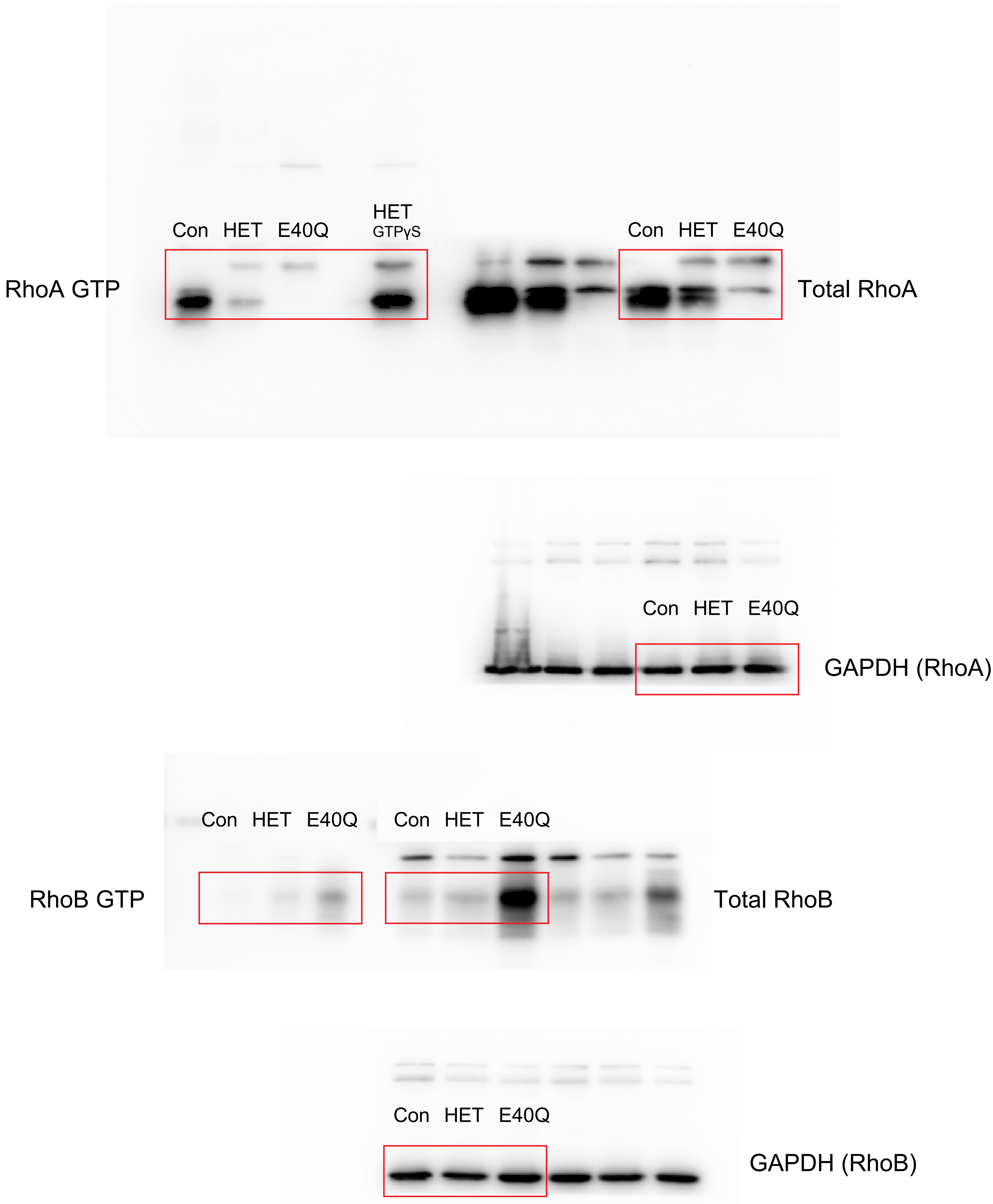

Supplement: Supplementary file 2 [file jci-136-201975-s153.pdf]
